# Synthesis of Sterically Shielded Nitroxides Using the Reaction of Nitrones with Alkynylmagnesium Bromides

**DOI:** 10.3390/molecules27217626

**Published:** 2022-11-07

**Authors:** Sergey A. Dobrynin, Mark M. Gulman, Denis A. Morozov, Irina F. Zhurko, Andrey I. Taratayko, Yulia S. Sotnikova, Yurii I. Glazachev, Yuri V. Gatilov, Igor A. Kirilyuk

**Affiliations:** 1 N. N. Vorozhtsov Novosibirsk Institute of Organic Chemistry SB RAS, Lavrentiev Ave. 9, 630090 Novosibirsk, Russia; 2 Department of Natural Sciences, Novosibirsk State University, Pirogova Str. 2, 630090 Novosibirsk, Russia; 3 Voevodsky Institute of Chemical Kinetics and Combustion SB RAS, Institutskaya 3, 630090 Novosibirsk, Russia

**Keywords:** nitroxides, nitrones, organometallic compounds

## Abstract

Sterically shielded nitroxides, which demonstrate high resistance to bioreduction, are the spin labels of choice for structural studies inside living cells using pulsed EPR and functional MRI and EPRI in vivo. To prepare new sterically shielded nitroxides, a reaction of cyclic nitrones, including various 1-pyrroline-1-oxides, 2,5-dihydroimidazole-3-oxide and 4*H*-imidazole-3-oxide with alkynylmagnesium bromide wereused. The reaction gave corresponding nitroxides with an alkynyl group adjacent to the N-O moiety. The hydrogenation of resulting 2-ethynyl-substituted nitroxides with subsequent re-oxidation of the N-OH group produced the corresponding sterically shielded tetraalkylnitroxides of pyrrolidine, imidazolidine and 2,5-dihydroimidazole series. EPR studies revealed large additional couplings up to 4 G in the spectra of pyrrolidine and imidazolidine nitroxides with substituents in 3- and/or 4-positions of the ring.

## 1. Introduction

Cyclic nitroxides with four ethyl (or more bulky alkyl) substituents adjacent to the N-O group are known to show much higher resistance to reduction with biogenic reductants and enzymatic systems than corresponding tetramethyl analogs [1]. The higher stability of these so-called “sterically shielded” nitroxides makes them favorable for those fields of application where the decay of conventional tetramethyl nitroxides is fast. The stability of the nitroxide group is of crucial importance for structural studies of biological macromolecules in their native environment inside living cells using site-directed spin labeling (SDSL) and pulsed EPR techniques [2]. For this application, reduction-resistant spin labels were prepared from sterically shielded nitroxides of pyrroline [3,4,5], isoindoline [6] and pyrrolidine series [7,8]. Sterically shielded nitroxides of piperidine [9,10] and imidazoline [11,12] series were designed for functional MRI and EPRI in vivo.

The reaction of cyclic nitrones with organometallic reagents is a widely used method of synthesis of various nitroxides [13,14]. There are some examples of successful synthesis of sterically shielded tetraethyl nitroxides using this method. For example, the direct addition of ethylmagnesium bromide to cyclic nitrones of 4*H*-imidazole 3-oxide or 2*H*-imidazole 1-oxide series afforded 2,2,5,5-tetraethyl substituted 2,5-dihydroimidazol-1-oxyls with 40–50% yield [15,16]. However, sterically hindered 1-pyrroline 1-oxides are not prone to the addition of EtMgBr [8,17], presumably due to metalation [18]. These nitrones, nevertheless, readily react with vinylmagnesium [19], allylmagnesium [20] or ethynylmagnesium halides [8,21], which are known to show lower basicity than EtMgBr, affording corresponding nitroxides in good yields. The terminal vinyl or ethynyl groups can then be converted into ethyl ones via hydrogenation. Although the nitroxide group is reduced to hydroxylamine, it can be easily recovered via oxidation. This technique of indirect introduction of the ethyl group allowed us to prepare highly resistant to reduction nitroxides [8,19,21].

**Figure 1:**
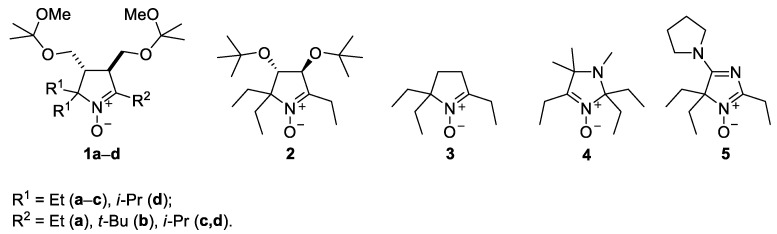
The sterically hindered nitrones used in this study.

In this work, we focus on reactions of alkynylmagnesium bromides with various cyclic nitrones of pyrroline (**1**–**3**), imidazoline (**4**) and 4*H*-imidazole-3-oxide (**5**) series (Figure 1).

The reaction was found to give satisfactory yields of 2-ethynyl-substituted nitroxides with all these sterically hindered nitrones except for **1d**. Subsequent hydrogenation of the ethynyl derivatives produced corresponding sterically shielded tetraalkylnitroxides.

Besides that, the new 2-alkynyl-substituted nitroxides themselves may be of interest as bioorthogonal spin labels capable of binding to biomolecules, modified with azide or nitrone groups via copper-catalyzed 1,3-dipolar cycloaddition reactions [22,23,24]. These alkynyl derivatives may find even broader applications because alkynes are used in the synthesis of numerous heterocyclic systems; for recent reviews, see [25,26,27].

## 2. Results and Discussion

### 2.1. Nitrones

**Scheme 1:**
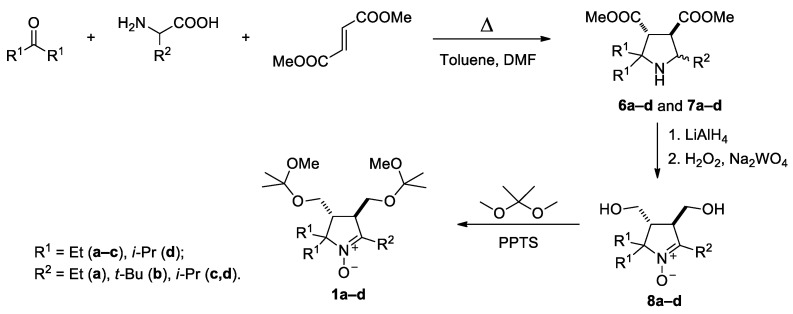
Synthesis of nitrones **1a**–**d**.

A series of sterically hindered nitrones **1**–**5** have been prepared to investigate their reactions with alkynylmagnesium halides. We have previously reported on the synthesis of diastereomeric pyrrolidines **6a**,**b** and **7a**,**b** and corresponding nitrones **8a**,**b** from amino acids, ketones and dimethyl fumarate according to Scheme 1 [17,19]. The pyrrolidines **6c**,**d** and **7c**,**d** were prepared in analogy to the published procedures. The reaction afforded the mixtures of diastereomers in a 1:1 ratio (cf. [17,19]), and the individual diastereomers were isolated using column chromatography. The mixtures were subjected to reduction of the ester groups with LiAlH_4_ and oxidation with tungstate–hydrogen peroxide system to give the nitrones **8c**,**d** (racemates). We noticed earlier that treatment of 3,4-bis-(hydroxymethyl)-1-pyrroline1-oxideswith Grignard reagents mightlead to THF-insoluble precipitate formation, presumably, magnesium alcoholates [17]. To avoid extra consumption of Grignard reagent and to prevent precipitation of magnesium alcoholates from the reaction mixtures, the hydroxy groups in the nitrones **8a**–**d** were protected via treatment with 2,2-dimethoxypropane, and the resulting nitrones **1a**–**d** were used for nitroxides syntheses.

**Scheme 2:**
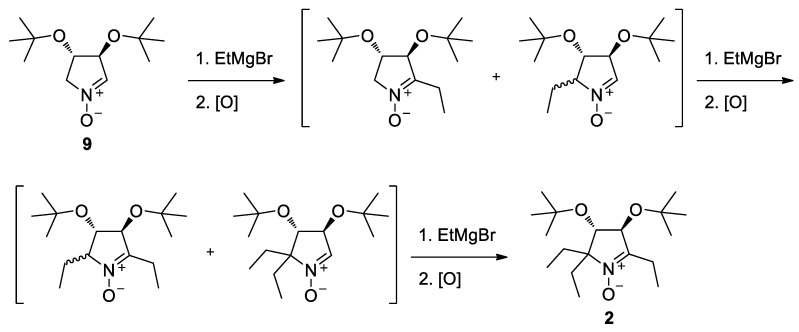
Synthesis of nitrone **2**.

The nitrone **2** was prepared from **9** [28,29] by successive introduction of three ethyl groups via reaction with ethylmagnesium bromide and oxidation (Scheme 2). The isomers formed in the intermediate steps were not separated, and the crude mixture was used in the second and third steps affording chiral nitrone **2** as a final product with a 25% yield.

**Scheme 3:**
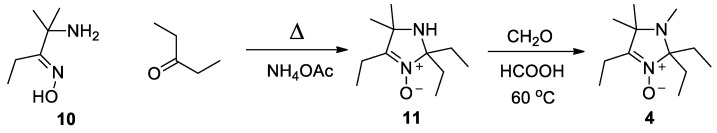
Synthesis of nitrone **4**.

To prepare nitrone **4**, the 2-amino-2-methylpentan-3-one oxime (**10**) was heated under reflux with pentane-3-one in methanol using ammonium acetate as a catalyst (cf. [30]), Scheme 3. The resulting nitrone **11** was subjected to Eschweiler-Clarke alkylation in analogy to literature protocol [31].

The nitrones **3** [20] and **5** [19] were prepared according to literature protocols.

### 2.2. Nitroxides

**Scheme 4:**
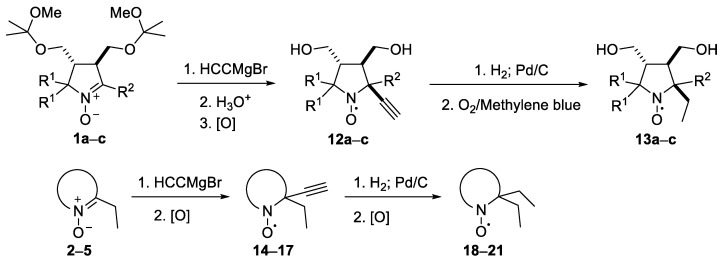
Synthesis of nitroxides.

Nitrones **1**–**5** were treated with a 10-fold excess of ethynylmagnesium bromide in THF, then quenched with water and oxidized (Scheme 4). The reaction products, times and yields of corresponding nitroxides are given in Table 1. In the case of **1**, processing of the reaction mixtures implied treatment with an aqueous acid solution to remove the protecting groups. Noteworthily, the conversion of **1b** and **c** was incomplete under these conditions, and TLC analysis of the quenched reaction mixtures showed the presence of the starting compounds. Bulky substituents at nitrone group carbon atom of **1** strongly decreased the reaction rate but didnot influence much the yield of the nitroxide. More hindered nitrone **1d** was quantitatively recovered from the reaction mixture after stirring with ethynylmagnesium bromide for a week.

The addition of ethynylmagnesium bromide to **1a**–**c** proceeded with higher selectivity than that of vinylmagnesium bromide affording only one diastereomer (cf. [19]). The structures **12a**–**c** were assigned based on X-ray analysis data (see Figures S2–S4 in the Supplementary Information in this article “X-ray diffraction data”). Selective formation of **12a**–**c** presumably results from the coordination of the organometallic reagent with the oxygen atom of the neighboring alkoxymethyl group. Interestingly, the reaction with chiral nitrone **2** also gave a single isomer, a chiral nitroxide **14**, but the ethynyl group entered from the opposite side, *trans-* to the neighboring *tert*-butoxy group (Figure S5 in the Supplementary Information in this article“X-ray diffraction data”).

**Scheme 5:**
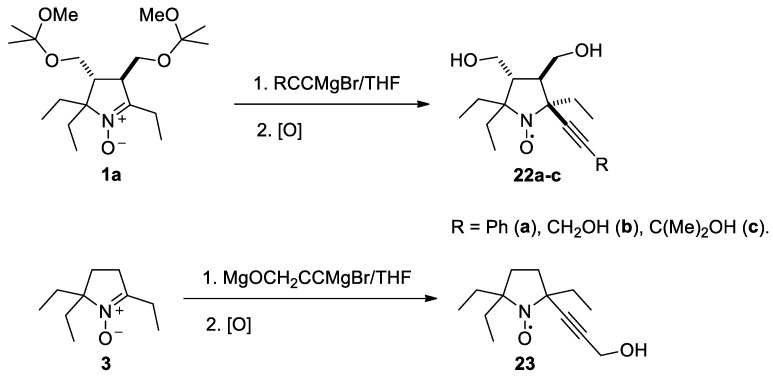
Preparation of nitroxides **22a**–**c** and **23**.

To introduce larger alkynyl groups corresponding organometallic reagents were prepared from terminal acetylenes and EtMgBr. The reaction with **1a** and **3** afforded nitroxides **22a**–**c** and **23** with 54–66% yield (Scheme 5). The stereochemistry of this reaction was similar to that of ethynylmagnesium bromide addition, see the X-ray analysis data in Figures S6–S8 in the Supplementary Information in this article“X-ray diffraction data.”

We have shown earlier that careful hydrogenation of ethynyl-substituted nitroxide in THF under atmospheric pressure allows for avoiding undesired over-reduction of the nitroxide group to an amine. The resulting ethyl-substituted hydroxylamine can then be easily oxidized to the corresponding nitroxide [8]. Using this procedure, nitroxides **12a**–**c**, **14**–**17** were converted to nitroxides **13a**–**c**, **18**–**21** (Scheme 4 and Table 1).

It should be noted that, in agreement with our previous observations [17,19], nitroxides **13a**–**c**, **18**–**20** can’t be prepared via the direct addition of EtMgBr. The overall yield of **13a** from **8a** using the procedures described here is nearly the same as in the previously described procedure with vinylmagnesium bromide [19]. The reaction of **5** with EtMgBr gives a lower yield of **21** (45%) [15] than the above two-step procedure with ethynylmagnesium bromide (Table 1).

### 2.3. EPR Spectra

**Figure 2:**
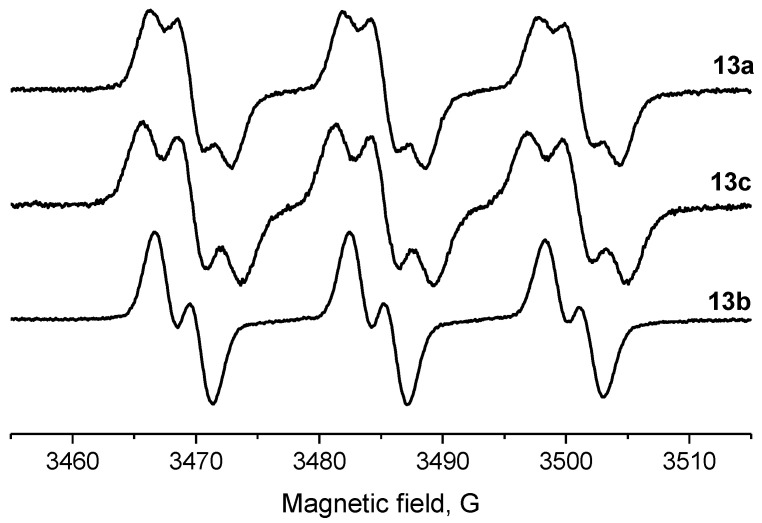
EPR spectra of nitroxides **13a** (**top**), **13c** (**middle**) and **13b** (**bottom**; for conditions, see the Section 3).

Parameters of the EPR spectra of the new nitroxides are given in Table 2. We have shown earlier that in nitroxides with a 3,4-disubstituted five-membered saturated ring, each pair of geminal ethyl groups at twoand fivepositions of the heterocycle produces one large (ca. 2 G) additional splitting due to hfi with one of the methylene hydrogens of the ethyl group [8,19,32]. Replacement of one of the geminal ethyls with another group may change the hyperfine structure of the spectrum greatly. The data on **13a**–**c** in Table 2 and Figure 2 demonstrate the remarkable evolution of the EPR spectra upon the increase of the steric demand of the substituent. The spectrum of **13c** follows the pattern described for **13a** [19], with two additional large splittings on γ-hydrogens. Replacement of one of the ethyl groups with theisopropyl one resulted in an increase of both hfi constants (by ca. 50% and 12%). Interestingly, the *tert*-butyl group (in **13b**) produces an opposite effect showing only one doublet splitting on one of the γ-hydrogens (cf. [17]).

**Figure 3:**
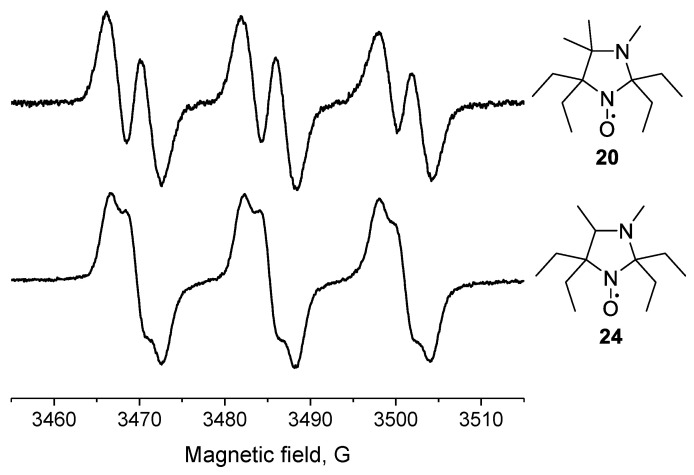
EPR spectra of nitroxides **20** and **24**.

Another example of an unusual hyperfine structure in the EPR spectrum is demonstrated by **20**. The EPR spectrum of close analog of this nitroxide, 2,2,5,5-tetraethyl-3,4-dimethylimidazolidine-1-oxyl (**24**; Figure 3), is known to contain two splitting constants (ca. 2 G) [32], while **20** showed a single additional splitting with *a_H_* = 3.64 G.

The above examples demonstrate that minor changes in the stricture of 2,2,5,5-tetraethyl-substituted five-membered ring nitroxides may lead to drastic changes in their EPR spectra. To the best of our knowledge, similar effects never occur for 2,2,5,5-tetramethyl nitroxides.

## 3. Materials and Methods

### 3.1. General Information

The 2-amino-2-methylpentan-3-one oxime (**10**) was obtained from the Pilot Plant of N. N. Vorozhtsov Novosibirsk Institute of Organic Chemistry SB RAS, chemprod@nioch.nsc.ru. The compounds **3** [20] and **5** [15], **8a** [19], **8b** [17], and **9** [29] were prepared according to the literature. Ethynyl magnesium bromide solutions (0.5–1 M) were prepared according to the protocol described in [33].

The IR spectra were recorded on a Bruker Vector 22 FT-IR spectrometer (Bruker, Billerica, MA, USA) in KBr pellets (1:150 ratio) or in neat samples (see the Supplementary Information in this article pp. 27–37) and are reported in wave numbers (cm^−1^). ^1^H NMR spectra were recorded on a Bruker AV 300 (300.132 MHz), AV 400(400.134 MHz) and DRX 500 (500.130 MHz) spectrometers (Bruker, Billerica, MA, USA). 13C NMR spectra were recorded on a Bruker AV 300 (75.467 MHz), AV 400 (100.614 MHz) and DRX 500 (125.758 MHz) spectrometers (see the Supplementary Information in this article pp. 5–26). All the NMR spectra were acquired for 5–10% solutions in CDCl_3_, (CD_3_)_2_SO or CDCl_3_-CD_3_OD mixtures at 300 K using the signal of the solvent as a standard. NMR spectra of nitroxides for analysis and structure assignment were recorded after reduction with Zn in CD_3_OD-CF_3_COOH at 65 °C as described in [8] or with Zn and ND_4_Cl in CD_3_OD at 5 °C. HRMS analyses were performedusinga High-Resolution Mass Spectrometer DFS (Thermo Electron, Waltham, MA, USA).

HPLC analyses were carried out using an HPLC-UV (Agilent 1100, Agilent Technologies Inc., Santa Clara, CA, USA) with a Zorbax C8 column (250 mm × 4.6 mm with 5 µm particle size; Agilent Technologies Inc., USA). The column was thermostatically controlled at 35 °C. Samples were dissolved in methanol (2 mg/mL) and 3 µL of the solutionwas injected. The mobile phase was composed of A (0.1% H_3_PO_4_ in water) and B (methanol) with the following gradient elution: 0–7 min 80% B, 7–10 min 100% B, 10–12 min 100% B, the flow rate was set to 1.0 mL/min, and peaks were detected using a wavelength of 230 nm.

Reactions were monitored by TLC on precoated TLC sheets ALUGRAM Xtra SIL G/UV_254_ ((Macherey-Nagel GmbH & Co. KG, Düren, Germany) using UV light 254 nm, 1% aqueous permanganate, 10% solution of phosphomolybdic acid in ethanol and Dragendorff reagent as visualizing agents. Kieselgel60 (Macherey-Nagel GmbH & Co. KG) was utilized as an adsorbent for column chromatography.

EPR experiments were performed on X-band (9.8 GHz) EPR spectrometer ER-200D (Bruker). All measurements were performed in 50 μL glass capillary. The radicals were dissolved in oxygen-free distilled water at a concentration of 0.2 mM. EPR settings: modulation amplitude 0.5 G, MW, power 5 mW time constant50 ms; total acquisition time 3 min. The water-insoluble radicals were dissolved in a water/ethanol mixture (50%/50%) at a concentration of 0.1 mM. Data simulation was performed with the free software Winsim.

CCDC 2,209,668 (**8d**), 2,209,669 (**12a**), 2,209,670 (**12c**), 220,9671 (**12b**), 2,209,672 (**13b**), 2,209,673 (**22a**), 2,209,674 (**22b**), 2,209,675 (**22c**), 2,209,676 (**14**) contain the supplementary crystallographic data for this paper. These data can be obtained free of charge from The Cambridge Crystallographic Data Centre https://www.ccdc.cam.ac.uk/structures/, accessed on 27 September 2022.

### 3.2. Synthesis

#### 3.2.1. General Method of Synthesis of 3,4-Bis(methoxycarbonyl)pyrrolidines **6c**,**d** and **7c**,**d**

A mixture of valine (11.7 g, 0.1 mol), dimethyl fumarate (14.4 g, 0.1 mol), ketone (1 mol), DMF (100 mL) and toluene (100 mL)was placed into Dean-Stark apparatus and stirred under reflux in a week (TLC control on silica gel, hexane–ethylacetate 4:1, visualizationwith Dragendorff’s reagent). The solvent was distilled off in a vacuum, the residue was dissolved in ethyl acetate, and the solution was extracted with 5% aqueous sulfuric acid. Acidic extracts were basified with Na_2_CO_3_ and extracted with ethyl acetate. The extract was dried with Na_2_CO_3,_ and the solvent was distilled off in a vacuum to give a yellow oil, a mixture of isomers, which was used for the next step without further purification. For the analysis, the isomers were separated using column chromatography on silica gel (hexane/ethyl acetate 4/1).

*2,2-Diethyl-5-isopropyl-3,4-bis(methoxycarbonyl)pyrrolidines*. **6c**, yield 20%, colorless oil, IR (neat) *ν*_max_: 1726 (C=O). ^1^H NMR (400 MHz; CDCl_3_,δ):0.79 (t, J_t_ = 7.4 Hz, 3H), 0.91 (d, J_d_ = 6.6 Hz, 3H), 0.94 (t, J_t_ = 7.4 Hz, 3H), 0.95 (d, J_d_ = 6.6 Hz, 3H), 1.12 (dq, J_d_ = 13.9 Hz, J_q_ = 7.2 Hz, 1H), 1.44 (dq, J_d_ = 13.9 Hz, J_q_ = 7.5 Hz, 1H), 1.51 (dsp, J_d_ = 9.2 Hz, J_sp_ = 6.6 Hz, 1H), 1.54–1.60 (br, 1H), 1.65 (dq, J_d_ = 15.1 Hz, J_q_ = 7.4 Hz, 1H), 1.68 (dq, J_d_ = 15.1 Hz, J_q_ = 7.4 Hz, 1H), 2.94 (dd, J_d1_ = 9.2 Hz, J_d2_ = 7.0 Hz, 1H), 3.09 (d, J_d_ = 4.5 Hz, 1H), 3.45 (dd, J_d1_ = 7.0 Hz, J_d2_ = 4.5 Hz, 1H), 3.63 (s, 3H), 3.63 (s, 3H). ^13^C{^1^H} NMR (100 MHz, CDCl_3_, δ): 7.9, 8.0, 20.7, 20.9, 25.4, 29.4, 29.7, 51.0, 51.3, 51.5, 56.7, 67.3, 68.0, 173.2, 175.2. Anal. calcd for C_15_H_27_NO_4_: C, 63.13; H, 9.24; N, 4.91; found: C, 63.14; H, 9.54; N, 4.90. **7c**, yield 20%, colorless oil, IR (neat) *ν*_max_: 1735 (C=O). ^1^H NMR (400 MHz; CDCl_3_, δ): 0.79 (t, J_t_ = 7.4 Hz, 3H), 0.85 (t, J_t_ = 7.4 Hz, 3H), 0.87 (d, J_d_ = 6.6 Hz, 3H), 0.91 (d, J_d_ = 6.6 Hz, 3H), 1.28 (q, J_q_ = 7.4 Hz, 2H), 1.37–1.43 (br, 1H), 1.51 (dq, J_d_ = 13.9 Hz, J_q_ = 7.2 Hz, 1H), 1.57 (dq, J_d_ = 13.9 Hz, J_q_ = 7.4 Hz, 1H), 1.71 (dsp, J_d_ = 5.7 Hz, J_sp_ = 6.6 Hz, 1H), 3.03 (dd, J_d1_ = 8.8 Hz, J_d2_ = 7.7 Hz, 1H), 3.08 (d, J_d_ = 7.7 Hz, 1H), 3.10 (dd, J_d1_ = 8.8 Hz, J_d2_ = 5.7 Hz, 1H), 3.62 (s, 3H), 3.62 (s, 3H). ^13^C{^1^H} NMR (100 MHz, CDCl_3_, δ):7.9, 8.0, 18.8, 19.5, 28.2, 29.1, 32.0, 51.4, 51.5, 51.8, 56.4, 66.8, 67.3, 173.4, 174.7. Anal. calcd for C_15_H_27_NO_4_: C, 63.13; H, 9.24; N, 4.91; found: C, 63.05; H, 9.37; N, 4.82.

*2,2,5-Triisopropyl-3,4-bis(methoxycarbonyl)pyrrolidines*. **6d**, yield 8%, colorless oil, IR (neat) *ν*_max_: 1733 (C=O). ^1^H NMR (300 MHz; CDCl_3_, δ): 0.83 (d, J_d_ = 7.0 Hz, 3H), 0.84 (d, J_d_ = 6.6 Hz, 3H), 0.88 (d, J_d_ = 6.6 Hz, 3H), 0.92 (d, J_d_ = 7.0 Hz, 3H), 0.97 (d, J_d_ = 6.8 Hz, 3H), 1.05 (d, J_d_ = 6.8 Hz, 3H), 1.50–1.59 (br, 1H), 1.67 (dsp, J_d_ = 7.5 Hz, J_sp_ = 6.6 Hz, 1H), 2.00 (sp, J_sp_ = 7.0 Hz, 1H), 2.16 (sp, J_sp_ = 6.8 Hz, 1H), 3.13 (dd, J_d1_ = 9.0 Hz, J_d2_ = 7.5 Hz, 1H), 3.32 (d, J_d_ = 8.9 Hz, 1H), 3.59 (dd, J_d1_ = 9.0 Hz, J_d2_ = 8.9 Hz), 3.63 (s, 6H). ^13^C{^1^H} NMR (75 MHz, CDCl_3_, δ): 17.9, 18.3, 18.9, 19.3, 19.5, 20.8, 29.9, 31.4, 31.6, 49.9, 51.4, 51.5, 53.3, 64.5, 71.1, 173.2, 174.7. Anal. calcd for C_17_H_31_NO_4_: C, 65.14; H, 9.97; N, 4.47; found: C, 65.37; H, 9.96; N, 4.50. **7d**, yield 8%, colorless oil, IR (neat) *ν*_max_: 1735 (C=O). ^1^H NMR (300 MHz; CDCl_3_, δ): 0.75 (d, J_d_ = 6.6 Hz, 3H), 0.78 (d, J_d_ = 6.8 Hz, 3H), 0.80 (d, J_d_ = 6.9 Hz, 3H); 0.92 (d, J_d_ = 6.7 Hz, 3H), 0.96 (d, J_d_ = 6.9 Hz, 3H); 1.02 (d, J_d_ = 6.7 Hz, 3H), 1.14–1.21 (br, 1H), 1.71 (dsep, J_d_ = 4.8 Hz, J_sep_ = 6.9 Hz, 1H), 2.01 (sep, J_sep_ = 6.9 Hz, 1H), 2.12 (sep, J_sp_ = 6.7 Hz, 1H), 3.02 (dd, J_d1_ = 10.5 Hz, J_d2_ = 10.5 Hz, 1H), 3.09 (dd, J_d1_ = 10.5 Hz, J_d2_ = 4.8 Hz, 1H), 3.26 (d, J_d_ = 10.5 Hz, 1H), 3.62 (s, 3H), 3.66 (s, 3H). ^13^C{^1^H} NMR (75 MHz, CDCl_3_, δ): 17.2, 17.5, 18.1, 18.3, 18.5, 19.4, 31.1, 31.1, 36.8, 50.8, 51.1, 51.7, 51.7, 66.5, 69.30, 172.9, 174.8. Anal. calcd for C_17_H_31_NO_4_: C, 65.14; H, 9.97; N, 4.47; found: C, 64.91; H, 9.83; N, 4.63.

#### 3.2.2. General Method of Synthesis of 3,4-Bis(hydroxymethyl)-3,4-dihydro-2H-pyrrole 1-oxides (**8c**,**d**)

A solution of crude amines (mixture of isomers **6c**,**d** or **7c**,**d**; 0.01 mol) in dry THF (10 mL) was added dropwise to a stirred solution of LiAlH_4_ (0.76 g, 0.02 mol) in dry THF (50 mL). The mixture was stirred at ambient temperature for 1 h, then the flask was placed into a cold water bath and quenched with water. The organic phase was separated via decantation, the remaining wet precipitate was washed with THF 3 × 20 mL, and the combined extracts were evaporated in a vacuum. The residue was dissolved in methanol (50 mL) and mixed with a solution of sodium tungstate dihydrate (0.33 g, 0.001 mol) and EDTA disodium salt (0.34 g, 0.001 mol) in water (25 mL) and hydrogen peroxide 30% (5 mL, 0.05 mol) was added. The solution was allowed to stand at ambient temperature for a few days (TLC control on silica gel, ethylacetate–methanol9:1, visualization with UV-254 and Dragendorff’s reagent). Then the catalytic amount of MnO_2_ (0.1 g, 1.1 mmol) was carefully added to quench the remaining H_2_O_2_. After oxygen evolution ceased, the solution was evaporated in a vacuum. The residue was triturated with a chloroform/ethanol 100/1 mixture, the catalyst was filtered off, and the solvent was distilled off in a vacuum. The residue was triturated with diethyl ether, and a yellowish crystalline precipitate was collected, which was used for the next step without further purification. For the analysis, the nitrone was purified using column chromatography on silica gel (ethyl acetate).

*2,2-Diethyl-5-isopropyl-3,4-bis(hydroxymethyl)-3,4-dihydro-2H-pyrrole 1-oxide* (**8c**). Yield 1.9 g (80%), colorless crystals, m.p. 87–90 °C (from ethyl acetate–diethyl ether). IR (KBr) ν_max_: 1592 (C=N). ^1^H NMR (400 MHz; CDCl_3_, δ):0.71 (t, J_t_ = 7.4 Hz, 3H), 0.84 (t, J_t_ = 7.4 Hz, 3H), 1.12 (d, J_d_ = 7.0 Hz, 3H), 1.17 (d, J_d_ = 7.0 Hz, 3H), 1.47 (dq, J_d_ = 14.4 Hz, J_q_ = 7.4 Hz, 1H), 1.59 (dq, J_d_ = 14.3 Hz, J_q_ = 7.4 Hz, 1H), 1.71 (dq, J_d_ = 14.3 Hz, J_q_ = 7.4 Hz, 1H), 1.82 (dq, J_d_ = 14.3 Hz, J_q_ = 7.4 Hz, 1H), 2.30 (ddd, J_d1_ = 8.9 Hz, J_d2_ = 8.6 Hz, J_d3_ = 4.4 Hz, 1H), 2.86 (ddd, J_d1_ = 8.9 Hz, J_d2_ = 7.6 Hz, J_d3_ = 3.2 Hz, 1H), 3.22 (sp, J_sp_ = 7.0 Hz, 1H), 3.35 (dd, J_d1_ = 10.0 Hz, J_d2_ = 8.6 Hz, 1H), 3.65 (dd, J_d1_ = 10.9 Hz, J_d2_ = 7.6 Hz, 1H), 3.69–3.76 (br, 1H), 4.01–4.09 (br, 1H), 5.23–5.34 (br, 1H), 5.38–5.47 (br, 1H). ^13^C{^1^H} NMR (100 MHz, CDCl_3_, δ): 7.4, 9.2, 17.4, 18.0, 26.2, 27.5, 30.5, 45.3, 50.4, 61.1, 63.5, 80.1, 153.4. Anal. calcd for C_13_H_25_NO_3_: C, 64.16; H, 10.36; N, 5.76; found: C, 64.14; H, 10.90 N, 5.50.

*2,2,5-Triisopropyl-3,4-bis(hydroxymethyl)-3,4-dihydro-2H-pyrrole 1-oxide* (**8d**). Yield 1.1 g (42%), colorless crystals, m.p. 160–161 °C (from ethyl acetate–diethyl ether). IR (KBr) ν_max_: 1595 (C=N). ^1^H NMR (500 MHz; CDCl_3_,δ):0.77 (d, J_d_ = 6.6 Hz, 3H), 0.79 (d, J_d_ = 6.6 Hz, 3H), 0.87 (d, J_d_ = 7.0, 3H), 1.07 (d, J_d_ = 7.0 Hz, 3H), 1.14 (d, J_d_ = 7.0 Hz, 3H), 1.16 (d, J_d_ = 7.0 Hz, 3H), 1.78 (sp, J_sp_ = 6.6 Hz, 1H), 2.20 (ddd, J_d1_ = 10.5 Hz, J_d2_ = 6.9 Hz, J_d3_ = 3.7 Hz, 1H), 2.64 (sp, J_sp_ = 7.0 Hz, 1H), 2.83 (ddd, J_d1_ = 9.4 Hz, J_d2_ = 6.9 Hz, J_d3_ = 3.3 Hz, 1H), 3.18 (sp, J_sp_ = 7.0 Hz, 1H), 3.26 (dd, J_d1_ = 10.3 Hz, J_d2_ = 9.4 Hz, 1H), 3.57 (dd, J_d1_ = 10.5 Hz, J_d2_ = 10.0 Hz, 1H), 3.69 (dd, J_d1_ = 10.0 Hz, J_d2_ = 3.7 Hz, 1H), 4.01 (dd, J_d1_ = 10.3 Hz, J_d2_ = 3.3 Hz, 1H), 5.22–5.26 (br, 1H), 5.35–5.40 (br, 1H). ^13^C{^1^H} NMR (125 MHz, CDCl_3_, δ): 15.4, 16.5, 17.0, 17.6, 17.8, 18.9, 25.9, 29.3, 30.1, 42.8, 51.1, 62.3 63.4, 83.5, 151.2. Anal. calcdfor C_15_H_29_NO_3_: C, 66.38; H, 10.77; N, 5.16; found: C, 66.45; H, 10.71; N, 5.14.

#### 3.2.3. General Method of Protecting Hydroxyl Groups with 2,2-Dimethoxypropane

2,2-Dimethoxypropane (18 mL, 0.15 mol) was added dropwise to a mixture of nitrone (**8a**–**d**) (5 mmol), pyridinium p-toluenesulfonate (0.1 g, 0.4 mmol), molecular saves 3A and dry chloroform (50 mL). The mixture was stirred at ambient temperature for 1 day (TLC control on silica gel, ethyl acetate–methanol9:1, visualization with UV-254 and Dragendorff’s reagent). Then the molecular saves 3A were filtered off. The organic phase was washed with a 10% solution of sodium carbonate in water (50 mL) and was dried with Na_2_CO_3,_ and filtered off. The solvent was evaporated in a vacuum, and the residue was crystallized from hexane.

*2,2,5-Triethyl-3,4-bis(((2-methoxypropan-2-yl)oxy)methyl)-3,4-dihydro-2H-pyrrole 1-oxide* (**1a**). Yield 1.2 g (63%), colorless crystals, m.p. 41–42 °C. IR (KBr) ν_max_: 2829 (OC-H), 1600 (C=N), 1153 (C-O-C). ^1^H NMR (400 MHz; CDCl_3_,δ): 0.71 (t, J_t_ = 7.3 Hz, 3H), 0.79 (t, J_t_ = 7.4 Hz, 3H), 1.03 (t, J_t_ = 7.4 Hz, 3H), 1.21 (s, 3H), 1.22 (s, 3H), 1.24 (s, 6H), 1.52 (dq, J_d_ = 14.6 Hz, J_q_ = 7.3 Hz, 1H), 1.57 (dq, J_d_ = 14.4 Hz, J_q_ = 7.4 Hz, 1H), 1.73 (dq, J_d_ = 14.6 Hz, J_q_ = 7.3 Hz, 1H), 1.87 (dq, J_d_ = 14.4 Hz, J_q_ = 7.4 Hz, 1H), 2.14 (dq, J_d_ = 14.0 Hz, J_q_ = 7.4 Hz, 1H), 2.45 (ddd, J_d1_ = 8.8 Hz, J_d2_ = 7.6 Hz, J_d3_ = 7.1 Hz, 1H), 2.61 (ddd, J_d1_ = 8.8 Hz, J_d2_ = 4.3 Hz, J_d3_ = 3.8 Hz, 1H), 2.73 (dq, J_d_ = 14.0 Hz, J_q_ = 7.4 Hz, 1H), 3.09 (s, 3H), 3.10 (s, 3H), 3.46 (dd, J_d1_ = 7.1 Hz, J_d2_ = 9.0 Hz, 1H), 3.47 (dd, J_d1_ = 4.3 Hz, J_d2_ = 9.4 Hz, 1H), 3.49 (dd, J_d1_ = 7.6 Hz, J_d2_ = 9.0 Hz, 1H), 3.51 (dd, j_d1_ = 9.4 Hz, J_d2_ = 3.8 Hz, 1H). ^13^C{^1^H} NMR (100 MHz, CDCl_3_, δ): 7.6, 9.1, 9.3, 18.2, 27.4, 30.5, 24.0, 24.1, 24.1, 24.1, 38.7, 45.6, 48.4, 48.5, 59.8, 60.1, 79.6, 99.8, 99.8, 148.5. Anal. calcd for C_20_H_39_NO_5_: C, 64.31; H, 10.52; N, 3.75; found: C, 64.09; H, 10.48; N, 3.66.

*5-tert-Butyl-2,2-diethyl-3,4-bis(((2-methoxypropan-2-yl)oxy)methyl)-3,4-dihydro-2H-pyrrole 1-oxide* (**1b**). Yield 1.2 g (78%), colorless crystals, m.p. 55–57 °C. IR (KBr) ν_max_: 2829 (OC-H), 1554 (C=N), 1152 (C-O-C). ^1^H NMR (500 MHz; CDCl_3_, δ): 0.72 (t, J_t_ = 7.4 Hz, 3H), 0.83 (t, J_t_ = 7.4 Hz, 3H), 1.25 (s, 3H), 1.25 (s, 3H), 1.25 (s, 3H), 1.26 (s, 3H), 1.30 (s, 9H), 1.58 (dq, J_d_ = 14.7 Hz, J_q_ = 7.1 Hz, 1H), 1.60 (dq, J_d_ = 13.6 Hz, J_q_ = 7.4 Hz, 1H), 1.71 (dq, J_d_ = 14.7 Hz, J_q_ = 7.4 Hz, 1H), 1.85 (dq, J_d_ = 13.6 Hz, J_q_ = 7.4 Hz, 1H), 2.43 (q, J_q_ = 7.3 Hz, 1H), 2.66 (dt, J_d_ = 7.3 Hz, J_t_ = 3.3 Hz, 1H), 3.11 (s, 3H), 3.16 (s, 3H), 3.47 (d, J_d_ = 7.3 Hz, 2H), 3.60 (d, J_d_ = 3.3 Hz, 2H). ^13^C{^1^H} NMR (125 MHz, CDCl_3_, δ): 7.6, 9.2, 23.9, 24.0, 24.1, 24.2, 25.7, 31.5, 34.5, 40.2, 46.8, 48.3, 48.6, 60.4, 61.8, 79.7, 99.7, 99.9, 150.4. Anal. calcd for C_22_H_43_NO_5_: C, 65.80; H, 10.79; N, 3.49; found: C, 65.86; H, 10.53; N, 3.66.

*2,2-Diethyl-5-isopropyl-3,4-bis(((2-methoxypropan-2-yl)oxy)methyl)-3,4-dihydro-2H-pyrrole 1-oxide* (**1c**). Yield 1.5 g (76%) of colorless crystals, m.p. 31–33 °C (dec.). IR (KBr) ν_max_: 2829 (OC-H), 1589 (C=N), 1153 (C-O-C). ^1^H NMR (500 MHz; CDCl_3_,δ):0.73 (t, J_t_ = 7.3 Hz, 3H), 0.82 (t, J_t_ = 7.5 Hz, 3H), 1.17 (d, J_d_ = 7.1 Hz, 3H), 1.19 (d, J_d_ = 7.1 Hz, 3H), 1.25 (s, 3H), 1.26 (s, 3H), 1.27 (s, 6H), 1.58 (dq, J_d_ = 14.3 Hz, J_q_ = 7.4 Hz, 2H), 1.75 (dq, J_d_ = 14.6 Hz, J_q_ = 7.5 Hz, 1H), 1.89 (dq, J_d_ = 14.0 Hz, J_q_ = 7.3 Hz, 1H), 2.46 (ddd, J_d1_ = 8.5 Hz, J_d2_ = 7.4 Hz, J_d3_ = 7.4 Hz, 1H), 2.62 (ddd, j_d1_ = 8.5 Hz, J_d2_ = 4.5 Hz, J_d3_ = 3.7 Hz, 1H), 3.09 (sp, J_sp_ = 7.1 Hz, 1H), 3.12 (s, 3H), 3.15 (s, 2H), 3.48 (dd, J_d1_ = 9.6 Hz, J_d2_ = 7.4 Hz, 1H), 3.49 (dd, J_d1_ = 9.2 Hz, J_d2_ = 4.5 Hz, 1H), 3.54 (dd, J_d1_ = 9.2 Hz, J_d2_ = 7.4 Hz, 1H), 3.58 (dd, J_d1_ = 9.6 Hz, J_d2_ = 3.7 Hz, 1H). ^13^C{^1^H} NMR (125 MHz, CDCl_3_, δ): 7.5, 9.2, 17.3, 17.4, 23.9,24.0,24.1,24.1, 26.2, 27.5, 30.6, 39.4, 45.9, 48.3, 48.5, 60.2, 60.5, 79.5, 99.8, 99.9, 150.1. Anal. calcd for C_21_H_41_NO_5_: C, 65.08; H, 10.66; N, 3.61.; found: C, 64.95; H, 10.75; N, 3.66.

*2,2,5-Triisopropyl-3,4-bis(((2-methoxypropan-2-yl)oxy)methyl)-3,4-dihydro-2H-pyrrole 1-oxide* (**1d**). Yield 1.2 g (58%), colorless crystals, m.p. 59–63 °C. IR (KBr) ν_max_: 2829 (OC-H), 1594 (C=N), 1153 (C-O-C). ^1^H NMR (400 MHz; CDCl_3_,δ): 0.82 (d, J_d_ = 6.6 Hz, 3H), 0.83 (d, J_d_ = 6.6 Hz, 3H), 0.86 (d, J_d_ = 7.1 Hz, 3H), 1.17 (d, J_d_ = 7.1 Hz, 3H), 1.19 (d, J_d_ = 7.1 Hz, 3H), 1.20 (d, J_d_ = 6.6 Hz, 3H), 1.28 (s, 3H), 1.28 (s, 9H), 1.86 (sp, J_sp_ = 6.6 Hz, 1H), 2.43 (ddd, J_d1_ = 9.0 Hz, J_d2_ = 7.8 Hz, J_d3_ = 5.7 Hz, 1H), 2.63 (ddd, J_d1_ = 7.8 Hz, J_d2_ = 3.8 Hz, J_d3_ = 3.7 Hz, 1H), 2.70 (sp, J_d_ = 6.8 Hz, 1H), 3.07 (sp, J_sp_ = 7.1 Hz, 1H), 3.14 (s, 3H), 3.19 (s, 3H), 3.43 (dd, J_d1_ = 9.1 Hz, J_d2_ = 9.0 Hz, 1H), 3.49 (dd, J_d1_ = 9.1 Hz, J_d2_ = 5.7 Hz, 1H), 3.57 (dd, J_d1_ = 9.5 Hz, J_d2_ = 3.8 Hz, 1H), 3.65 (dd, J_d1_ = 9.5 Hz, J_d2_ = 3.7 Hz, 1H). ^13^C{^1^H} NMR (100 MHz, CDCl_3_, δ): 16.1, 16.9, 17.0, 17.1, 18.1, 19.2, 24.0, 24.1, 24.2, 26.1, 36.3, 28.0, 48.4, 48.6, 60.7, 61.8, 82.2, 99.8, 99.9, 149.5. Anal. calcd for C_23_H_45_NO_5_: C, 66.47; H, 10.91; N, 3.37; found: C, 66.31; H, 10.87; N, 3.58.

*(3S,4S)-3,4-Di-tert-butoxy-2,2,5-triethyl-3,4-dihydro-2H-pyrrole 1-oxide* (**2**). A solution of ethyl magnesium bromide was prepared from ethyl bromide (1.4 g, 12.87 mmol) and Mg chips (0.36 g, 14.85 mmol) in dry Et_2_O. A solution of (3S,4S)-3,4-di-*tert*-butoxy-3,4-dihydro-2*H*-pyrrole 1-oxide (2.27 g, 9.9 mmol) in dry Et_2_O (15 mL) was added dropwise. The reaction mixture was stirred for 2 h, quenched with water (0.21 mL, 11.85 mmol), the organic phase was separated, and dry air was bubbled into the solution for 5 h (TLC control, silica gel, eluent EtOAc). The resulting solution was treated with another portion of ethylmagnesium bromide and processed as described above. This procedure was repeated once again, the organic layer was concentrated in a vacuum, and the residue was purified using column chromatography (silica gel, EtOAc) to give **2** as a colorless waxy solid. Yield 0.76 g (25%). IR (KBr) ν_max_: 2973 (C-H). UV (EtOH): 239 (3,94). [α]^26^_D_ = + 12.7 (c 0.85, CHCl_3_). ^1^H NMR (500 MHz, CDCl_3_, δ): 0.77 (t, J_t_ = 7.4 Hz, 3H), 0.78 (t, J_t_ = 7.4 Hz, 3H), 1.07 (t, J_t_ = 7.4 Hz, 3H), 1.17 (s, 9H), 1.21 (s, 9H), 1.43 (dq, J_d_ = 14.7 Hz, J_q_ = 7.4 Hz, 1H), 1.64 (dq, J_d_ = 14.0 Hz, J_q_ = 7.4 Hz, 1H), 1.67 (dq, J_d_ = 14.0 Hz, J_q_ = 7.4 Hz, 1H), 2.17 (dq, J_d_ = 14.7 Hz, J_q_ = 7.4 Hz, 1H), 2.37 (dq, J_d_ = 13.5 Hz, J_q_ = 7.4 Hz, 1H), 2.50 (dq, J_d_ = 13.5 Hz, J_q_ = 7.4 Hz, 1H), 3.94 (d, J_d_ = 5.7 Hz, 1H), 4.40 (d, J_d_ = 5.7 Hz, 1H). ^13^C{^1^H} NMR (125 MHz, CDCl_3_, δ): 7.5, 8.7, 9.0, 17.6, 26.8, 27.8, 29.0, 29.1, 74.4, 74.7, 76.7, 77.8, 78.9, 148.4. Anal. calcd for C_18_H_35_NO_3_: C, 68.97; H, 11.25; N, 4.47; found: C, 69.11; H, 11.17; N, 4.55.

*2,2,4-Triethyl-5,5-dimethyl-2,5-dihydroimidazole 3-oxide* (**11**). A solution of 2-amino-2-methylpentan-3-one oxime (**10**) (6.8 g, 52 mmol), pentane-3-one (14 g, 162 mmol) and ammonium acetate (5 g, 64 mmol) in methanol (30 mL) was heated under reflux for 3.5 h. The solution was concentrated in a vacuum, diluted with brine (100 mL) and extracted with EtOAc. The extract was dried with Na_2_CO_3_ and evaporated in a vacuum, with a bath temperature of 50 °C. The viscous residue was left overnight at 20 °C and solidified. The resulting crude **11** was used in the next step without purification. Yield 10 g (97%), colorless crystals, m.p. 40–44 °C (from hexane). IR(KBr) ν_max_: 3275 (NH), 1606 (C=N). ^1^H NMR (500 MHz; CDCl_3_, δ): 0.82 (t, J_t_ = 7.4 Hz, 6H), 1.13 (t, J_t_ = 7.5 Hz, 3H), 1.28 (s, 6H), 1.67 (dq, J_d_ = 14.0 Hz, J_q_ = 7.4 Hz, 2H), 1.83 (dq, J_d_ = 14.0 Hz, J_q_ = 7.4 Hz, 2H), 2.32 (q, J_q_ = 7.5 Hz, 2H). ^13^C{^1^H} NMR (125 MHz, CDCl_3_, δ): 7.5, 9.2, 17.3, 28.3, 30.1, 61.4, 92.7, 149.2. Anal. calcd for C_11_H_22_N_2_O: C, 66.62; H, 11.18; N, 14.13; found: C, 66.41; H, 10.97; N, 13.98.

*2,2,4-Triethyl-1,5,5-trimethyl-2,5-dihydroimidazole 3-oxide* (**4**). A solution of **11** (9.5 g, 48 mmol) in 37% aqueous formaldehyde (20 mL, 270 mmol) and 90% formic acid (20 mL, 470 mmol) was stirred at 60 °C overnight. The mixture was diluted with water (200 mL) and extracted with chloroform. The extract was washed with a saturated aqueous solution of Na_2_CO_3_ and dried with Na_2_CO_3_. The solvent was distilled off, affording **12** as a colorless oil, 7.8 g (77%). The compound was used without further purification. IR (neat) ν_max_: 2806 (CH, NMe), 1603 (C=N). ^1^H NMR (500 MHz; CDCl_3_, δ): 0.71 (t, J_t_ = 7.4 Hz, 6H), 1.17 (t, J_t_ = 7.4 Hz, 3H), 1.23 (s, 6H), 1.50 (dq, J_d_ = 14.5 Hz, J_q_ = 7.4 Hz, 2H), 1.92 (dq, J_d_ = 14.5 Hz, J_q_ = 7.4 Hz, 2H), 2.32 (s, 3H), 2.36 (q, J_q_ = 7.5 Hz, 2H). ^13^C{^1^H} NMR (125.77 MHz, CDCl_3_, δ): 8.1, 9.2, 17.4, 24.4, 25.9, 27.9, 61.2, 94.2, 150.3. Anal. calcd for C_12_H_24_N_2_O: C, 67.88; H, 11.39; N, 13.19; found: C, 67.49; H, 11.17; N, 13.28.

#### 3.2.4. General Method of Reaction of Nitrones **12** with Ethynyl Magnesium Bromide

A 0.5–1 M solution of ethynylmagnesium bromide in THF (20–10 mL, 10 mmol) was added to nitrone (1 mmol) and kept at ambient temperature for 1–8 weeks (TLC control on silica gel, ethylacetate, visualization with UV-254 and Dragendorff’s reagent). Then the mixture was quenched with water, the organic phase was separated via decantation, and the remaining wet precipitate was washed with THF 5 × 10 mL. The THF was distilled off in a vacuum, and the residue was dissolved in methanol (20 mL). A 1 M solution of sodium hydroxide in water (6 mL) was added to the mixture. Then, the methylene blue (3 mg, 0.01 mmol) was added to the mixture, and the air was bubbled until the solution turned dark blue. Then the mixture was diluted with water (20 mL) and a 1M solution of sulphuric acid (4 mL). Methanol was evaporated in a vacuum. The mixture was extracted with ethyl acetate (3 × 10 mL). The organic phase was evaporated in a vacuum, and the residue was purified using column chromatography on silica gel (hexane–ethyl acetate 1:1).

*2,2,5-Triethyl-5-ethynyl-3,4-bis(hydroxymethyl)-pyrrolidine-1-oxyl* (**12a**). Yield 0.173 g (68%), yellow crystals, m.p. 107–113 °C (diethyl ether). IR (KBr) ν_max_: 3220 (≡C-H), 2102 (C≡C). ^1^H NMR (300 MHz; CD_3_OD, Zn/CF_3_COOH,δ): 0.85 (t, J_t_ = 7.4 Hz, 3H), 0.87 (t, J_t_ = 7.4 Hz, 3H), 1.07 (t, J_t_ = 7.4 Hz, 3H), 1.72 (q, J_q_ = 7.4 Hz, 2H), 2.21–2.35 (m, 4H), 2.53–2.61 (m, 2H), 2.90 (s, 1H), 3.57 (dd, J_d1_ = 10.9 Hz, J_d2_ = 5.3 Hz, 1H), 3.67 (dd, J_d1_ = 10.9 Hz, J_d2_ = 3.0 Hz, 1H), 3.68 (dd, J_d1_ = 11.4 Hz, J_d2_ = 5.9 Hz, 1H), 3.80 (dd, J_d1_ = 11.4 Hz, J_d2_ = 3.5 Hz,1H). Anal. calcd for C_14_H_24_NO_3_: C, 66.11; H, 9.51; N, 5.51; found: C, 66.18; H, 9.65; N, 5.50.HRMS (EI/DFS) *m*/*z* [M]^+^calcd for C_14_H_24_NO_3_: 254.1751; found: 254.1753.

*5-tert-Butyl-2,2-diethyl-5-ethynyl-3,4-bis(hydroxymethyl)-pyrrolidine-1-oxyl* (**12b**). Yield 0.189 g (67%), yellow crystals, m.p. 113–114 °C (diethyl ether). IR (KBr) ν_max_: 3305 (≡C-H). ^1^H NMR (300 MHz; CD_3_OD, Zn/CF_3_COOH, δ): 1.01 (t, J_t_ = 7.4 Hz, 3H), 1.05 (t, J_t_ = 7.4 Hz, 3H), 1.27 (s, 9H), 2.00 (q, J_q_ = 7.4, 2H), 2.08 (dq, J_d_ = 14.0 Hz, J_q_ = 7.4, 1H), 2.31 (dq, J_d_ = 14.0 Hz, J_q_ = 7.4 Hz, 1H), dt, J_d_ = 6.2 Hz, J_t_ = 4.7 Hz, 1H), 2.70 (ddd, J_d1_ = 8.5 Hz, J_d2_ = 6.2 Hz, J_d3_ = 4.4 Hz, 1H), 3.49 (s, 1H), 3.73 (d, J_d_ = 4.7 Hz, 2H), 3.76 (dd, J_d1_ = 11.2 Hz, J_d2_ = 8.5 Hz, 1H), 3.95 (dd, J_d1_ = 11.2 Hz, Jd_2_ = 4.4 Hz, 1H). Anal. Calcd for C_16_H_28_NO_3_: C, 68.05; H, 9.99; N, 4.96.; found: C, 67.99; H, 10.00; N, 4.87. HRMS (EI/DFS) *m*/*z*: [M]^+^calcd for C_16_H_28_NO_3_ 282.2064, found 282.2060.

*2,2-Diethyl-5-isopropyl-5-ethynyl-3,4-bis(hydroxymethyl)-pyrrolidine-1-oxyl* (**12c**). Yielding 0.137 g (51%) of yellow crystals. m.p. 132–133 °C (diethyl ether). IR (KBr) ν_max_: 3219 (≡C-H), 2100 (C≡C). ^1^H NMR (300 MHz; CD_3_OD, Zn/CF_3_COOH, δ): 0.98 (t, J_t_ = 7.4 Hz, 3H), 1.02 (t, J_t_ = 7.4 Hz, 3H), 1.14 (d, J_d_ = 6.5 Hz, 3H), 1.17 (d, J_d_ = 6.5 Hz, 3H), 1.93 (dq, J_d_ = 15.6 Hz, J_q_ = 7.4 Hz, 1H), 2.01–2.12 (m, 2H), 2.21 (dq, J_d_ = 13.7 Hz, J_q_ = 7.4 Hz, 1H), 2.38 (sp, J_sp_ = 6.5 Hz, 1H), 2.42 (ddd, J_d1_ = 3.8 Hz, J_d2_ = 3.8 Hz, J_d3_ = 3.1 Hz, 1H), 2.56 (ddd, J_d1_ = 10.0 Hz, J_d2_ = 3.8 Hz, J_d3_ = 4.9 Hz, 1H), 3.41 (s, 1H), 3.60 (dd, J_d1_ = 10.5 Hz, J_d2_ = 3.8 Hz, 1H), 3.74 (dd, J_d1_ = 11.1 Hz, J_d2_ = 10.0 Hz, 1H), 3.82 (dd, J_d1_ = 10.5 Hz, J_d2_ = 3.1 Hz, 1H), 3.92 (dd, J_d1_ = 11.1 Hz, J_d2_ = 4.9 Hz, 1H). Anal. calcd for C_15_H_26_NO_3_: C, 67.13; H, 9.76; N, 5.22.; found: C, 66.73; H, 9.79; N, 5.21.HRMS (EI/DFS) *m*/*z* [M]^+^calcd for C_15_H_26_NO_3_: 268.1907; found: 268.1909.

*(3S,4S,5S)-3,4-Di-tert-butoxy-2,2,5-triethyl-5-ethynylpyrrolidine 1-oxyl* (**14**). To the solution of **2** (0.7 g, 2.23 mmol) in dry THF (5 mL), the solution of ethynylmagnesium bromide in THF (22.3 mL of a 0.5M solution) was added in one portion. The mixture was allowed to stand at r.t. for one week. The reaction mixture was quenched with H_2_O (2 mL), and the inorganic precipitate was filtered off. The dry air was bubbled into the solution until the oxidation to radical was complete (TLC control, silica gel, EtOAc). The organic layer was concentrated in a vacuum, and the residue was purified using column chromatography (silica gel, EtOAc) to give **14** as an orange crystal, m.p. 91–93 °C (from hexane). Yield: 490 mg (65%), UV (EtOH): 244 (3,21). [α]^26^_D_ = + 106.1 (c 0.85, CHCl_3_). IR (KBr) ν_max_: 3271 (≡C-H). ^1^H NMR (500 MHz; CD_3_OD, Zn/CF_3_COOH, δ): 0.99 (t, J_t_ = 7.4 Hz, 3H), 1.03 (t, J_t_ = 7.4 Hz, 3H), 1.16 (t, J_t_ = 7.4 Hz, 3H), 1.31 (s, 9H), 1.33 (s, 9H), 1.90–2.01 (m, 4H), 2.05 (dq, J_d_ = 15.5 Hz, J_q_ = 7.4 Hz, 1H), 2.18 (dq, J_d_ = 14.0 Hz, J_q_ = 7.4 Hz, 1H), 3.27 (s, 1H), 4.02 (s, 1H), 4.20 (s, 1H). Anal. calcd for C_20_H_36_NO_3_: C, 70.96; H, 10.72; N, 4.14; found: C, 70.85; H, 10.65; N, 4.10.

*2,2,5-Triethyl-5-ethynylpyrrolidin-1-oxyl* (**15**). A solution of **3** (1.0 g, 5.9 mmol) in anhydrous THF (10 mL) was added to a 0.5–1 M solution of ethynyl-magnesium bromide in THF (50 mL) upon stirring. The mixture was allowed to stand at room temperature for 24 h (TLC control, hexane-ethyl acetate 4:1, UV detection), then quenched with NaCl saturated solution (10 mL). The organic layer was separated, and the residue was washed with ethyl acetate (2 × 20 mL). The combined organic layers were dried with anhydrous Na_2_SO_4_. The solvent was evaporated in a vacuum, and the crude residue was dissolved in methanol (15 mL) and basified with sodium hydroxide solution (1 M, 5 mL). Methylene blue (6 mg, 0.02 mmol) was added to the mixture, and the air was bubbled until the solution turned dark blue. The methanol was distilled off in a vacuum, and the remaining aqueous solution was extracted with ether (3 × 20 mL). The combined organic solution was washed with water (3 × 20 mL). The organic phase was dried with Na_2_SO_4,_ and the solvent was evaporated in a vacuum. The residue was purified by column chromatography on silica gel, eluent hexane–ethyl acetate 4:1, to give **15**, with a yield of 800 mg (70%), as a yellow liquid. IR (KBr) ν_max_: 3309, 3244 (≡C-H), 2107 (C≡C). ^1^H NMR (300 MHz; CD_3_OD, Zn/CF_3_COOH, δ): 0.99 (t, J_t_ = 7.4 Hz, 3H), 1.01 (t, J_t_ = 7.4 Hz, 3H), 1.18 (t, J_t_ = 7.4 Hz, 3H), 1.76–1.86 (m, 2H), 1.94–2.17 (m, 6H), 2.23–2.24 (m, 2H), 3.39 (s, 1H). Anal. calcd for C_12_H_20_NO: C, 74.16; H, 10.38; N, 7.21; found: C, 73.95; H, 10.65; N, 7.10.

*2,2,5-Triethyl-5-ethynyl-3,4,4-trimethylimidazolidin-1-oxyl* (**16**). A solution of **5** (850 mg, 4 mmol) in THF (5 mL) was added to a 0.9 M solution of ethynylmagnesium bromide (45 mL, 40 mmol) in THF, and the flask was sealed and left at 20 °C for 21 days. The reaction mixture was quenched with brine, the organic phase was separated, and the aqueous phase was washed with diethyl ether. Combined organic phases were dried with Na_2_CO_3,_ then PbO_2_ (10 g, 42 mmol) was added, and the reaction mixture was stirred for 24 h. The led oxides were filtered off, and the solution was concentrated in a vacuum and separated using column chromatography of silica gel, and the eluent hexane–diethyl ether 3:1 to give **16** as an orange oil, with a yield of 620 mg (65%). IR (KBr) ν_max_: 3309, 3251 (≡C-H), 2808 (C-H, NMe), 2112 (C≡C). For NMR investigation, the sample (10 mg) was stirred with Zn powder (100 mg) and ND_4_Cl (30 mg) in CD_3_OD (0.5 mL) at 5 °C for 10 min and filtered into an NMR tube. ^1^H NMR (300 MHz; CD_3_OD, δ): 0.97 (t, J_t_ = 7.4 Hz, 3H), 0.99 (t, Jt = 7.4 Hz, 3H), 1.06 (s, 3H), 1.12 (t, J_t_ = 7.4 Hz, 3H), 1.27 (s, 3H), 1.48 (dq, J_d_ = 13.6 Hz, J_q_ = 7.4 Hz, 1H), 1.67 (dq, J_d_ = 14.0 Hz, J_q_ = 7.4 Hz, 1H), 1.78 (dq, J_d_ = 13.6 Hz, J_q_ = 7.4 Hz, 1H), 1.87 (dq, J_d_ = 14.0 Hz, J_q_ = 7.4 Hz, 1H), 1.95 (dq, J_d_ = 14.0 Hz, J_q_ = 7.4 Hz, 1H), 2.13 (dq, J_d_ = 14.0 Hz, J_q_ = 7.4 Hz, 1H), 2.31 (s, 3H), 2.72 (s, 1H). HRMS (EI/DFS) *m*/*z* [M]^+^calcdforC_14_H_25_N_2_O: 237.1961; found: 237.1960.

*2,5,5-Triethyl-2-ethynyl-4-pyrrolidino-2,5-dihydroimidazol-1-oxyl* (**17**) was prepared using the above procedure. The reaction was completed in 10 h, with a yield of 70%, producing a yellow crystalline solid, m.p. 61–62 °C. IR (KBr) ν_max_: 3290 (≡C-H), 2104 (C≡C), 1583 (C=N). ^1^H NMR (400 MHz; CD_3_OD, Zn/ND_4_Cl, δ): 0.88 (t, J_t_ = 7.4 Hz, 3H), 1.00 (t, J_t_ = 7.4 Hz, 3H), 1.06 (t, J_t_ = 7.4 Hz, 3H), 1.73 (q, J_q_ = 7.4 Hz, 2H), 1.81 (dq, J_d_ = 13.6 Hz, J_q_ = 7.4 Hz, 1H), 1.84–2.00 (m, 6H) 2.06 (dq, J_d_ = 15.2 Hz, J_q_ = 7.4 Hz, 1H), 2.84 (s, 1H), 3.34–3.42 (m, 2H), 3.44–3.52 (m, 2H). Anal. calcd for C_15_H_24_N_3_O: C, 68.67; H, 9.22; N, 16.02; found: C, 68.34; H, 9.11; N, 15.89.

#### 3.2.5. General Method of Hydrogenation

A solution of ethynyl-substituted nitroxide (10 mmol) in THF (100 mL) was placed in the reaction vessel equipped with a magnetic stirrer and a connection line to a gasometer filled with hydrogen. The catalyst (Pd/C, 4%, 200 mg) was added, and the system was purged with hydrogen and closed. The mixture was vigorously stirred until hydrogen absorption ceased (ca. 5 h, 0.6 L of hydrogen absorbed), after which the catalyst was filtered off and washed with THF. The THF was distilled off in a vacuum, and the residue was dissolved in methanol (20 mL). A 1 M solution of sodium hydroxide in water (6 mL) and methylene blue (3 mg, 0.01 mmol) was added to the mixture, and the air was bubbled until the solution turned dark blue. Then the mixture was diluted with water (20 mL) and acidified with a 1 M solution of sulfuric acid (4 mL). Methanol was evaporated in a vacuum. The mixture was extracted with ethyl acetate (3 × 10 mL). The organic phase was evaporated in a vacuum, and the residue was purified using column chromatography on silica gel (hexane–ethyl acetate 1:1).

*2,2,5-Triethyl-5-tert-butyl-3,4-bis(hydroxymethyl)-pyrrolidine-1-oxyl* (**13b**). Yield 2.35 g (82%), yellow crystals, m.p. 109–111 °C (from hexane). IR(KBr) ν_max_: 3373, 3294 (O-H). ^1^H NMR (300 MHz; CD_3_OD, Zn/CF_3_COOH, δ): 1.08 (t, J_t_ = 7.4 Hz, 3H), 1.12 (t, J_t_ = 7.4 Hz, 3H), 1.15 (t, J_t_ = 7.4 Hz, 3H), 1.19 (s, 9H), 1.77–2.20 (m, 6H), 2.42 (ddd, J_d1_ = 12.3 Hz, J_d2_ = 6.2 Hz, J_d3_ = 5.5 Hz, 1H), 2.54 (ddd, J_d1_ = 12.3 Hz, J_d2_ = 5.3 Hz, J_d3_ = 1.5 Hz, 1H), 3.79 (dd, J_d1_ = 11.5 Hz, J_d2_ = 5.3 Hz, 1H), 3.82 (dd, J_d1_ = 11.1 Hz, J_d2_ = 6.2 Hz, 1H), 3.85 (dd, J_d1_ = 11.1 Hz, J_d2_ = 5.5 Hz, 1H), 3.94 (dd, J_d1_ = 11.5 Hz, J_d2_ = 1.5 Hz, 1H). Anal. calcd for C_16_H_32_NO_3_: C, 67.09;H, 11.26; N, 4.89; found: C, 67.44; H, 11.51; N, 4.84. HRMS (EI/DFS) *m*/*z* [M]^+^calcd for C_16_H_32_NO_3_: 286.2377; found: 286.2380.

*2,2,5-Triethyl-5-isopropyl-3,4-bis(hydroxymethyl)-pyrrolidine-1-oxyl* (**13c**). Yield 1.39 g (51%), yellow oil. IR(neat) ν_max_: 2966, 2941, 2861 (C-H). ^1^H NMR (400 MHz; CD_3_OD, Zn/CF_3_COOH, δ): 1.03 (t, J_t_ = 7.4 Hz, 3H), 1.04 (t, J_t_ = 7.4 Hz, 3H), 1.06 (t, J_t_ = 7.4 Hz, 3H), 1.10 (d, J_d_ = 6.9 Hz, 3H), 1.11 (d, J_d_ = 6.9 Hz, 3H), 1.78 (dq, J_d_ = 14.7 Hz, J_q_ = 7.4 Hz, 1H), 1.81 (dq, J_d_ = 15.1 Hz, J_q_ = 7.4 Hz, 1H), 1.91 (dq, J_d_ = 15.1 Hz, J_q_ = 7.4 Hz, 1H), 1.92 (q, J_q_ = 7.4 Hz, 2H), 1.99 (dq, J_d_ = 14.7 Hz, J_q_ = 7.4 Hz, 1H), 2.26 (sep, J_sep_ = 6.9 Hz, 1H), 2.36–2.51 (m, 2H), 3.67–3.85 (m, 4H). Anal.calcd for C_15_H_30_NO_3_: C, 66.14; H, 11.10; N, 5.14; found: C, 66.35; H, 10.98; N, 5.07. HRMS (EI/DFS) *m*/*z* [M]^+^calcd for C_15_H_30_NO_3_: 272.2220; found: 272.2217.

*(3S,4S)-3,4-Di-tert-butoxy-2,2,5,5-tetraethylpyrrolidine 1-oxyl* (**18**). A solution of **14** (0.5 g, 1.48 mmol) in THF (10 mL) was placed in the reaction vessel equipped with a magnetic stirrer and a connection line to a gasometer filled with hydrogen. The catalyst (Pd/C, 4%, 100 mg) was added, and the system was purged with hydrogen and closed. The mixture was vigorously stirred until hydrogen absorption ceased (ca. 5 h, 66 mL of hydrogen absorbed). The catalyst was filtered off, the organic layer was concentrated in a vacuum, and the residue was purified using column chromatography (silica gel, EtOAc–hexane 1:1) to give **18** as yellow crystals, m.p. 117–119 °C (from hexane). Yield: 405 mg (80%).UV (EtOH): 241 (3,22). [α]^26^_D_ = +128.1 (c 0.85, CHCl_3_). IR (KBr): 2966, 2937, 2879 (C-H). ^1^H NMR (500 MHz, CD_3_OD, Zn/CF_3_COOH, δ): 0.86 (t, J_t_ = 7.4 Hz, 6H), 0.90 (t, J_t_ = 7.4 Hz, 6H), 1.20 (s, 18H), 1.63 (dq, J_d_ = 15.7 Hz, J_q_ = 7.4 Hz, 2H), 1.84 (dq, J_d_ = 15.7 Hz, J_q_ = 7.4 Hz, 2H), 1.85 (dq, J_d_ = 14.0 Hz, J_q_ = 7.4 Hz, 2H), 2.24 (dq, J_d_ = 14.0 Hz, J_q_ = 7.4 Hz, 2H), 3.93 (s, 2H). ^13^C{^1^H} NMR (125 MHz, CD_3_OD, Zn/CF_3_COOH,δ): 8.0, 9.1, 25.8, 26.6, 29.0, 76.6, 76.7, 80.9. Anal. calcd for C_20_H_40_NO_3_: C, 70.13; H, 11.77; N, 4.09; found: C, 70.25; H, 11.65; N, 4.21.

*2,2,5,5-Tetraethylpyrrolidin-1-oxyl* (**19**). A solution of **15** (0.8 g, 4.1 mmol) in dry THF (10 mL) was placed in the reaction vessel equipped with a magnetic stirrer and a connection line to a gasometer filled with hydrogen. The catalyst (Pd/C, 4%, 30 mg) was added, and the system was purged with hydrogen and closed. The mixture was vigorously stirred until hydrogen absorption ceased (ca. 5 h, 0.25 L of hydrogen absorbed), then the catalyst was filtered off and washed with THF. The filtrate was evaporated in a vacuum, and the crude residue was dissolved in methanol (15 mL) and basified with sodium hydroxide solution (1 M, 5 mL). Methylene blue (6 mg, 0.02 mmol) was added to the mixture, and the air was bubbled until the solution turned dark blue. The methanol was distilled off in a vacuum, and the remaining aqueous solution was extracted with ether (3 × 20 mL). The organic phase was dried with Na_2_SO_4_, and the solvent was evaporated in a vacuum. The residue was purified by column chromatography (silica gel, eluent hexane–ethyl acetate 4:1) to give **19**, with a yield of 800 mg (70%), yellow liquid. IR (neat, cm^−^): 2966, 2937, 2881 (C-H). ^1^H NMR (300 MHz; CD_3_OD, Zn/CF_3_COOH, δ): = 0.97 (t, J_t_ = 7.4 Hz, 12H), 1.75 (dq, J_d_ = 14.0 Hz, J_q_ = 7.4 Hz, 4H), 1.84 (dq, J_d_ = 14.0 Hz, J_q_ = 7.4 Hz, 4H), 1.98 (s, 4H). Anal. calcd for C_12_H_24_NO: C, 72.20; H, 12.20; N, 7.06; found: C, 72.25; H, 12.35; N, 7.21. HRMS (EI/DFS) *m*/*z* [M]^+^calcd for C_12_H_24_NO: 198.1852, found: 198.1851.

*2,2,5,5-Tetraethyl-3,4,4-trimethylimidazolidin-1-oxyl* (**20**). A solution of **16** (355 mg, 1.5 mmol) in THF (2 mL) was placed in the reaction vessel equipped with a magnetic stirrer and a connection line to a gasometer filled with hydrogen. The catalyst (Pd/C, 4%, 50 mg) was added, and the system was purged with hydrogen and closed. The mixture was vigorously stirred until hydrogen absorption ceased (ca. 5 h, 100 mL of hydrogen absorbed), then the catalyst was filtered off and washed with THF. The solution was bubbled with air overnight, THF was distilled off in a vacuum, and the residue was separated using column chromatography on silica gel, eluent hexane–diethyl ether 3:1 to give **20** as a yellow oil, with a yield of 620 mg (65%). IR (KBr) ν_max_: 2972, 2943, 2881 (C-H). For the NMR investigation, the sample (10 mg) was stirred with Zn powder (100 mg) and ND_4_Cl (30 mg) in CD_3_OD (0.5 mL) at 5 °C for 10 min and filtered into an NMR tube. ^1^H NMR (300 MHz; CD_3_OD, Zn/ND_4_Cl,δ): = 0.94 (m, 12H), 1.04 (s, 6H), 1.55–1.80 (m, 6H), 1.82–1.96 (m, 2H), 2.31 (s, 3H). HRMS (EI/DFS) *m*/*z* [M]^+^calcd. forC_14_H_25_N_2_O: 237.1961; found: 237.1960.

*2,2,5,5-Tetraethyl-4-pyrrolidino-2,5-dihydroimidazol-1-oxyl* (**21**) was prepared using the above procedure, with a yield of 94%.IR(KBr) ν_max_: 2968, 2939, 2877 (C-H), 1593 (C=N). ^1^H NMR (400 MHz; CD_3_OD, Zn/ND_4_Cl, δ): 0.96 (t, J_t_ = 7.4 Hz, 6H), 0.97 (t, J_t_ = 7.4 Hz, 6H), 1.70 (dq, J_d_ = 14.2 Hz, J_q_ = 7.4 Hz, 2H), 1.81 (dq, J_d_ = 14.6 Hz, J_q_ = 7.4 Hz, 2H), 1.82 (dq, J_d_ = 14.2 Hz, J_q_ = 7.4 Hz, 2H), 1.92 (dq, J_d_ = 14.6 Hz, J_q_ = 7.4 Hz, 2H), 1.94–1.99 (m, 4H), 3.46–3.51 (m, 4H).

#### 3.2.6. General Method of Reaction of Nitrones with Alkynyl Magnesium Bromides

The terminal alkyne (0.107 mol) was added dropwise to a 2 M solution of ethylmagnesium bromide in THF (50 mL, 0.100 mol). The mixture was stirred at ambient temperature for 1 h. Then a solution of **1a** (3.7 g, 0.01 mol) in dry THF (10 mL) was added to the mixture and kept at ambient temperature for 2 days (TLC control on silica gel, eluent ethyl acetate, visualization with UV-254 and Dragendorff’s reagent). Then the mixture was quenched with water, the organic phase was separated via decantation, and the remaining wet precipitate was washed with THF 5 × 10 mL. The THF was distilled off in a vacuum, and the residue was dissolved in methanol (50 mL). A 1 M solution of sodium hydroxide in water (10 mL) was added to the mixture. Then, the methylene blue (3 mg, 0.01 mmol) was added to the mixture, and the air was bubbled until the solution turned dark blue. Then the mixture was diluted with water (50 mL) and a 1M solution of sulfuric acid (6 mL). Methanol was distilled off in a vacuum, and the mixture was extracted with ethyl acetate (3 × 10 mL). The organic phase was concentrated in a vacuum, and the resulting crude nitroxide **22** was purified as described below.

*2,2,5-Triethyl-5-phenylethynyl-3,4-bis(hydroxymethyl)-pyrrolidine-1-oxyl* (**22a**) was purified using column chromatography on silica gel (hexane–ethyl acetate 1:1). Yield 1.93 g (54%), yellow crystals, m.p. 100–103 °C (dec.) (from diethyl ether). IR (KBr) ν_max_: 3384 (O-H), 3311 (O-H), 1055 (C-OH). ^1^H NMR (400 MHz, CD_3_OD, Zn/CF_3_COOH, δ): 0.98 (t, J_t_ = 7.5 Hz, 3H), 1.01 (t, J_t_ = 7.5 Hz, 3H), 1.252 (t, J_t_ = 7.5 Hz, 3H), 1.86 (dq, J_d_ = 14.9 Hz, J_q_ = 7.5 Hz, 1H), 1.88 (dq, J_d_ = 14.9 Hz, Jq = 7.5 Hz, 1H), 2.10 (dq, J_d_ = 14.1 Hz, J_q_ = 7.2 Hz, 1H), 2.15 (dq, J_d_ = 14.5 Hz, J_q_ = 7.3 Hz, 1H), 2.17 (dq, J_d_ = 14.1 Hz, J_q_ = 7.9 Hz, 1H), 2.25 (dq, J_d_ = 14.5 Hz, J_q_ = 7.7 Hz, 1H), 2.39 (ddd, J_d1_ = 10.2 Hz, J_d2_ = 6.6 Hz, J_d3_ = 4.5 Hz, 1H), 2.41 (ddd, J_d1_ = 10.2 Hz, J_d2_ = 5.2 Hz, J_d3_ = 3.5 Hz, 1H), 3.71 (dd, J_d1_ = 11.5 Hz, J_d2_ = 5.2 Hz, 1H), 3.78 (dd, J_d1_ = 11.5 Hz, J_d2_ = 3.5 Hz, 1H), 3.86 (dd, J_d1_ = 11.6 Hz, J_d2_ = 6.6 Hz, 1H), 3.97 (dd, J_d1_ = 11.6 Hz, J_d2_ = 4.5 Hz, 1H), 7.38 (dddd, J_d1_ = 7.8 Hz, J_d2_ = 7.6 Hz, J_d3_ = 1.3 Hz, J_d4_ = 0.6 Hz, 2H), 7.42 (dddd, J_d1_ = 7.6 Hz, J_d2_ = 7.6 Hz, Jd3 = 1.3 Hz, J_d4_ = 1.3 Hz, 1H), 7.46 (dddd, J_d1_ = 7.8 Hz, J_d2_ = 1.8 Hz, J_d3_ = 1.3 Hz, J_d4_ = 0.6 Hz, 2H). Anal. calcd for C_20_H_28_NO_3_: C, 72.69; H, 8.54; N, 4.24.; found: C, 72.75; H, 8.54; N, 4.46. HRMS (EI/DFS) *m*/*z* [M]^+^calcd for C_20_H_28_NO_3_: 330.2064; found: 330.2062.

*2,2,5-Triethyl-5-(3-hydroxyprop-1-yn-1-yl)-3,4-bis(hydroxymethyl)-pyrrolidine-1-oxyl* (**22b**) was crystallized from diethyl ether. Yield 1.7 g (60%), yellow crystals, m.p. 104–105 °C. IR (KBr) ν_max_: 3493, 3412, 3365 (O-H), 1045 (C-OH). ^1^H NMR (300 MHz, CD_3_OD, Zn/CF_3_COOH, δ): 0.94 (t, J_t_ = 7.5 Hz, 3H), 1.14 (t, J_t_ = 7.4 Hz, 3H), 1.55 (t, J_t_ = 7.2 Hz, 3H), 1.77 (dq, J_d_ = 15.5 Hz, J_q_ = 7.4 Hz, 1H), 1.80 (dq, J_d_ = 15.5 Hz, J_q_ = 7.4 Hz, 1H), 1.98 (dq, J_d_ = 13.8 Hz, J_q_ = 7.5 Hz, 1H), 2.04 (dq, J_d_ = 14.4 Hz, J_q_ = 7.2 Hz, 1H), 2.07 (dq, J_d_ = 13.8 Hz, J_q_ = 7.5 Hz, 1H), 2.08 (dq, J_d_ = 14.4 Hz, J_q_ = 7.2 Hz, 1H), 2.28 (ddd, J_d1_ = 9.8 Hz, J_d2_ = 6.5 Hz, J_d3_ = 5.1 Hz, 1H), 2.30 (ddd, J_d1_ = 9.8 Hz, J_d2_ = 5.9 Hz, J_d3_ = 3.7 Hz, 1H), 3.64 (dd, J_d1_ = 11.3 Hz, J_d2_ = 5.9 Hz, 1H), 3.74 (dd, J_d1_ = 11.3 Hz, J_d2_ = 3.7 Hz, 1H), 3.76 (dd, J_d1_ = 11.4 Hz, J_d2_ = 6.5 Hz, 1H), 3.85 (dd, J_d1_ = 11.4 Hz, J_d1_ = 5.1 Hz, 1H). Anal. calcd for C_15_H_26_NO_4_: C, 63.35; H, 9.22; N, 4.93.; found: C, 62.85; H, 9.25; N, 4.86. HRMS (EI/DFS) *m*/*z*: [M]^+^calcdfor C_15_H_26_NO_4_: 284.1856; found 284.1851.

*2,2,5-Triethyl-5-(3-hydroxy-3-methylbut-1-yn-1-yl)-3,4-bis(hydroxymethyl)-pyrrolidine-1-oxyl* (**22c**) was crystallized from diethyl ether to give **22c**. Yield 2.0 g (66%) of yellow crystals, m.p. 111–114 °C. IR (KBr) ν_max_: 3340, 3182 (O-H). ^1^H NMR (300 MHz, CD_3_OD, Zn/CF_3_COOH, δ): 0.96 (t, J_t_ = 7.4 Hz, 3H), 0.97 (t, J_t_ = 7.6 Hz, 3H), 1.16 (t, J_t_ = 7.3 Hz, 3H), 1.50 (s, 6H), 1.80 (dq, J_d_ = 14.1 Hz, J_q_ = 7.4 Hz, 1H), 1.87 (dq, J_d_ = 14.1 Hz, J_q_ = 7.2 Hz, 1H), 1.98 (dq, J_d_ = 14.3 Hz, J_q_ = 7.7 Hz, 1H), 2.06 (dq, J_d_ = 15.2 Hz, J_q_ = 7.3 Hz, 1H), 2.11 (dq, J_d_ = 14.1 Hz, J_q_ = 7.2 Hz, 1H), 2.13 (dq, J_d_ = 14.3 Hz, J_q_ = 7.5 HZ, 1H), 2.29 (dd, J_d1_ = 4.4 Hz, J_d2_ = 3.1, 1H), 2.29 (dd, J_d1_ = 5.7, J_d2_ = 4.4, 1H) 3.66 (dd, J_d1_ = 11.7 Hz, J_d2_ = 4.4 Hz, 1H), 3.74 (dd, J_d1_ = 11.7 Hz, J_d2_ = 3.1 Hz, 1H), 3.76 (dd, J_d1_ = 11.6 Hz, J_d2_ = 5.7 Hz, 1H), 3.86 (dd, J_d1_ = 11.6 Hz, J_d2_ = 4.4 Hz, 1H). Anal. calcd for C_17_H_30_NO_4_: C, 65.35; H, 9.68; N, 4.48.; found: C, 65.82; H, 9.65; N, 4.52. HRMS (EI/DFS) *m*/*z* [M]^+^calcdfor C_17_H_30_NO_4_: 312.2169; found 312.2164.

*2,2,5-Triethyl-5-(3-hydroxyprop-1-yn-1-yl)pyrrolidin-1-oxyl* (**23**). A solution of 2-propyn-1-ol (1.75 mL, 29.6 mmol) in dry THF (5 mL)was slowly added to the ethylmagnesium bromide solution in THF (2.0 M, 30 mL) at 0 °C under argon atmosphere. The resulting grey mixture was stirred for 30 min at room temperature. After that, nitrone**3** solution (0.5 g, 3.0 mmol) in 5 mL of anhydrous THF was added there. The final mixture was stirred for 20 h at room temperature under an argon atmosphere and then poured into a mixture of ice (10 g) and NaCl (10 g). The organic layer was separated, and the residue was washed with ethyl acetate (3 × 30 mL). The combined organic phase was dried with Na_2_SO_4,_ and the solvent was evaporated in a vacuum. The crude residue was dissolved in methanol (10 mL) and basified with sodium hydroxide solution (1 M, 2 mL). Methylene blue (3 mg, 0.01 mmol) was added to the mixture, and the air was bubbled until the solution turned dark blue. The methanol was distilled off in a vacuum, and the remaining aqueous solution was extracted with ether (3 × 20 mL). The organic phase was dried with Na_2_SO_4,_ and the solvent was evaporated in a vacuum. The residue was purified by column chromatography (silica gel, eluent hexane–ethyl acetate 1:1) to give **23**, with a yield of 400 mg (65%), yellow liquid. IR (neat) ν_max_: 3417 (O-H), 2970, 2939, 2879 (C-H). ^1^H NMR (400 MHz; CD_3_OD, Zn/CF_3_COOH, δ): 0.99 (t, J_t_ = 7.4 Hz, 3H), 1.01 (t, J_t_ = 7.4 Hz, 3H), 1.17 (t, J_t_ = 7.4 Hz, 3H), 1.76–1.83 (m, 2H), 1.90–2.13 (m, 6H), 2.23–2.37 (m, 2H), 4.29 (s, 2H). HRMS (EI/DFS) *m*/*z* [M]^+^calcd for C_13_H_22_NO_2_: 224.1645; found: 224.1642

## 4. Conclusions

The synthesis of nitroxides via reaction of 1-pyrroline 1-oxides with alkynylmagnesium halides was earlier investigated by K. Hideg et al. [34,35,36]. Recently, we suggested using this reaction for the preparation of sterically shielded 2,2,5,5-tetraalkylpyrrolidine nitroxides [8,21]. In this paper, we showed how these reagents could be applied for the preparation of various highly strained nitroxides of pyrrolidine, imidazolidine and 2,5-dihydroimidazole series. The described two-step addition & hydrogenation protocol was suitable for the introduction of an ethyl group to the carbon atom of cyclic *α*-ethyl-, α-isopropyl- and α-*tert*-butyl nitrones and may find broader application. Some of the new nitroxides can hardly be prepared in any other way.

The new data on the feasibility of the addition of alkynylmagnesium halides to highly hindered alkylnitrones may have another consequence. In this work, we did not utilize the synthetic potential of alkynyl groups. However, taking into account the broad application of alkynes in organic synthesis, the use of 2-alkynyl nitroxides for biorthogonal spin labeling and for the synthesis of bioactive nitroxide derivatives certainly deserves attention.

The new set of nitroxides gives impressive examples of the variability of EPR spectra of five-membered nitroxides with bulky alkyl substituents adjacent to the nitroxide group. The high additional coupling constants are highly dependent on nitroxide structure. The high sensitivity of spectral parameters to minor structural changes is a promising basis for the molecular design of functional spin probes of a new generation.

## Figures and Tables

**Table 1:** Synthesis of nitroxides from nitrones: reaction times and yields (for conditions, see the Section 3).

Nitrone	Addition of Ethynylmagnesium Bromide			Hydrogenation		Overall Yield, %
	Time	Product	Yield, %		Yield, %	
**1a**	7 days	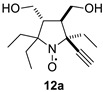	68	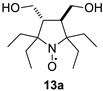	81	55 *
**1b**	72 days	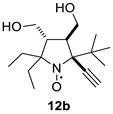	67	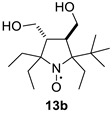	82	43 *
**1c**	14 days	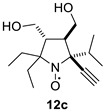	51	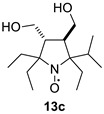	76	39
**1d**	7 days	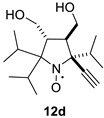	0			
**2**	7 days	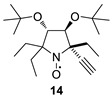	65	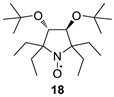	80	52
**3**	1 day	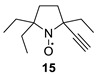	70	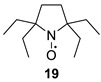	70	49
**4**	21 days	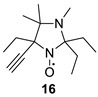	65	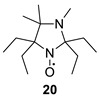	74	48
**5**	10 h	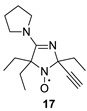	70	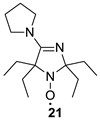	94	66

* From **8a**,**b**.

**Table 2:** Parameters of the EPR spectra of nitroxides in oxygen-free distilled water at a concentration of 0.2 mM. Modulation amplitude 0.5 G, MW power 5 mW.

Nitroxide	*a*_N_, G ±0.05	*a*_H_, G ±0.05	Line Width, G ±0.05
**12a**	15.8	2.24	0.87
**12b**	15.8	2.11	0.92
**12c**	15.8	2.22	0.96
**13a**	15.7	2.24; 2.24	1.25
**13b**	15.8	2.55	1.13
**13c**	15.6	2.52; 3.31	1.43
**14** *	14.9	1.74	0.97
**15**	16.1	-	0.73
**16**	16.4	-	1.03
**17**	15.6	-	0.90
**18** *	14.9	-	1.72
**19**	16.2	-	0.92
**20**	15.8	3.64	1.38
**20-H**^+^ **	14.7	4.01	1.49
**21**	15.7	-	0.89
**22a**	15.8	2.12	0.91
**22b**	15.7	2.09	0.89
**22c**	15.8	2.08	0.88
**23**	16.1	-	0.71
**24**	15.7	2.50; 1.88	1.24

* 0.1 mM in water-ethanol 1:1; ** 0.2 mM in 0.1 M phosphoric acid.

## Data Availability

All the data are in the text and the Supplementary Information in this article.

## Supplementary Materials

The following supporting information can be downloaded at: https://www.mdpi.com/article/10.3390/molecules27217626/s1, IR and NMR spectra of all new compounds, X-ray diffraction data for **8d**, **12a**–**c**, **14**, **22a**–**c**, EPR spectra of all new nitroxides, HPLC chromatogram of **14**.
