# Supplementary material for: Synthesis of Sterically Shielded Nitroxides Using the Reaction of Nitrones with Alkynylmagnesium Bromides
Source: Molecules. 2022 Nov 7;27(21):7626. doi: 10.3390/molecules27217626 (PMC9654931; doi:10.3390/molecules27217626)

Supporting Information

Synthesis of sterically shielded nitroxides using the reaction of nitrones with alkynylmagnesium bromides

Sergey A. Dobrynin[1], Mark M. Gulman [1,2], Denis A. Morozov[1*], Irina F. Zhurko[1], Andrey I. Taratayko[1], Yulia S. Sotnikova[1], Yurii I. Glazachev[3], Yuri V. Gatilov[1], Igor A. Kirilyuk[1]

[1]*N. N. Vorozhtsov Novosibirsk Institute of Organic Chemistry SB RAS, Lavrentiev Ave. 9, Novosibirsk, 630090, Russia*

[2]*Novosibirsk State University, Pirogova Str. 2, Novosibirsk, 630090, Russia*

[3]*Voevodsky Institute of Chemical Kinetics and Combustion SB RAS, Institutskaya 3, Novosibirsk, 630090, Russia*

Table of contents

Spectra 5

^{1}H NMR of 2,2-diethyl-5-isopropyl-3,4-bis(methoxycarbonyl)pyrrolidine (6c)..........5

^{13}C{^{1}H} NMR of 2,2-diethyl-5-isopropyl-3,4-bis(methoxycarbonyl)pyrrolidine (6c) 5

^{1}H NMR of 2,2-diethyl-5-isopropyl-3,4-bis(methoxycarbonyl)pyrrolidine (7c)..........6

^{13}C{^{1}H} NMR of 2,2-diethyl-5-isopropyl-3,4-bis(methoxycarbonyl)pyrrolidine (7c) 6

^{1}H NMR of 2,2,5-triisopropyl-3,4-bis(methoxycarbonyl)pyrrolidine (6d) 7

^{13}C{^{1}H} NMR of 2,2,5-triisopropyl-3,4-bis(methoxycarbonyl)pyrrolidine (6d)..........7

^{1}H NMR of 2,2,5-triisopropyl-3,4-bis(methoxycarbonyl)pyrrolidine (7d) 8

^{13}C{^{1}H} NMR of 2,2,5-triisopropyl-3,4-bis(methoxycarbonyl)pyrrolidine (7d)..........8

^{1}H NMR of 2,2-diethyl-5-isopropyl-3,4-bis(hydroxymethyl)-3,4-dihydro-2H-pyrrole 1-oxide (8c)..........9

^{13}C{^{1}H} NMR of 2,2-diethyl-5-isopropyl-3,4-bis(hydroxymethyl)-3,4-dihydro-2H-pyrrole 1-oxide (8c) ...9

^{1}H NMR of 2,2,5-triisopropyl-3,4-bis(hydroxymethyl)-3,4-dihydro-2H-pyrrole 1-oxide (8d) 10

^{13}C{^{1}H} NMR of 2,2,5-triisopropyl-3,4-bis(hydroxymethyl)-3,4-dihydro-2H-pyrrole 1-oxide (8d)..........10

^{1}H NMR of 2,2,5-triethyl-3,4-bis(((2-methoxypropan-2-yl)oxy)methyl)-3,4-dihydro-2H-pyrrole 1-oxide (1a) 11

^{13}C{^{1}H} NMR of 2,2,5-triethyl-3,4-bis(((2-methoxypropan-2-yl)oxy)methyl)-3,4-dihydro-2H-pyrrole 1-oxide (1a)..........11

^{1}H NMR of 2,2-diethyl-5-isopropyl-3,4-bis(((2-methoxypropan-2-yl)oxy)methyl)-3,4-dihydro-2H-pyrrole 1-oxide (1c) 12

^{13}C{^{1}H} NMR of 2,2-diethyl-5-isopropyl-3,4-bis(((2-methoxypropan-2-yl)oxy)methyl)-3,4-dihydro-2H-pyrrole 1-oxide (1c) 12

^{1}H NMR of 5-tert-butyl-2,2-diethyl-3,4-bis(((2-methoxypropan-2-yl)oxy)methyl)-3,4-dihydro-2H-pyrrole 1-oxide (1b)....13

^{13}C{^{1}H} NMR of 5-tert-butyl-2,2-diethyl-3,4-bis(((2-methoxypropan-2-yl)oxy)methyl)-3,4-dihydro-2H-pyrrole 1-oxide (1b)....13

^{1}H NMR of 2,2,5-triisopropyl-3,4-bis(((2-methoxypropan-2-yl)oxy)methyl)-3,4-dihydro-2H-pyrrole 1-oxide (1d) 14

^{13}C{^{1}H} NMR of 2,2,5-triisopropyl-3,4-bis(((2-methoxypropan-2-yl)oxy)methyl)-3,4-dihydro-2H-pyrrole 1-oxide (1d) 14

^{1}H NMR of 2,2,4-triethyl-5,5-dimethyl-2,5-dihydroimidazole 3-oxide (11) 15

^{13}C{^{1}H} NMR of 2,2,4-triethyl-5,5-dimethyl-2,5-dihydroimidazole 3-oxide (11)....15

^{1}H NMR of 2,2,4-triethyl-1,5,5-trimethyl-2,5-dihydroimidazole 3-oxide (4) 16

^{13}C{^{1}H} NMR of 2,2,4-triethyl-1,5,5-trimethyl-2,5-dihydroimidazole 3-oxide (4) 16

^{1}H NMR of (3S,4S)-3,4-di-tert-butoxy-2,2,5-triethyl-3,4-dihydro-2H-pyrrole 1-oxide (2) 17

^{13}C{^{1}H} NMR of (3S,4S)-3,4-di-tert-butoxy-2,2,5-triethyl-3,4-dihydro-2H-pyrrole 1-oxide (2) 17

^{1}H NMR of 2,2,5-triethyl-5-ethynyl-3,4-bis(hydroxymethyl)-pyrrolidine-1-oxyl (12a) 18

^{1}H NMR of 2,2-diethyl-5-isopropyl-5-ethynyl-3,4-bis(hydroxymethyl)-pyrrolidine-1-oxyl (12c)....18

^{1}H NMR of 5-tert-dutyl-2,2-diethyl-5-ethynyl-3,4-bis(hydroxymethyl)-pyrrolidine-1-oxyl (12b)....19

^{1}H NMR of 2,2,5,5-tetraethyl-3,4-bis(hydroxymethyl)-pyrrolidine-1-oxyl (13a) 19

^{1}H NMR of 2,2,5-triethyl-5-isopropyl-3,4-bis(hydroxymethyl)-pyrrolidine-1-oxyl (13c)....20

^{1}H NMR of 2,2,5-triethyl-5-tert-butyl-3,4-bis(hydroxymethyl)-pyrrolidine-1-oxyl (13b)....20

^{1}H NMR of 2,5,5-triethyl-2-ethynyl-4-pyrrolidino-2,5-dihydroimidazol-1-oxyl (17) 21

^{1}H NMR of 2,2,5,5-tetraethyl-4-pyrrolidino-2,5-dihydroimidazol-1-oxyl (21) 21

^{1}H NMR of 2,2,5-triethyl-5-ethynyl-3,4,4-trimethylimidazolidin-1-oxyl (16)....22

^{1}H NMR of 2,2,5,5-tetraethyl-3,4,4-trimethylimidazolidin-1-oxyl (20) 22

^{1}H NMR of 2,2,5-triethyl-5-ethynylpyrrolidin-1-oxyl (15) 23

^{1}H NMR of 2,2,5,5-tetraethylpyrrolidin-1-oxyl (19) 23

^{1}H NMR of (3S,4S,5S)-3,4-di-tert-butoxy-2,2,5-triethyl-5-ethynylpyrrolidine 1-oxyl (14) 24

^{1}H NMR of (3S,4S)-3,4-di-tert-butoxy-2,2,5,5-tetraethylpyrrolidine 1-oxyl (18) 24

^{1}H NMR of 2,2,5-triethyl-5-(3-hydroxyprop-1-yn-1-yl)pyrrolidin-1-oxyl (23) 25

^{1}H NMR of 2,2,5-triethyl-5-phenylethynyl-3,4-bis(hydroxymethyl)-pyrrolidine-1-oxyl (22a) 25

^{1}H NMR of 2,2,5-triethyl-5-(3-hydroxyprop-1-yn-1-yl)-3,4-bis(hydroxymethyl)-pyrrolidine-1-oxyl (22b) 26

^{1}H NMR of 2,2,5-triethyl-5-(3-hydroxy-3-methylbut-1-yn-1-yl)-3,4-bis(hydroxymethyl)-pyrrolidine-1-oxyl (22c) 26

IR (neat) of 2,2-diethyl-5-isopropyl-3,4-bis(methoxycarbonyl)pyrrolidine (6c)....27

IR (neat) of 2,2-diethyl-5-isopropyl-3,4-bis(methoxycarbonyl)pyrrolidine (7c)....27

IR (neat) of 2,2,5-triisopropyl-3,4-bis(methoxycarbonyl)pyrrolidine (6d) 27

IR (neat) of 2,2,5-triisopropyl-3,4-bis(methoxycarbonyl)pyrrolidine (7d) 28
IR (KBr) of 2,2-diethyl-5-isopropyl-3,4-bis(hydroxymethyl)-3,4-dihydro-2H-pyrrole 1-oxide (8c) 28
IR (KBr) of 2,2,5-triisopropyl-3,4-bis(hydroxymethyl)-3,4-dihydro-2H-pyrrole 1-oxide (8d) 28
IR (neat) of 2,2,5-triethyl-3,4-bis(((2-methoxypropan-2-yl)oxy)methyl)-3,4-dihydro-2H-pyrrole 1-oxide (1a) 29
IR (neat) of 2,2-diethyl-5-isopropyl-3,4-bis(((2-methoxypropan-2-yl)oxy)methyl)-3,4-dihydro-2H-pyrrole 1-oxide (1c) 29
IR (KBr) of 5-tert-butyl-2,2-diethyl-3,4-bis(((2-methoxypropan-2-yl)oxy)methyl)-3,4-dihydro-2H-pyrrole 1-oxide (1b) 29
IR (KBr) of 2,2,5-triisopropyl-3,4-bis(((2-methoxypropan-2-yl)oxy)methyl)-3,4-dihydro-2H-pyrrole 1-oxide (1d) 30
IR (KBr) of (3S,4S)-3,4-di-tert-butoxy-2,2,5-triethyl-3,4-dihydro-2H-pyrrole 1-oxide (2) 30
IR (KBr) of 2,2,4-triethyl-5,5-dimethyl-2,5-dihydroimidazole 3-oxide (11) 30
IR (neat) of 2,2,4-triethyl-1,5,5-trimethyl-2,5-dihydroimidazole 3-oxide (4) 31
IR (KBr) of 2,2,5-triethyl-5-ethynyl-3,4-bis(hydroxymethyl)-pyrrolidine-1-oxyl (12a) 31
IR (KBr) of 2,2-diethyl-5-isopropyl-5-ethynyl-3,4-bis(hydroxymethyl)-pyrrolidine-1-oxyl (12c) 31
IR (KBr) of 5-tert-dutyl-2,2-diethyl-5-ethynyl-3,4-bis(hydroxymethyl)-pyrrolidine-1-oxyl (12b) 32
IR (KBr) of 2,2,5,5-tetraethyl-3,4-bis(hydroxymethyl)-pyrrolidine-1-oxyl (13a) 32
IR (neat) of 2,2,5-triethyl-5-isopropyl-3,4-bis(hydroxymethyl)-pyrrolidine-1-oxyl (13c) 32
IR (KBr) of 2,2,5-triethyl-5-tert-butyl-3,4-bis(hydroxymethyl)-pyrrolidine-1-oxyl (13b) 33
IR (KBr) of 2,5,5-triethyl-2-ethynyl-4-pyrrolidino-2,5-dihydroimidazol-1-oxyl (17) 33
IR (neat) of 2,2,5,5-tetraethyl-4-pyrrolidino-2,5-dihydroimidazol-1-oxyl (21) 33
IR (KBr) of 2,2,5-triethyl-5-ethynyl-3,4,4-trimethylimidazolidin-1-oxyl (16) 34
IR (KBr) of 2,2,5,5-tetraethyl-3,4,4-trimethylimidazolidin-1-oxyl (20) 34
IR (neat) of 2,2,5-triethyl-5-ethynylpyrrolidin-1-oxyl (15) 34
IR (neat) of 2,2,5,5-tetraethylpyrrolidin-1-oxyl (19) 35
IR (KBr) of (3S,4S,5S)-3,4-di-tert-butoxy-2,2,5-triethyl-5-ethynylpyrrolidine 1-oxyl (14) 35
IR (KBr) of (3S,4S)-3,4-di-tert-butoxy-2,2,5,5-tetraethylpyrrolidine 1-oxyl (18) 35
IR (neat) of 2,2,5-triethyl-5-(3-hydroxyprop-1-yn-1-yl)pyrrolidin-1-oxyl (23) 36
IR (KBr) of 2,2,5-triethyl-5-phenylethynyl-3,4-bis(hydroxymethyl)-pyrrolidine-1-oxyl (22a) 36
IR (KBr) of 2,2,5-triethyl-5-(3-hydroxyprop-1-yn-1-yl)-3,4-bis(hydroxymethyl)-pyrrolidine-1-oxyl (22b) 36
IR (KBr) of 2,2,5-triethyl-5-(3-hydroxy-3-methylbut-1-yn-1-yl)-3,4-bis(hydroxymethyl)-pyrrolidine-1-oxyl (22c) 37
X-ray diffraction data for compounds 37
Fig. 1SI. Structure of 8d (CCDC 2209668) 38
Fig. S2. Structure of 12a (CCDC 2209669) 38

Fig. S3. Structure of 12c (CCDC 2209670) 38
Fig. S4. Structure of 12b (CCDC 2209671) 38
Fig. S5. Structure of 13b (CCDC 2209672) 38
Fig. 6S. Structure of 14 (CCDC 2209676) 38
Fig. S7. Structure of 22a (CCDC 2209673) 39
Fig. S8. Structure of 22b (CCDC 2209674) 39
Fig. S9. Structure of 22c (CCDC 2209675) 39
Table S1. Experimental details 40
EPR 43
EPR of 2,2,5-triethyl-5-ethynyl-3,4-bis(hydroxymethyl)-pyrrolidine-1-oxyl (12a) 43
EPR of 2,2-diethyl-5-isopropyl-5-ethynyl-3,4-bis(hydroxymethyl)-pyrrolidine-1-oxyl (12c) 43
EPR of 5-tert-dutyl-2,2-diethyl-5-ethynyl-3,4-bis(hydroxymethyl)-pyrrolidine-1-oxyl (12b) 44
EPR of 2,5,5-triethyl-2-ethynyl-4-pyrrolidino-2,5-dihydroimidazol-1-oxyl (17) 44
EPR of 2,2,5-triethyl-5-ethynyl-3,4,4-trimethylimidazolidin-1-oxyl (16) 45
EPR of 2,2,5-triethyl-5-ethynylpyrrolidin-1-oxyl (15) 45
EPR of 2,2,5,5-tetraethyl-3,4-bis(hydroxymethyl)-pyrrolidine-1-oxyl (13a) 46
EPR of 2,2,5-triethyl-5-isopropyl-3,4-bis(hydroxymethyl)-pyrrolidine-1-oxyl (13c) 46
EPR of 2,2,5-triethyl-5-tert-butyl-3,4-bis(hydroxymethyl)-pyrrolidine-1-oxyl (13b) 47
EPR of 2,2,5,5-tetraethyl-4-pyrrolidino-2,5-dihydroimidazol-1-oxyl (21) 47
EPR of 2,2,5,5-tetraethyl-3,4,4-trimethylimidazolidin-1-oxyl (20) 48
EPR of 2,2,5,5-tetraethylpyrrolidin-1-oxyl (19) 48
EPR of (3S,4S,5S)-3,4-di-tert-butoxy-2,2,5-triethyl-5-ethynylpyrrolidine 1-oxyl (14) 49
EPR of (3S,4S)-3,4-di-tert-butoxy-2,2,5,5-tetraethylpyrrolidine 1-oxyl (18) 49
EPR of 2,2,5-triethyl-5-(3-hydroxyprop-1-yn-1-yl)pyrrolidin-1-oxyl (23) 50
EPR of 2,2,5-triethyl-5-phenylethynyl-3,4-bis(hydroxymethyl)-pyrrolidine-1-oxyl (22a) 50
EPR of 2,2,5-triethyl-5-(3-hydroxyprop-1-yn-1-yl)-3,4-bis(hydroxymethyl)-pyrrolidine-1-oxyl (22b) 51
EPR of 2,2,5-triethyl-5-(3-hydroxy-3-methylbut-1-yn-1-yl)-3,4-bis(hydroxymethyl)-pyrrolidine-1-oxyl (22c) 51
HPLC 52
HPLC of (3S,4S,5S)-3,4-di-tert-butoxy-2,2,5-triethyl-5-ethynylpyrrolidine 1-oxyl (14) 52

Spectra

¹H NMR of 2,2-diethyl-5-isopropyl-3,4-bis(methoxycarbonyl)pyrrolidine (6c)

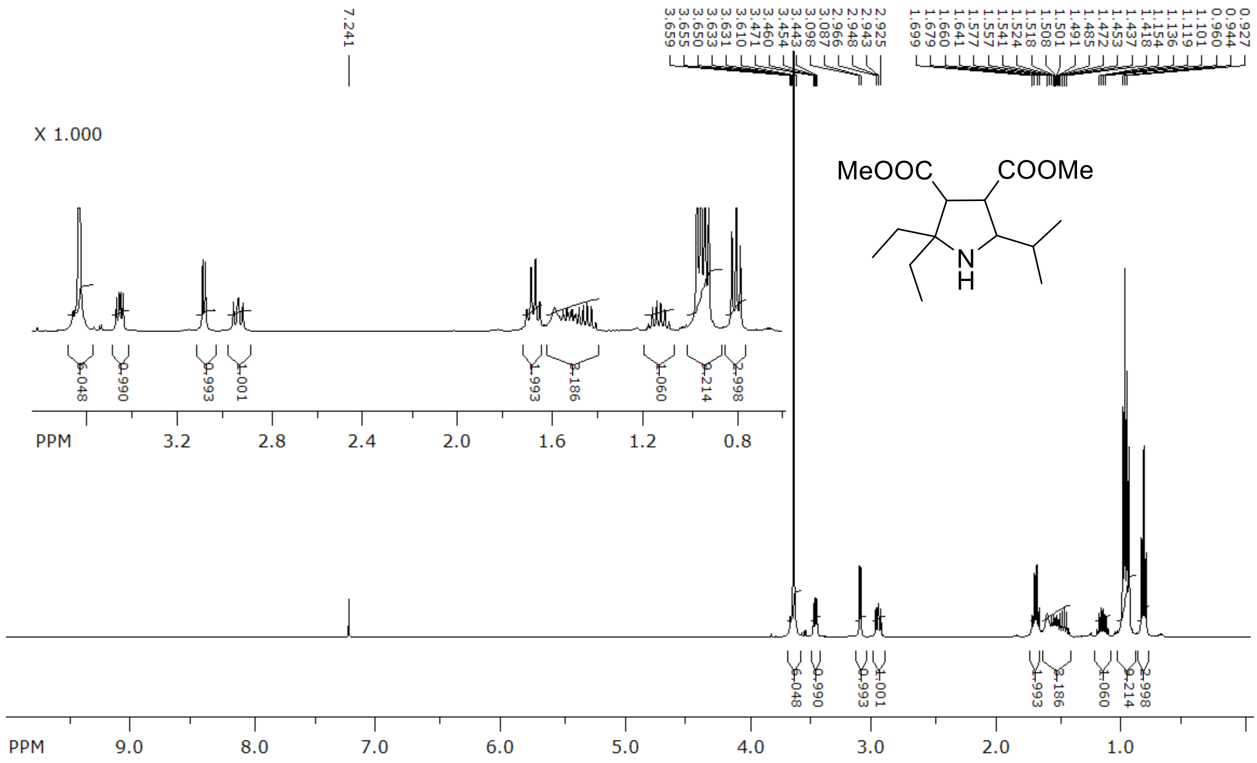

¹³C{¹H} NMR of 2,2-diethyl-5-isopropyl-3,4-bis(methoxycarbonyl)pyrrolidine (6c)

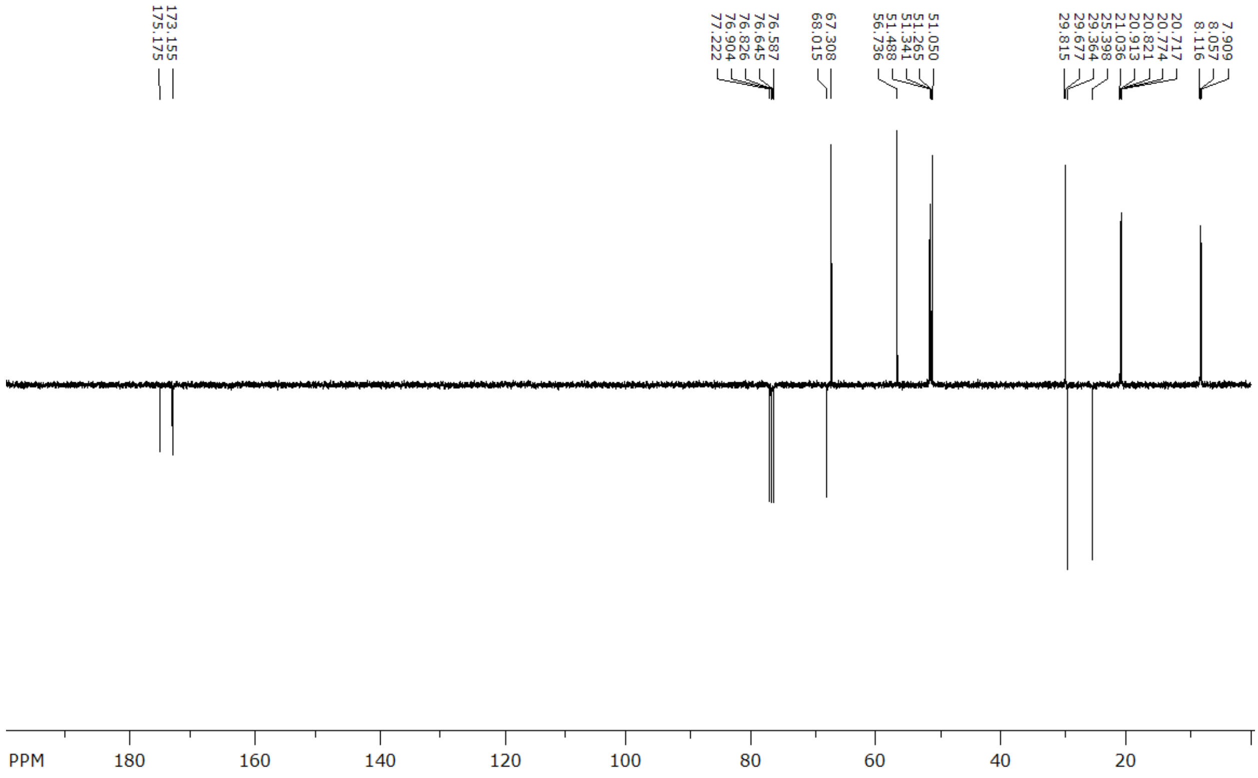

^{1}H NMR of 2,2-diethyl-5-isopropyl-3,4-bis(methoxycarbonyl)pyrrolidine (7c)

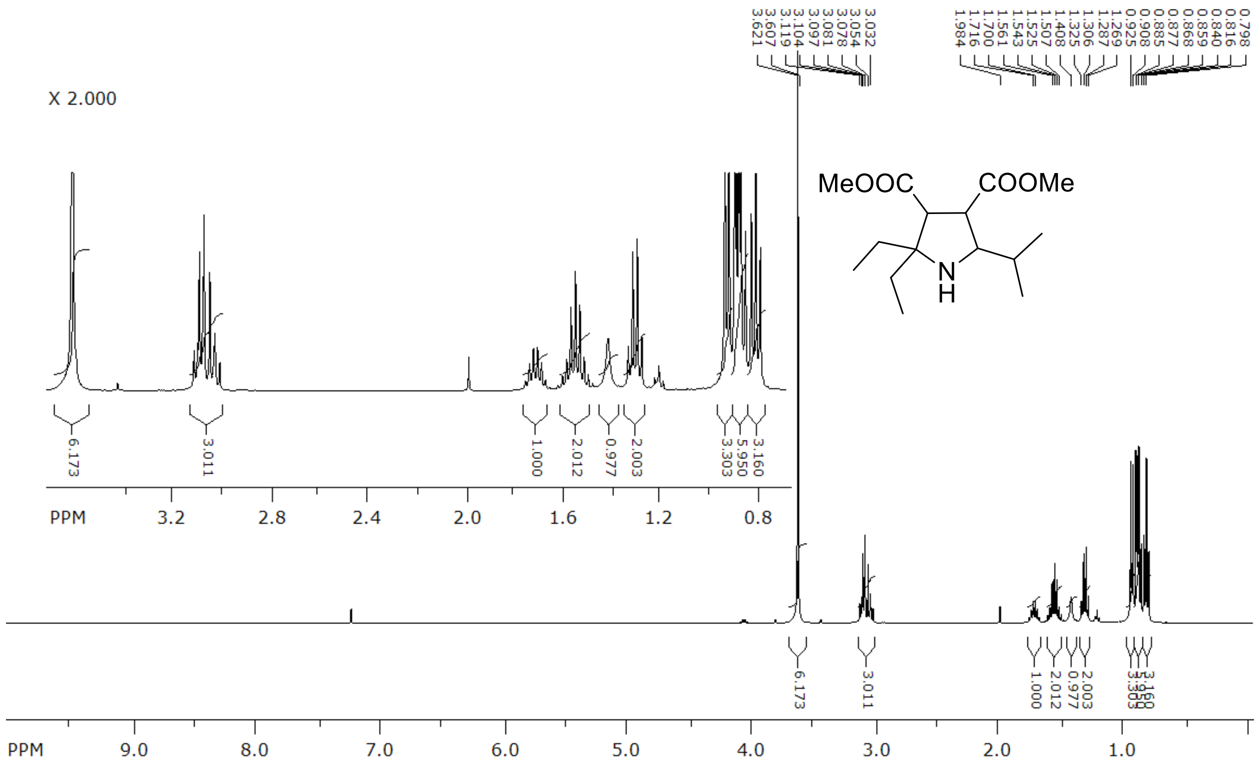

^{13}C{^{1}H} NMR of 2,2-diethyl-5-isopropyl-3,4-bis(methoxycarbonyl)pyrrolidine (7c)

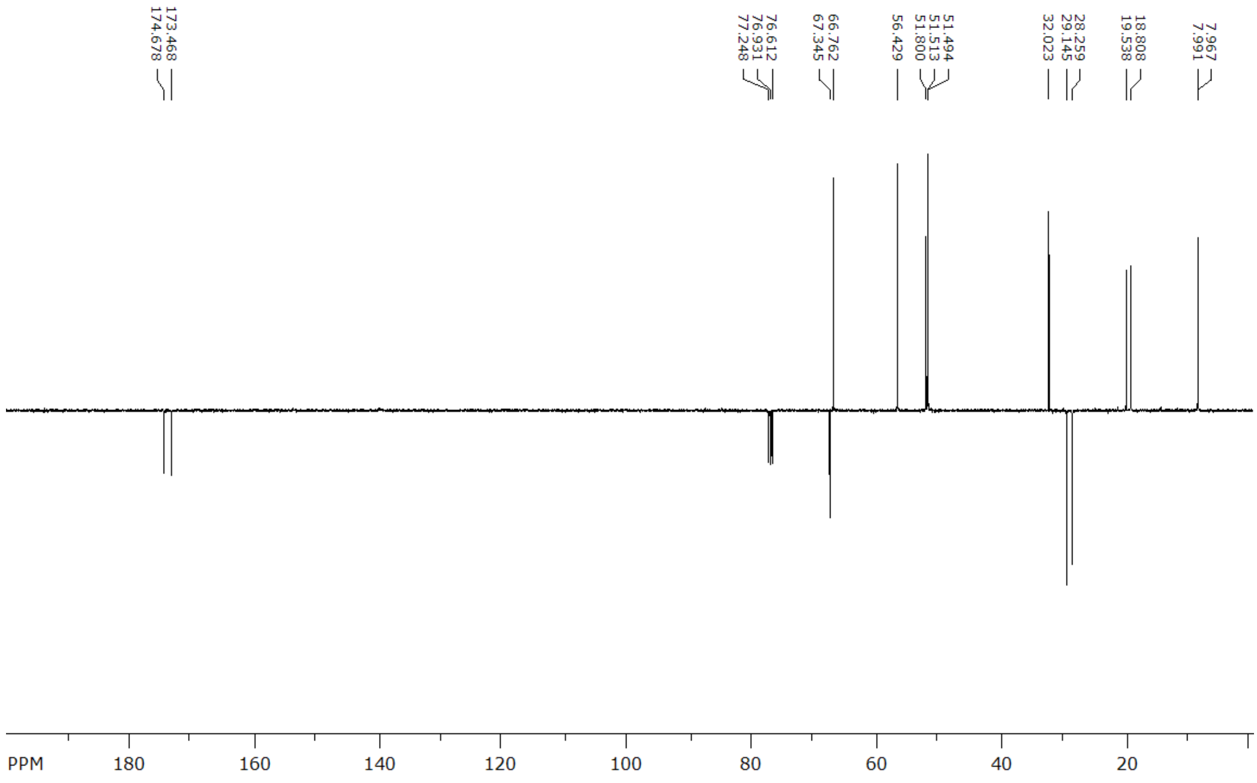

^{1}H NMR of 2,2,5-triisopropyl-3,4-bis(methoxycarbonyl)pyrrolidine (6d)

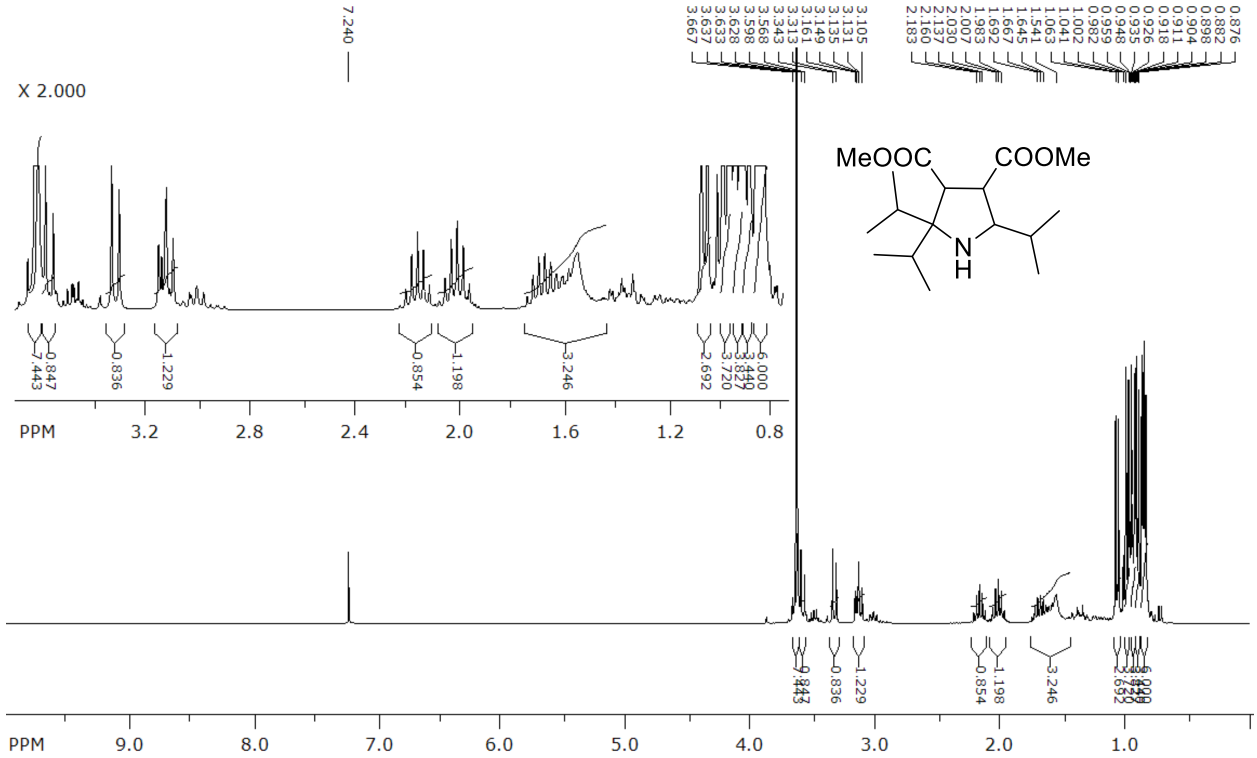

^{13}C{^{1}H} NMR of 2,2,5-triisopropyl-3,4-bis(methoxycarbonyl)pyrrolidine (6d)

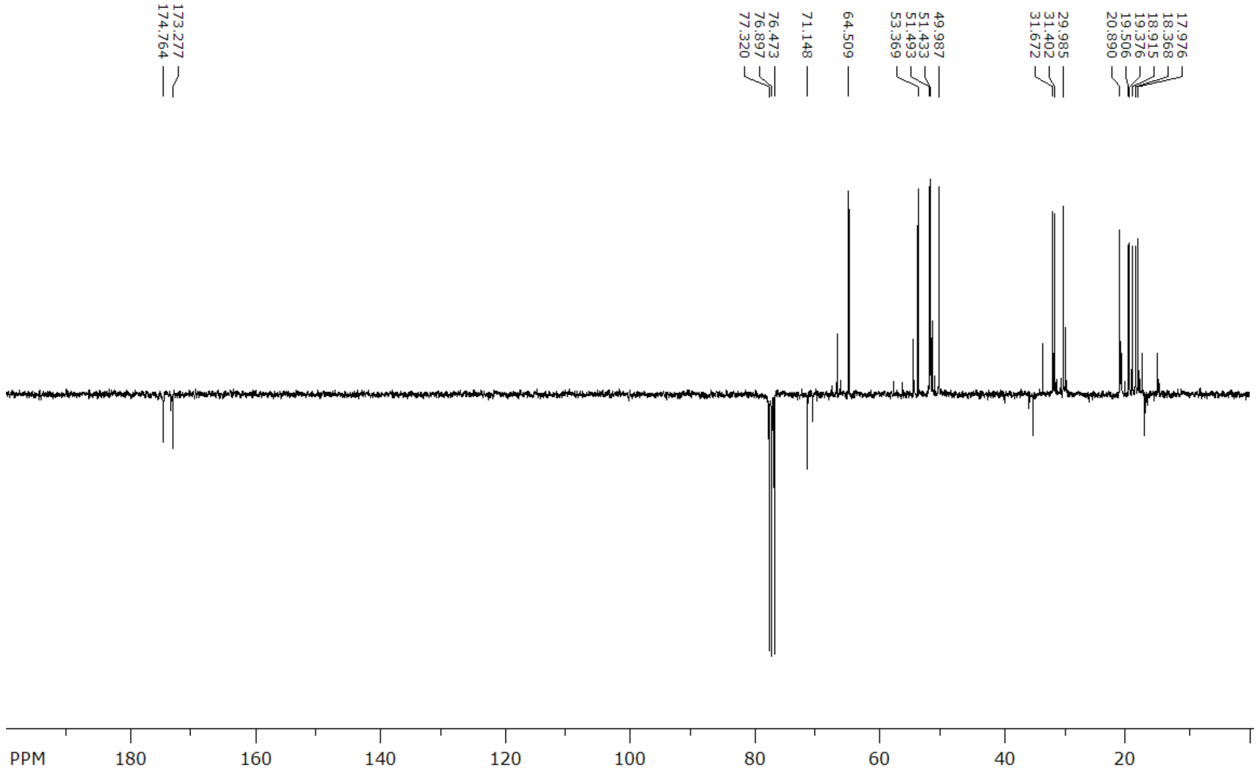

^{1}H NMR of 2,2,5-triisopropyl-3,4-bis(methoxycarbonyl)pyrrolidine (7d)

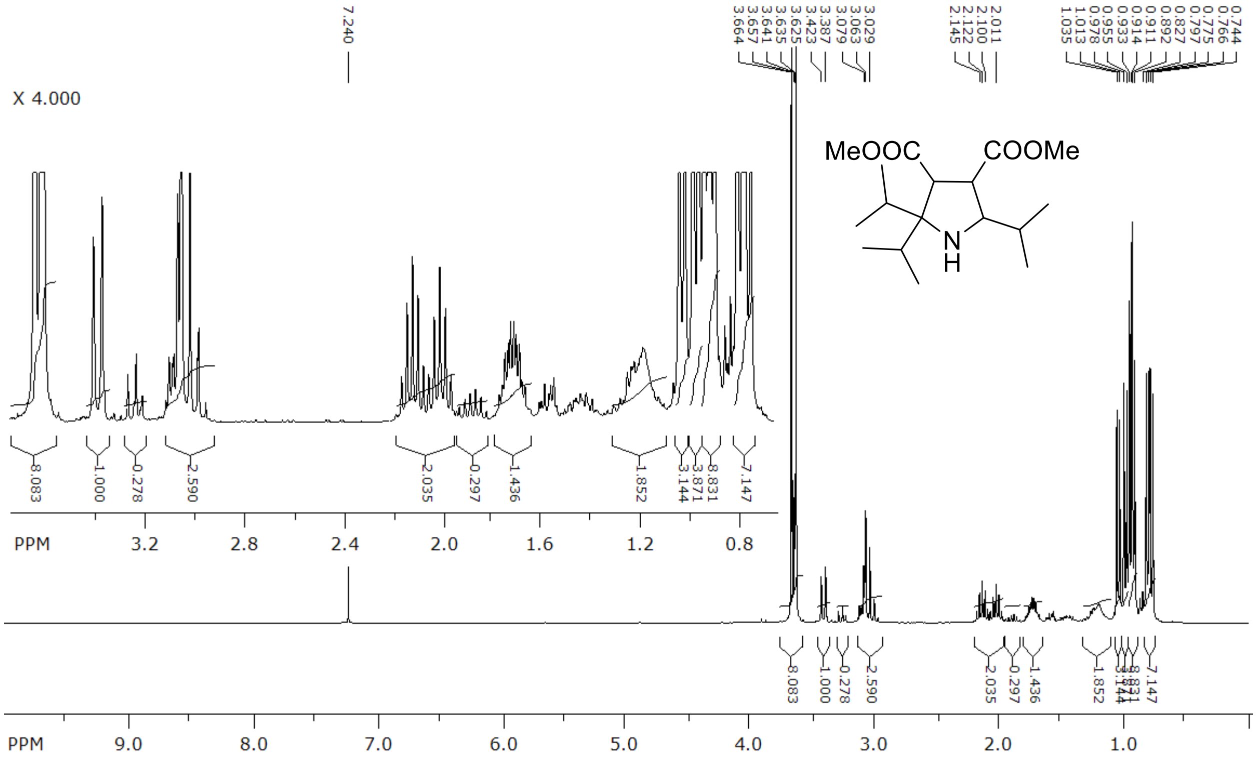

$^{13}C\{^{1}H\}$ NMR of 2,2,5-triisopropyl-3,4-bis(methoxycarbonyl)pyrrolidine (7d)

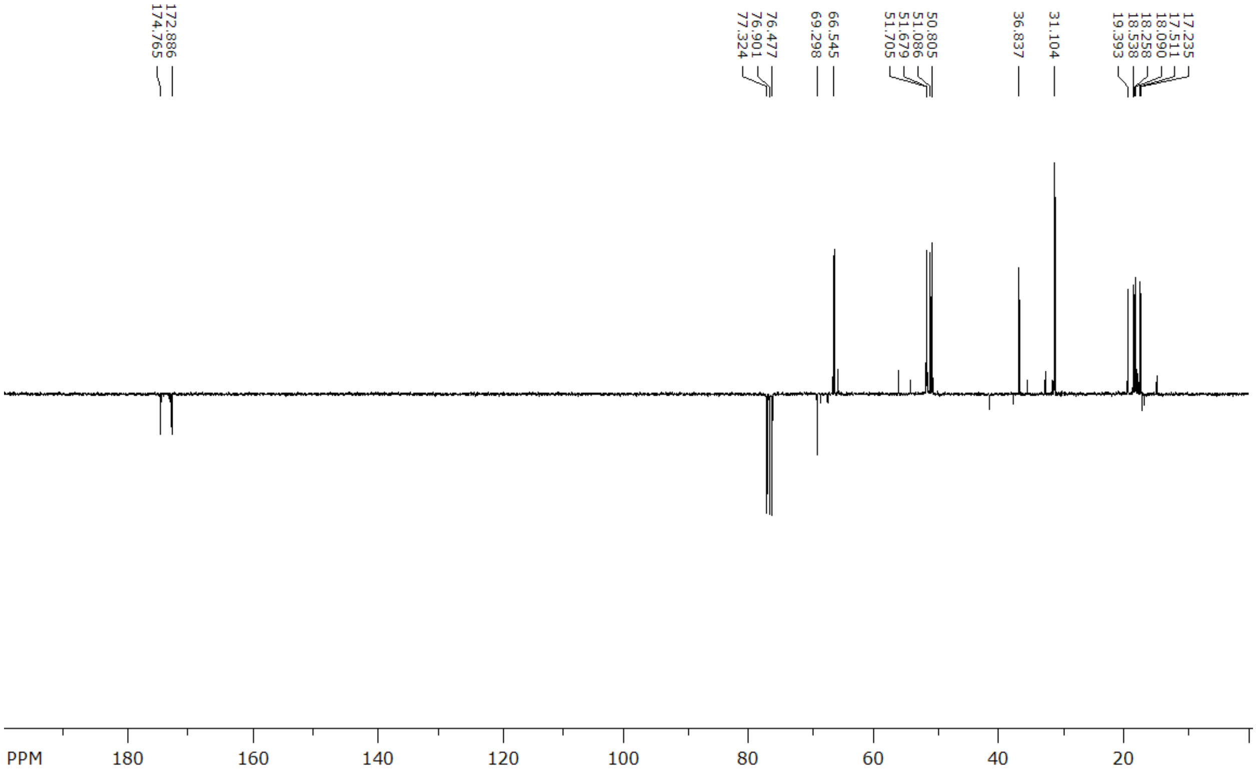

^{1}H NMR of 2,2-diethyl-5-isopropyl-3,4-bis(hydroxymethyl)-3,4-dihydro-2H-pyrrole 1-oxide (8c)

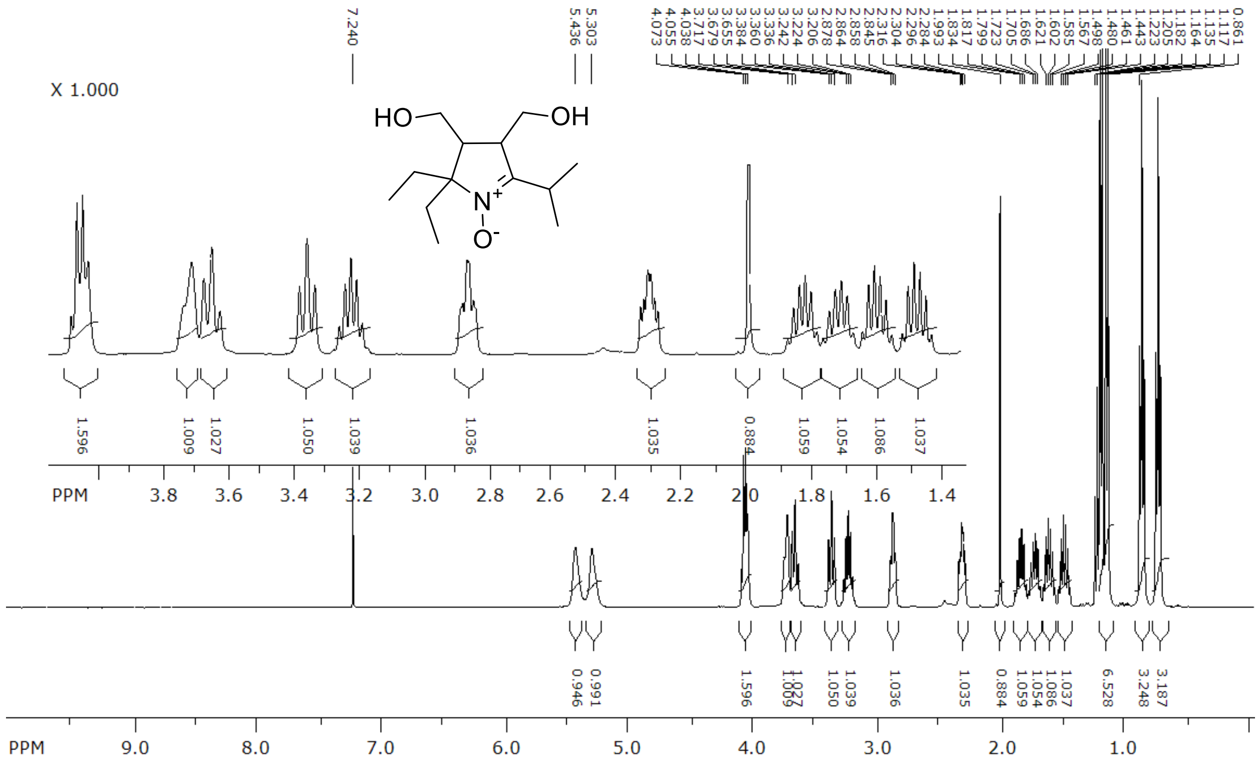

^{13}C{^{1}H} NMR of 2,2-diethyl-5-isopropyl-3,4-bis(hydroxymethyl)-3,4-dihydro-2H-pyrrole 1-oxide (8c)

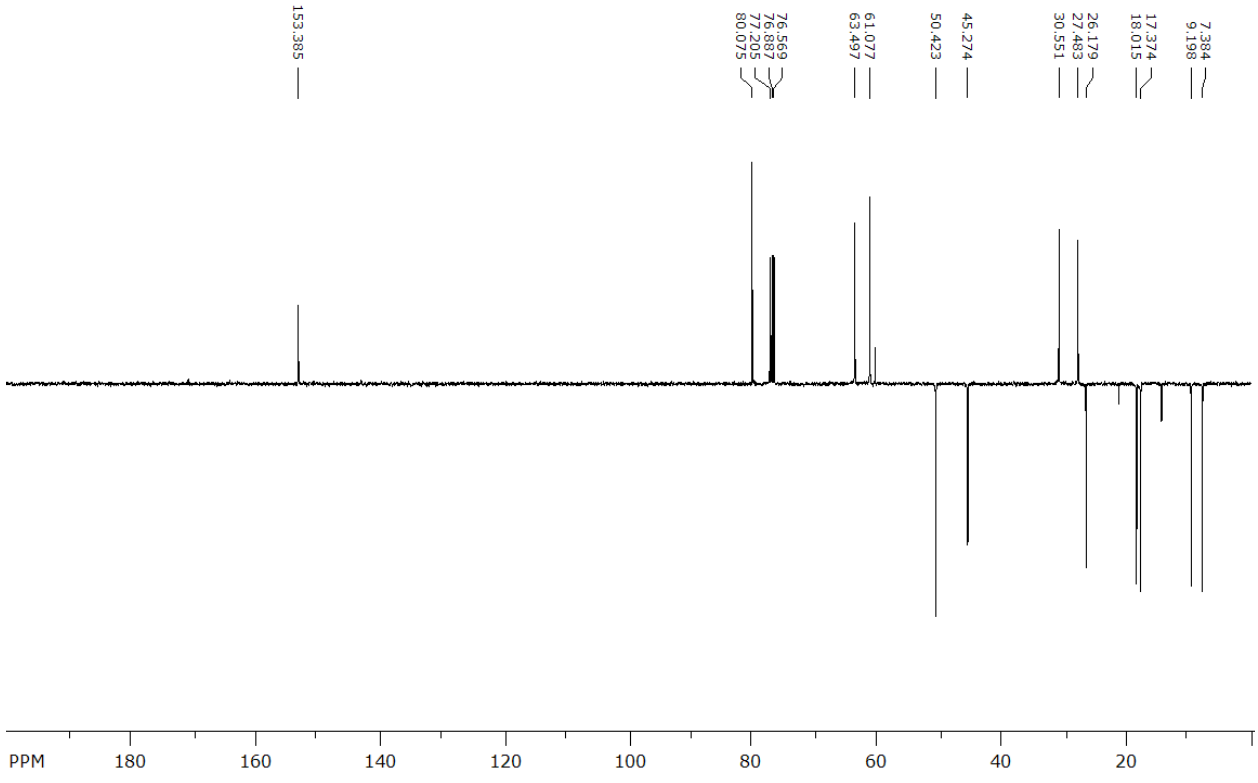

^{1}H NMR of 2,2,5-triisopropyl-3,4-bis(hydroxymethyl)-3,4-dihydro-2H-pyrrole 1-oxide (8d)

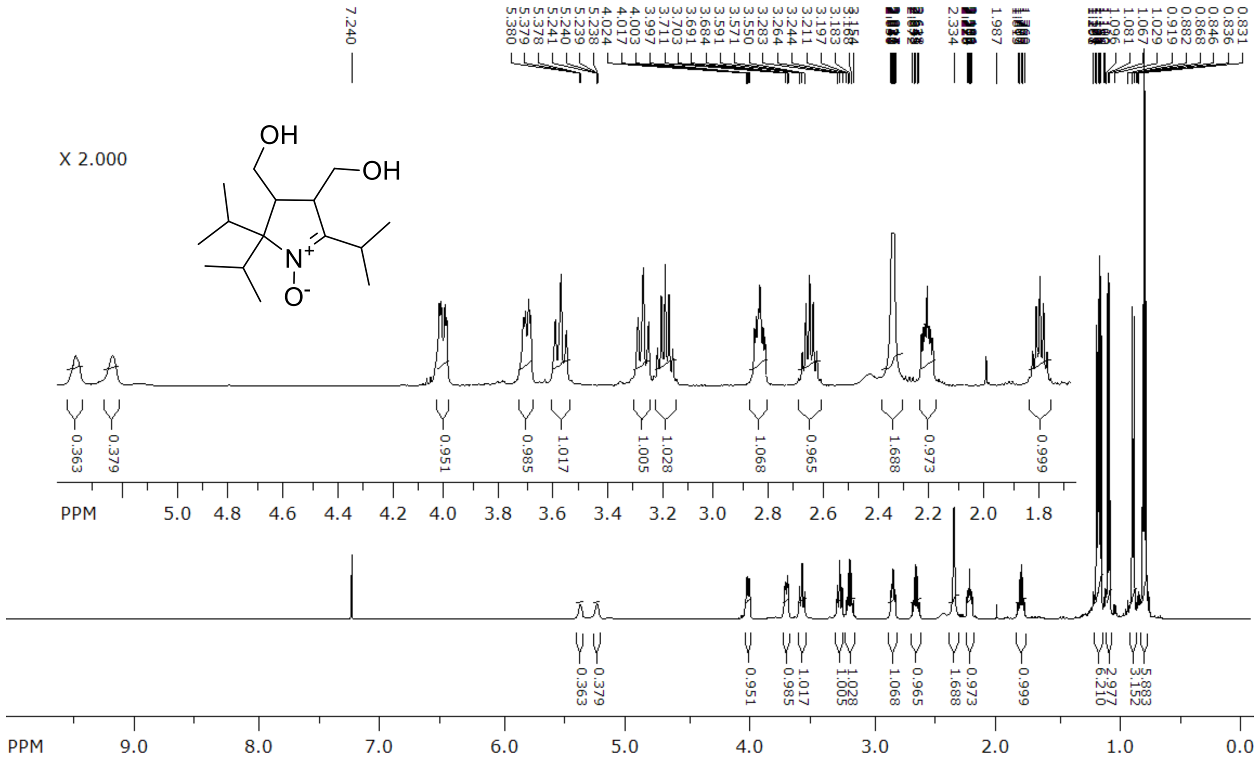

$^{13}C\{^{1}H\}$ NMR of 2,2,5-triisopropyl-3,4-bis(hydroxymethyl)-3,4-dihydro-2H-pyrrole 1-oxide (8d)

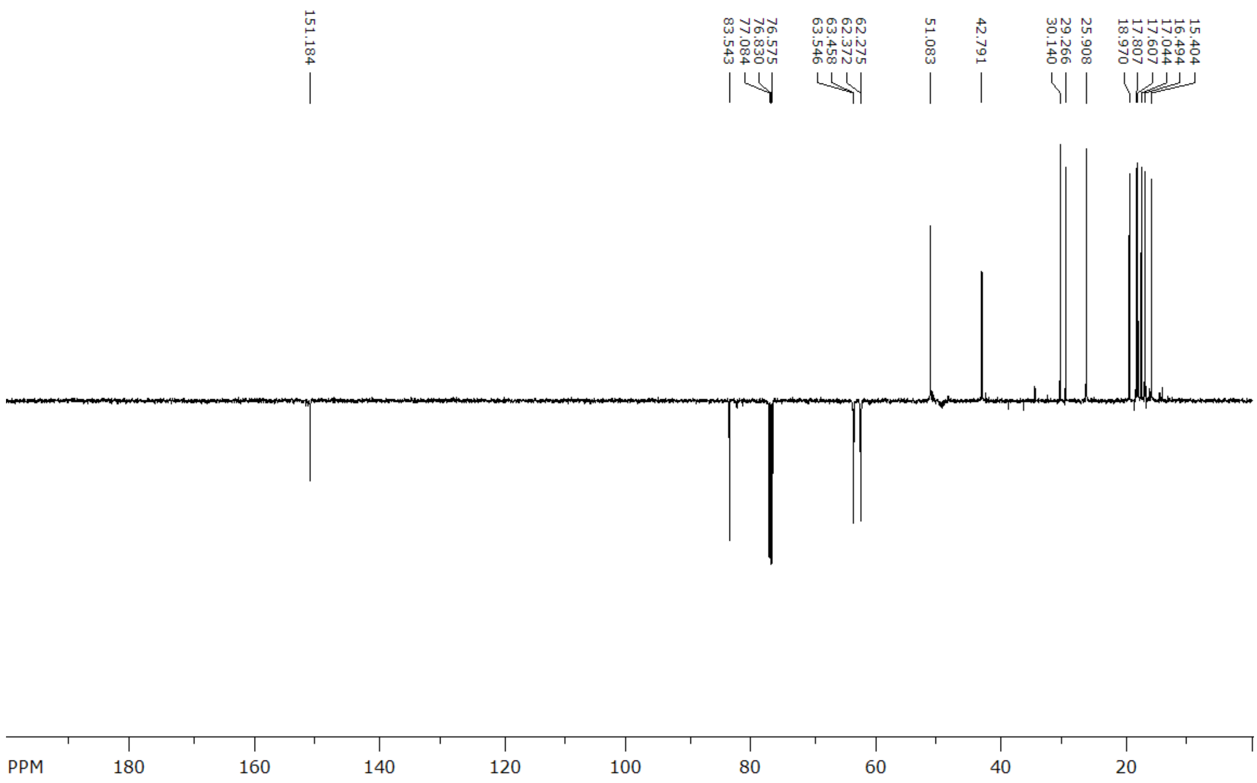

^{1}H NMR of 2,2,5-triethyl-3,4-bis(((2-methoxypropan-2-yl)oxy)methyl)-3,4-dihydro-2H-pyrrole 1-oxide (1a)

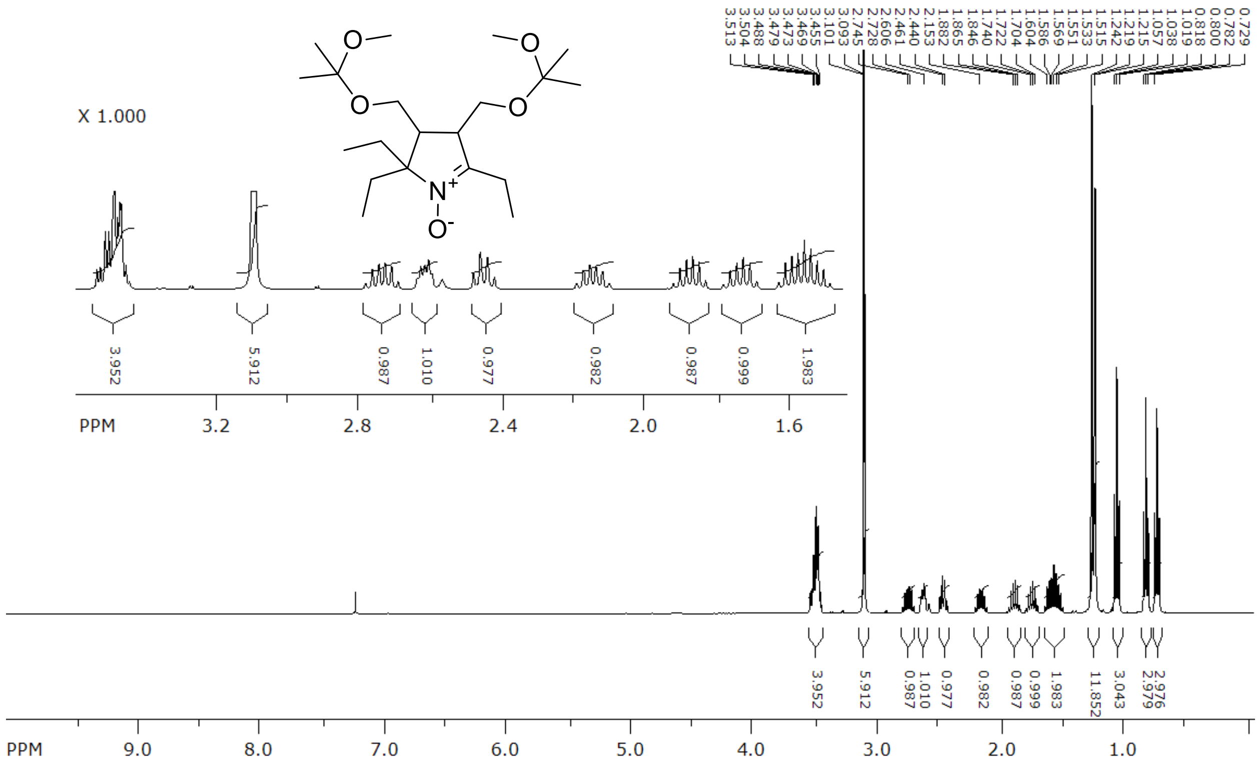

^{13}C{^{1}H} NMR of 2,2,5-triethyl-3,4-bis(((2-methoxypropan-2-yl)oxy)methyl)-3,4-dihydro-2H-pyrrole 1-oxide (1a)

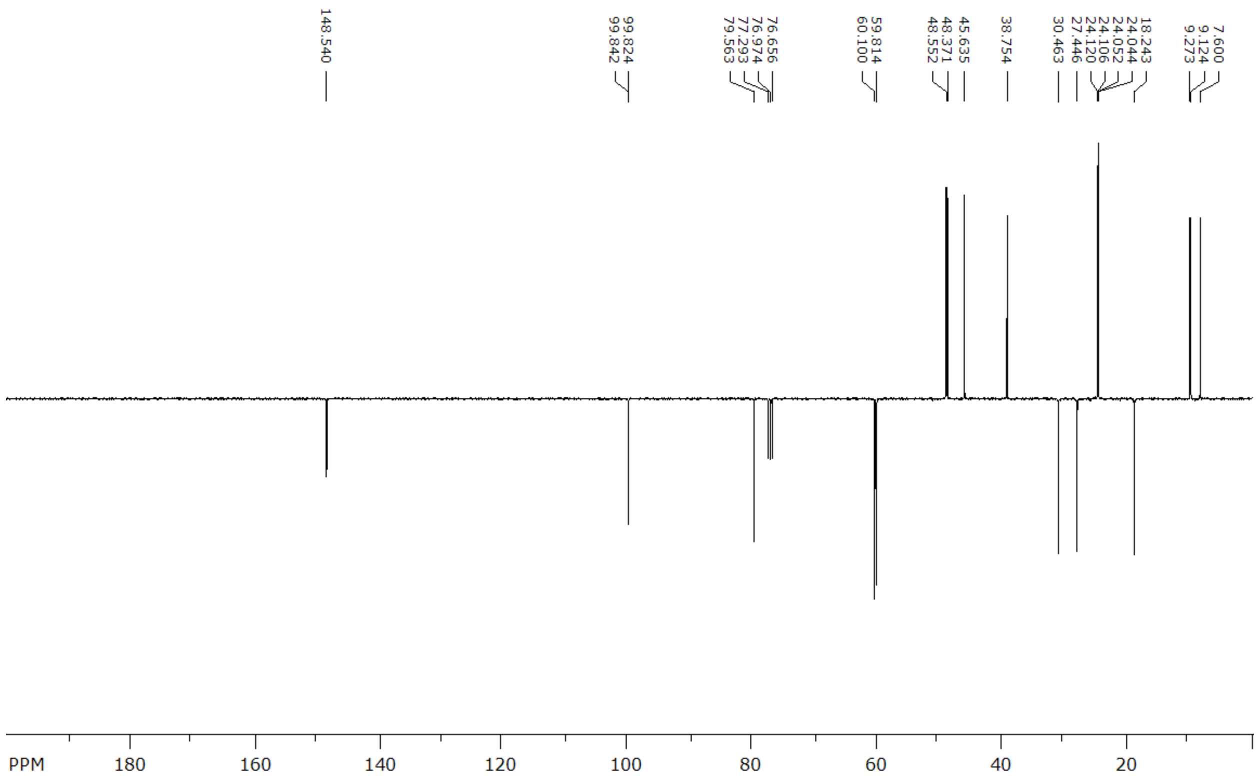

^{1}H NMR of 2,2-diethyl-5-isopropyl-3,4-bis(((2-methoxypropan-2-yl)oxy)methyl)-3,4-dihydro-2H-pyrrole 1-oxide (1c)

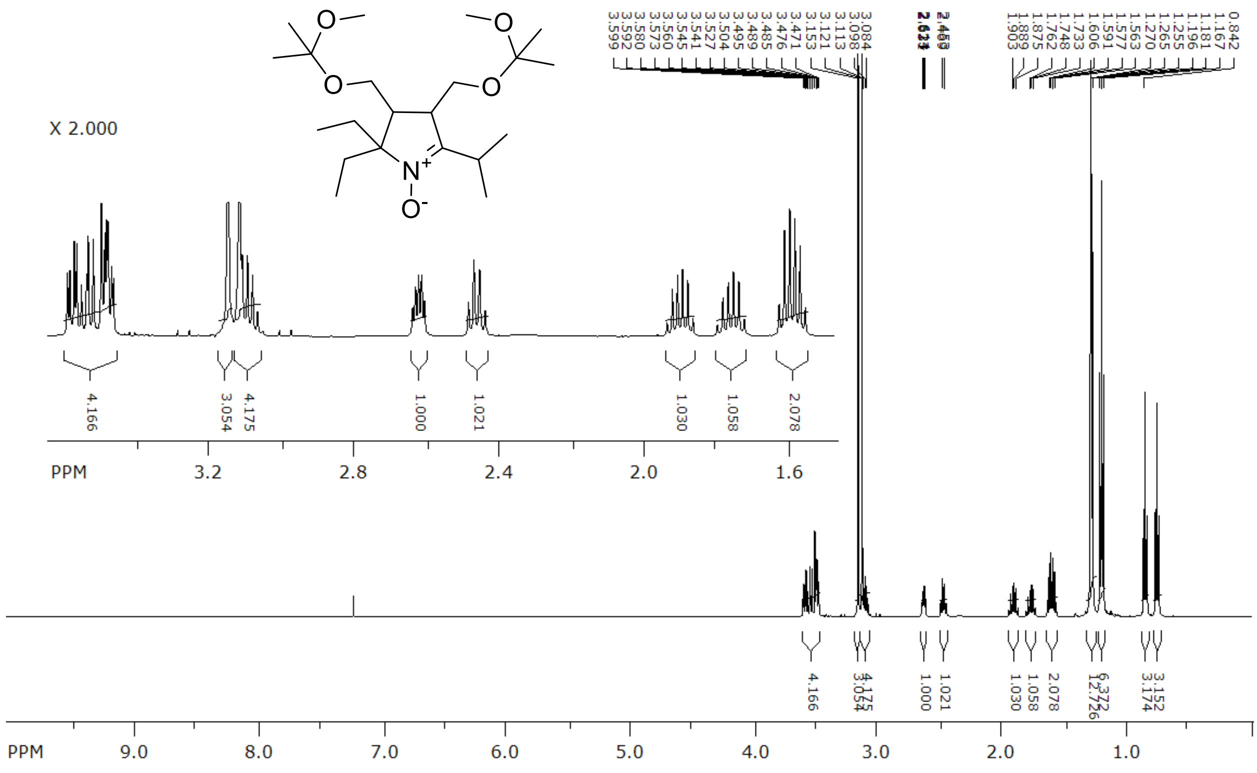

$^{13}C\{^{1}H\}$ NMR of 2,2-diethyl-5-isopropyl-3,4-bis(((2-methoxypropan-2-yl)oxy)methyl)-3,4-dihydro-2H-pyrrole 1-oxide (1c)

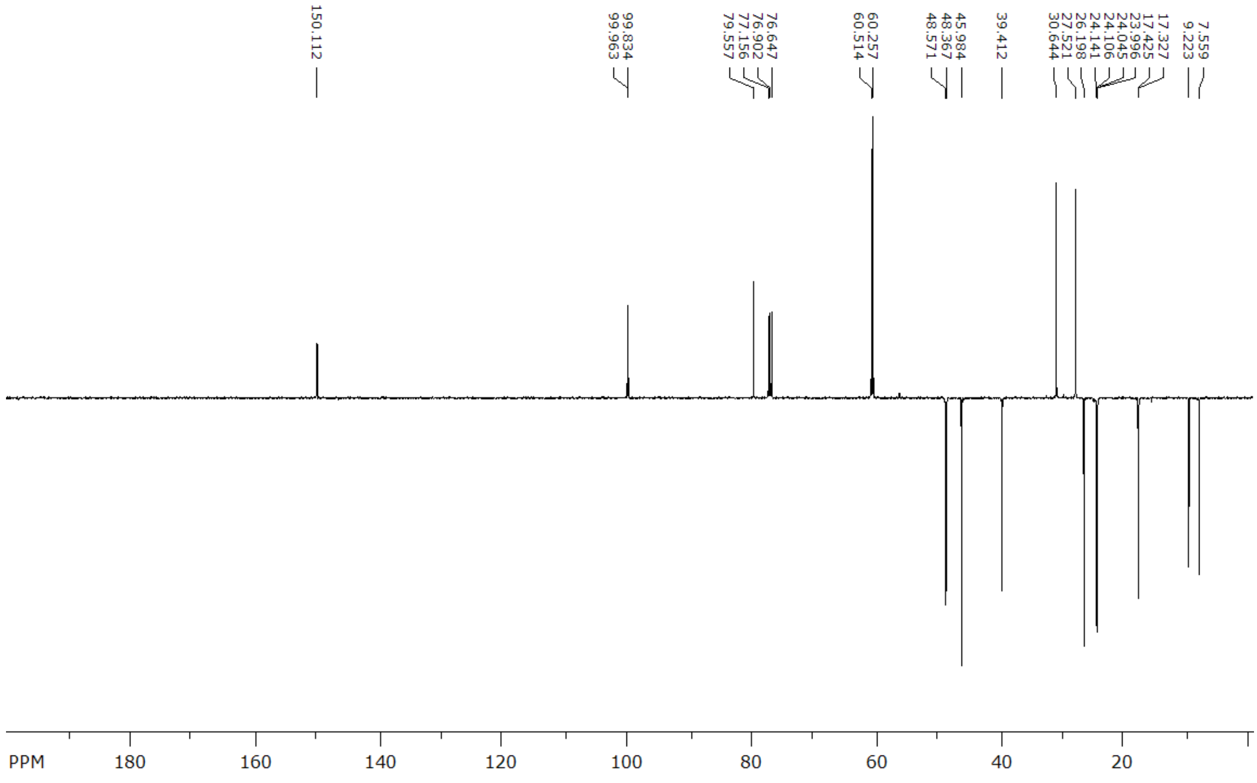

^{1}H NMR of 5-tert-butyl-2,2-diethyl-3,4-bis(((2-methoxypropan-2-yl)oxy)methyl)-3,4-dihydro-2H-pyrrole 1-oxide (1b)

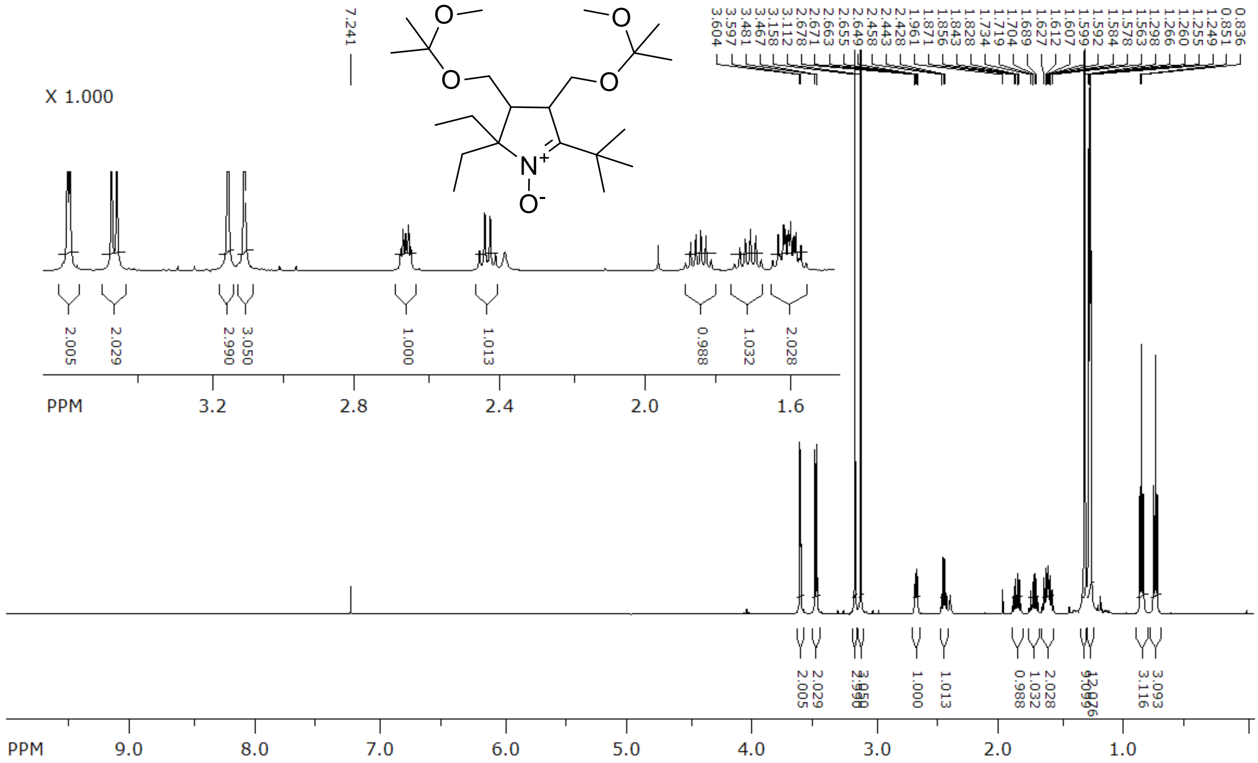

^{13}C{^{1}H} NMR of 5-tert-butyl-2,2-diethyl-3,4-bis(((2-methoxypropan-2-yl)oxy)methyl)-3,4-dihydro-2H-pyrrole 1-oxide (1b)

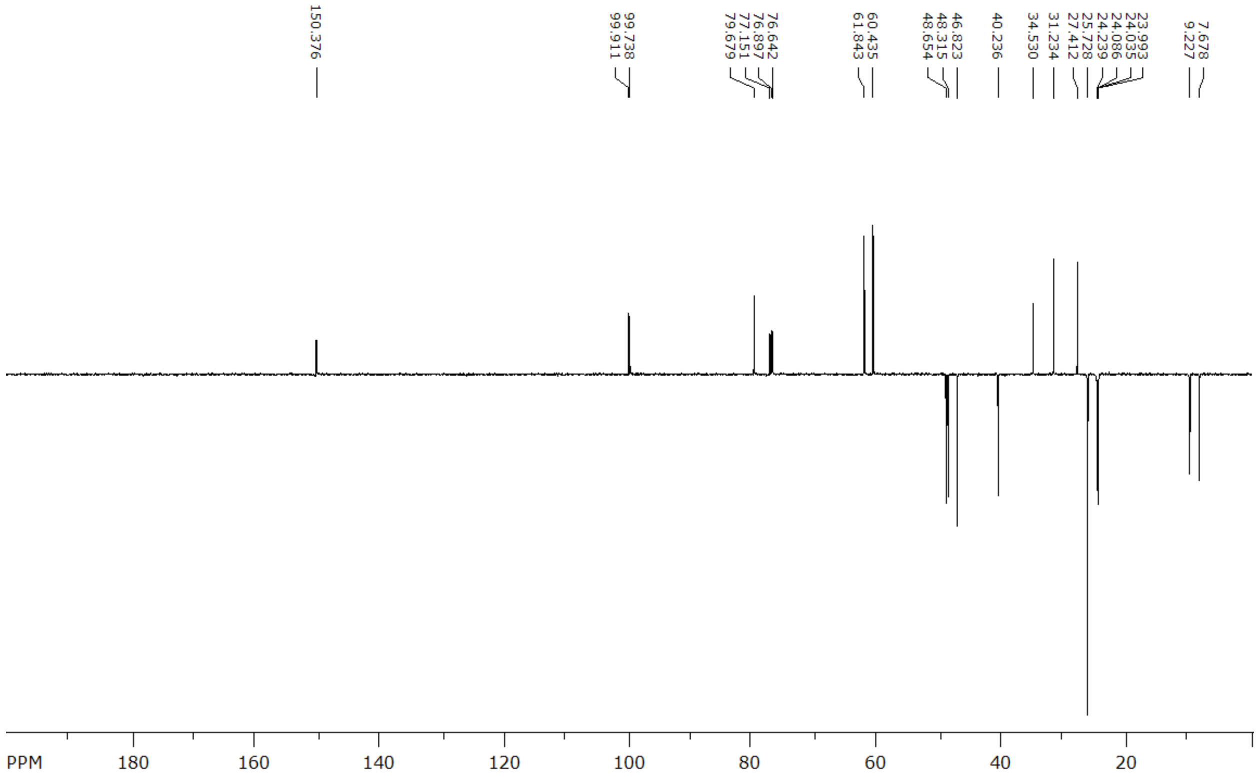

^{1}H NMR of 2,2,5-triisopropyl-3,4-bis(((2-methoxypropan-2-yl)oxy)methyl)-3,4-dihydro-2H-pyrrole 1-oxide (1d)

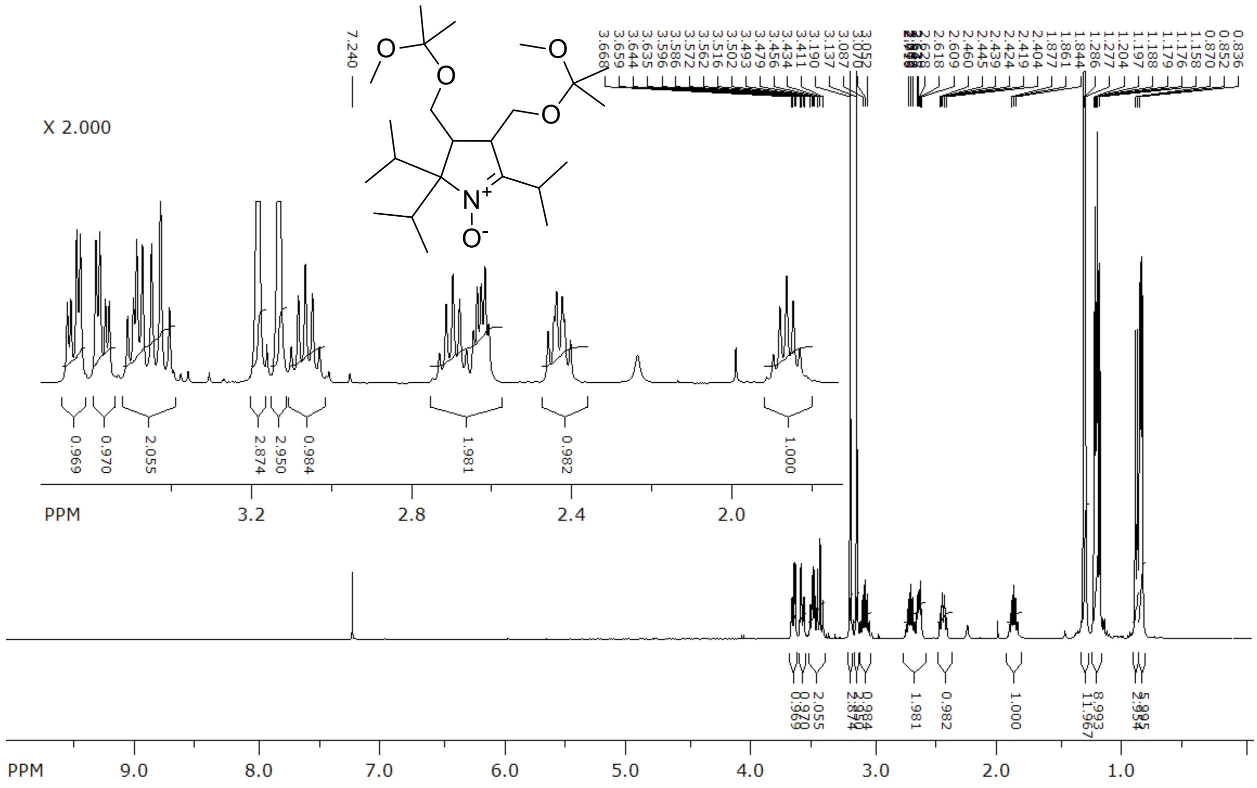

^{13}C{^{1}H} NMR of 2,2,5-triisopropyl-3,4-bis(((2-methoxypropan-2-yl)oxy)methyl)-3,4-dihydro-2H-pyrrole 1-oxide (1d)

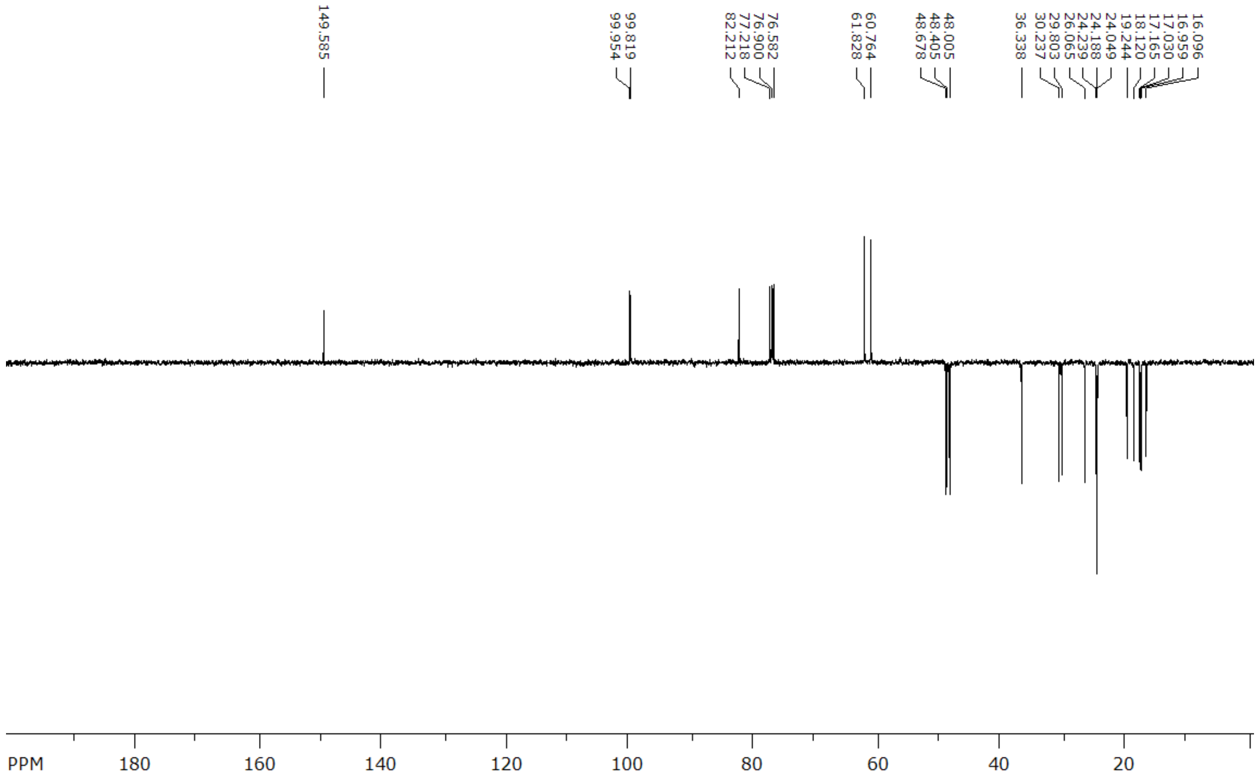

^{1}H NMR of 2,2,4-triethyl-5,5-dimethyl-2,5-dihydroimidazole 3-oxide (11)

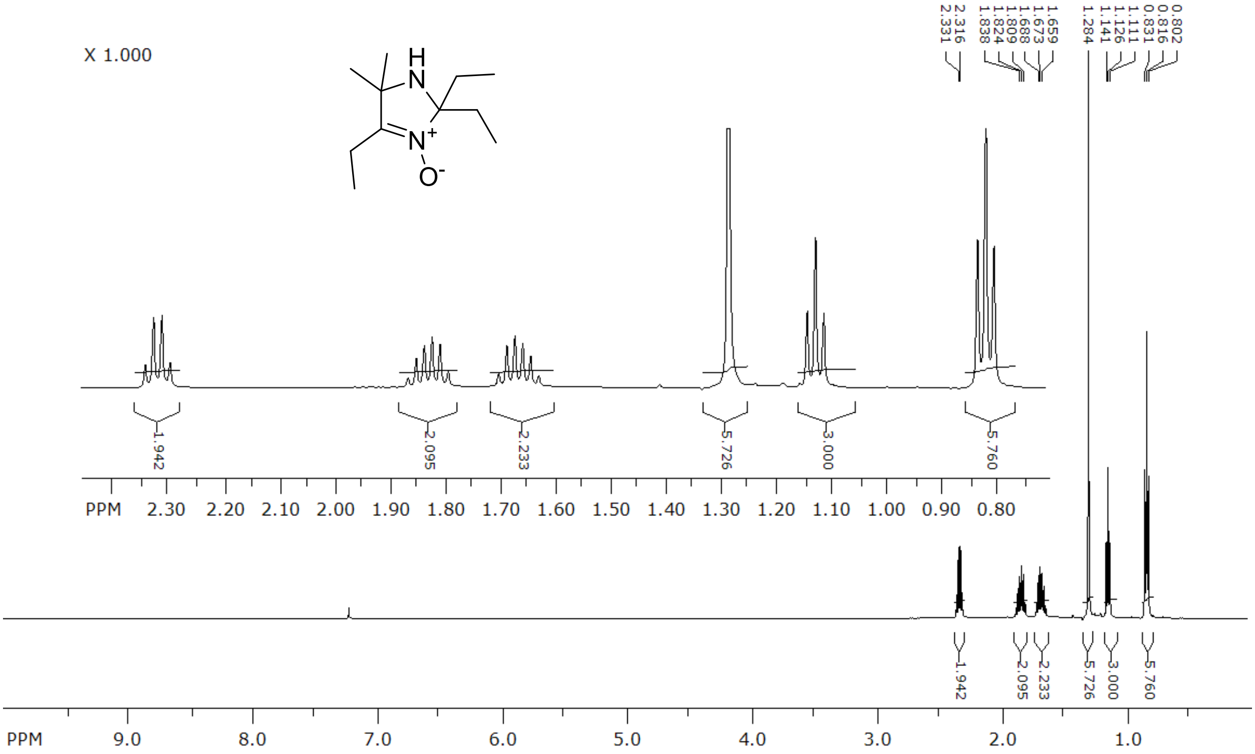

^{13}C{^{1}H} NMR of 2,2,4-triethyl-5,5-dimethyl-2,5-dihydroimidazole 3-oxide (11)

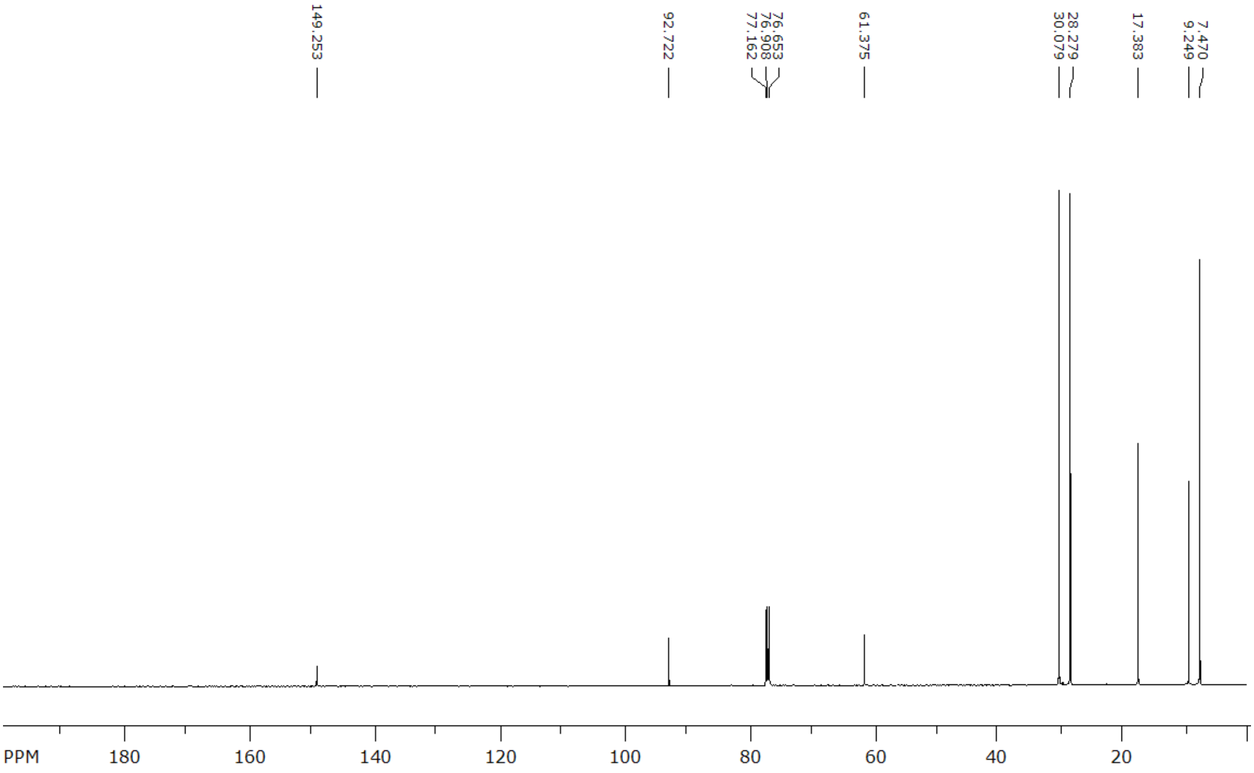

^{1}H NMR of 2,2,4-triethyl-1,5,5-trimethyl-2,5-dihydroimidazole 3-oxide (4)

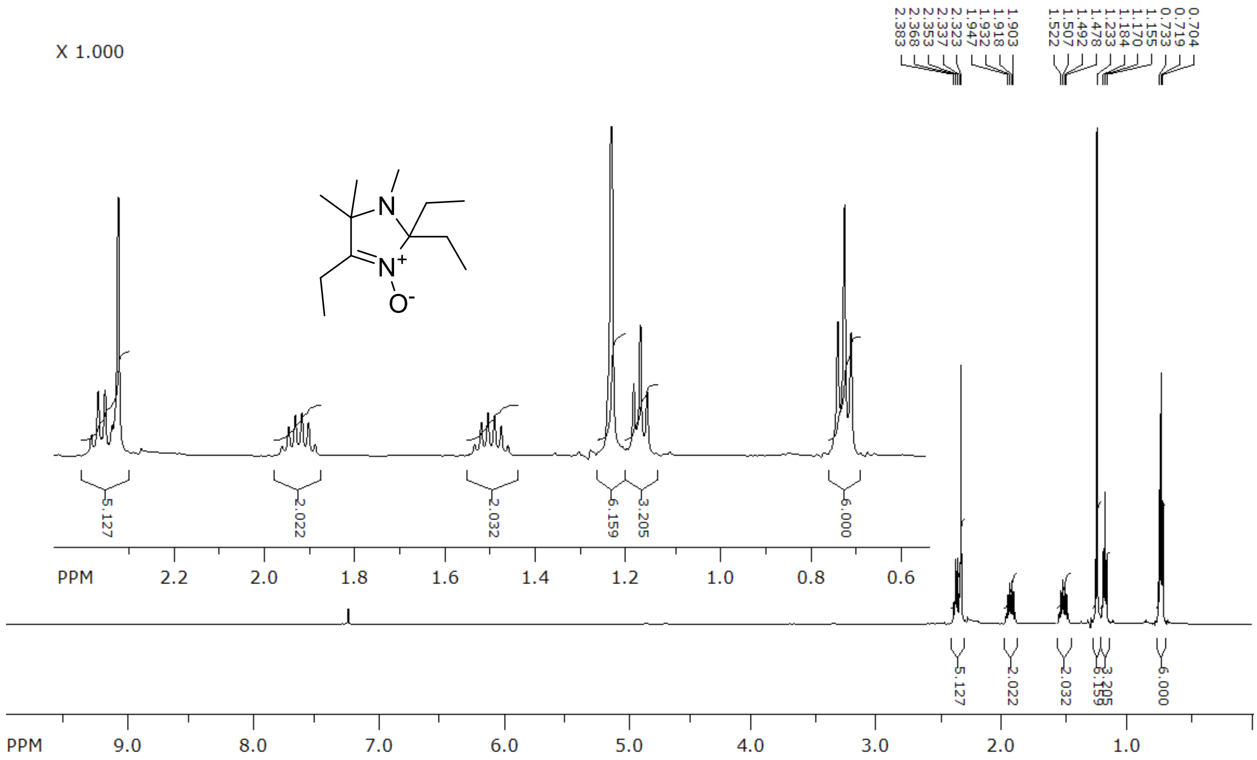

^{13}C{^{1}H} NMR of 2,2,4-triethyl-1,5,5-trimethyl-2,5-dihydroimidazole 3-oxide (4)

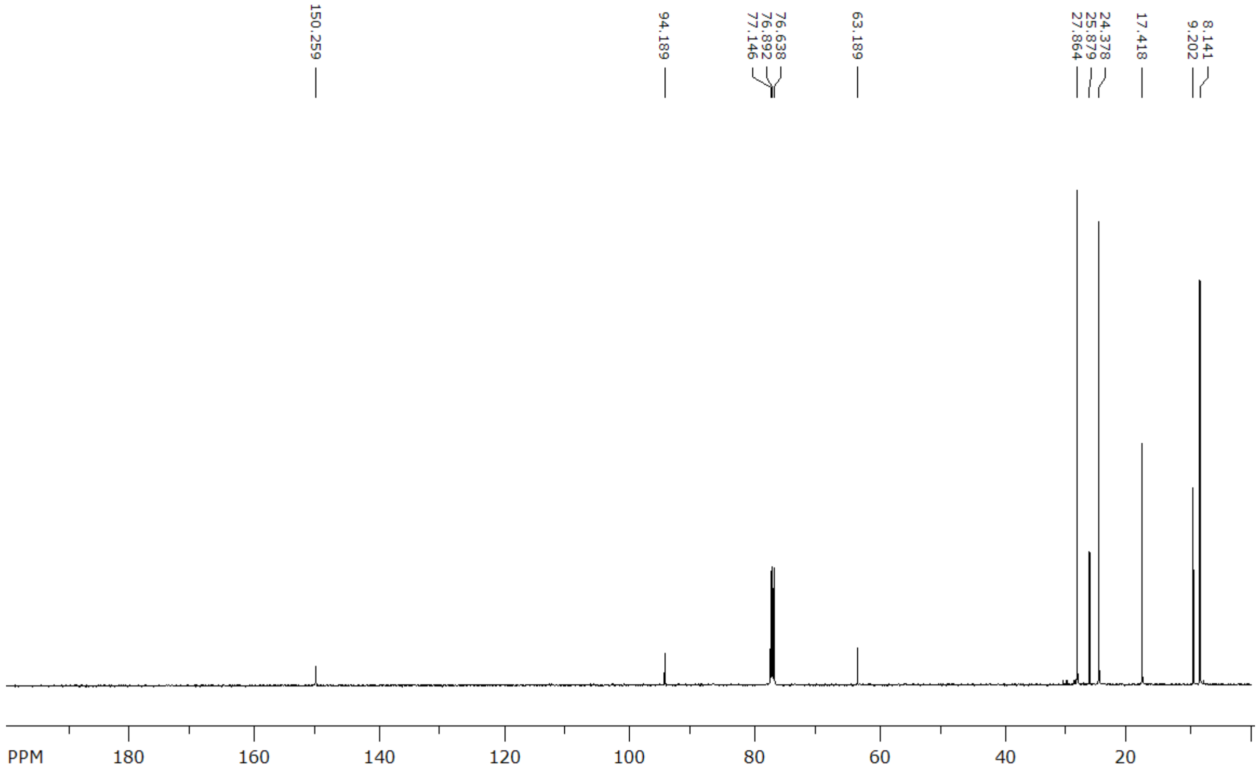

^{1}H NMR of (3S,4S)-3,4-di-tert-butoxy-2,2,5-triethyl-3,4-dihydro-2H-pyrrole 1-oxide (2)

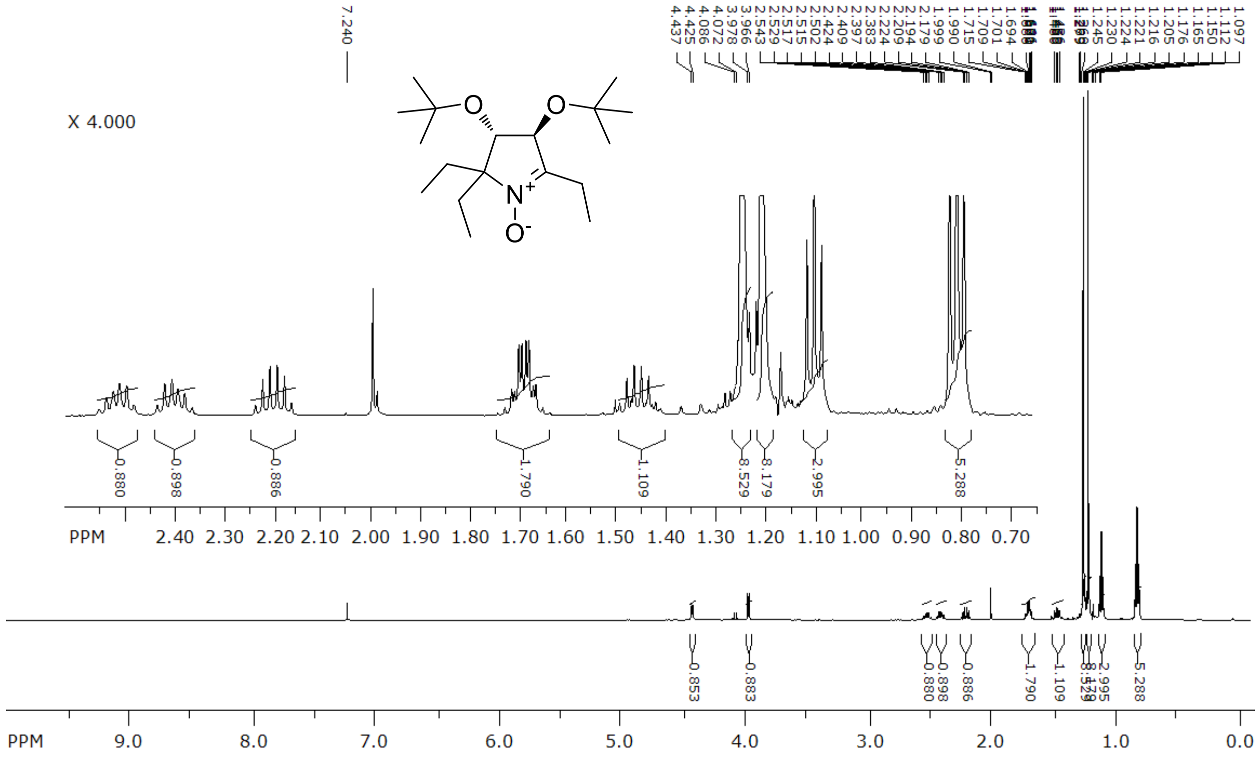

^{13}C{^{1}H} NMR of (3S,4S)-3,4-di-tert-butoxy-2,2,5-triethyl-3,4-dihydro-2H-pyrrole 1-oxide (2)

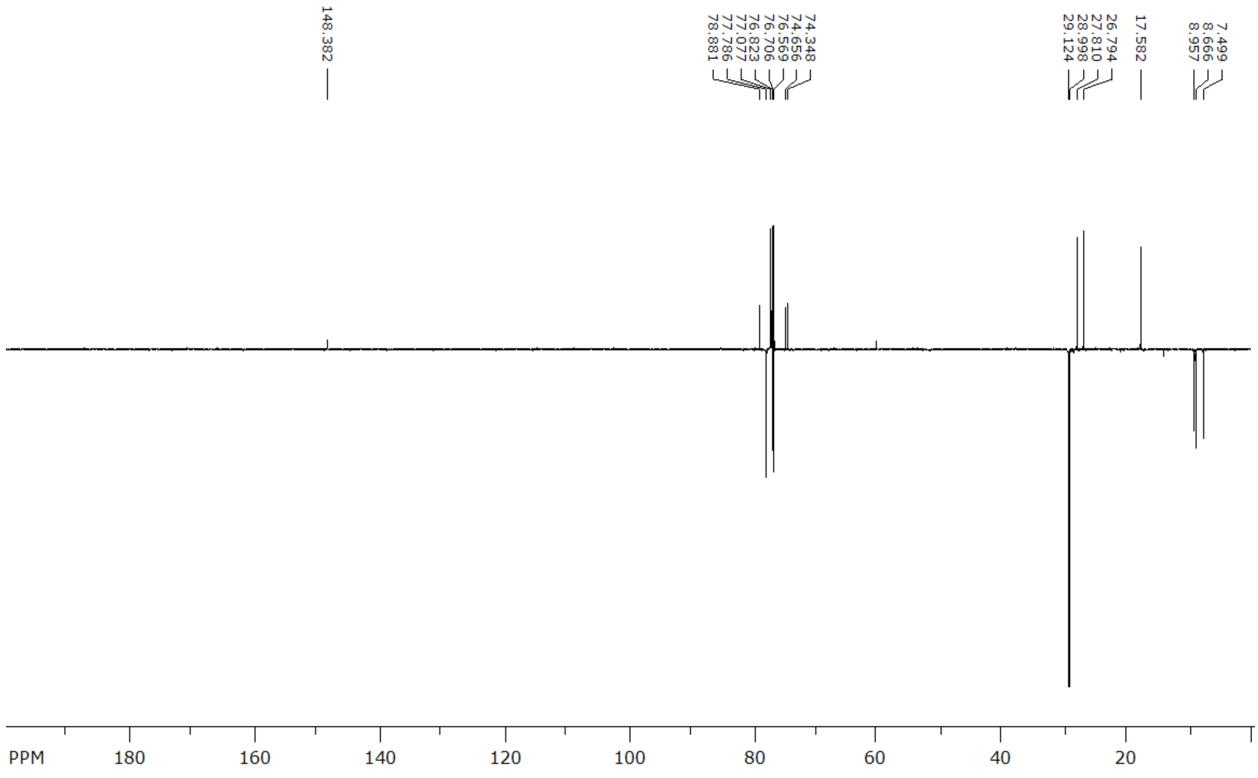

^{1}H NMR of 2,2,5-triethyl-5-ethynyl-3,4-bis(hydroxymethyl)-pyrrolidine-1-oxyl (12a)

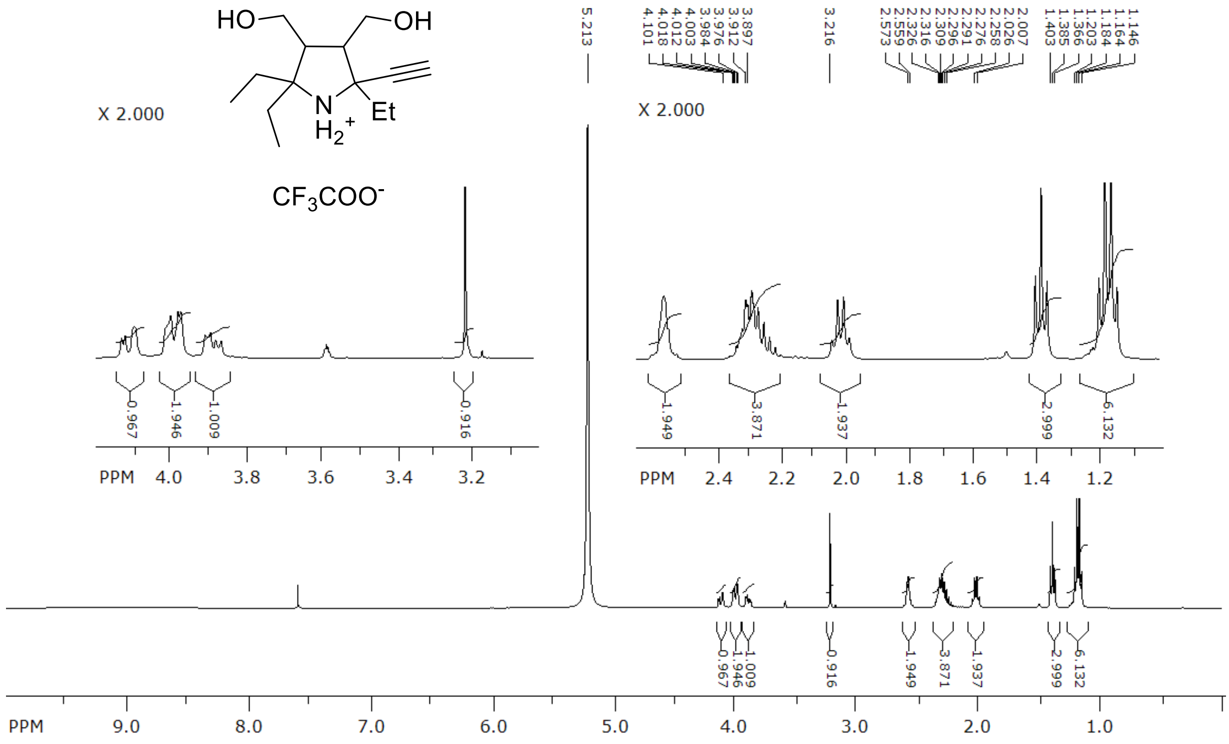

^{1}H NMR of 2,2-diethyl-5-isopropyl-5-ethynyl-3,4-bis(hydroxymethyl)-pyrrolidine-1-oxyl (12c)

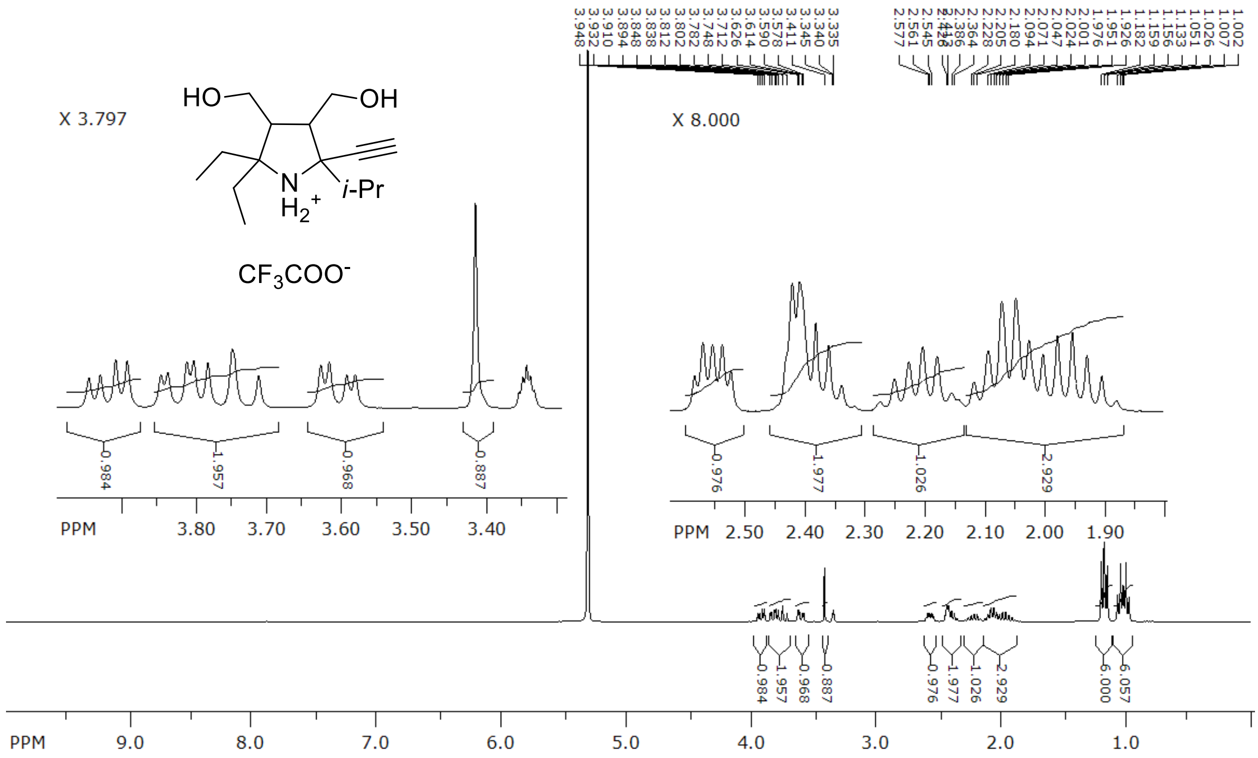

^{1}H NMR of 5-tert-dutyl-2,2-diethyl-5-ethynyl-3,4-bis(hydroxymethyl)-pyrrolidine-1-oxyl (12b)

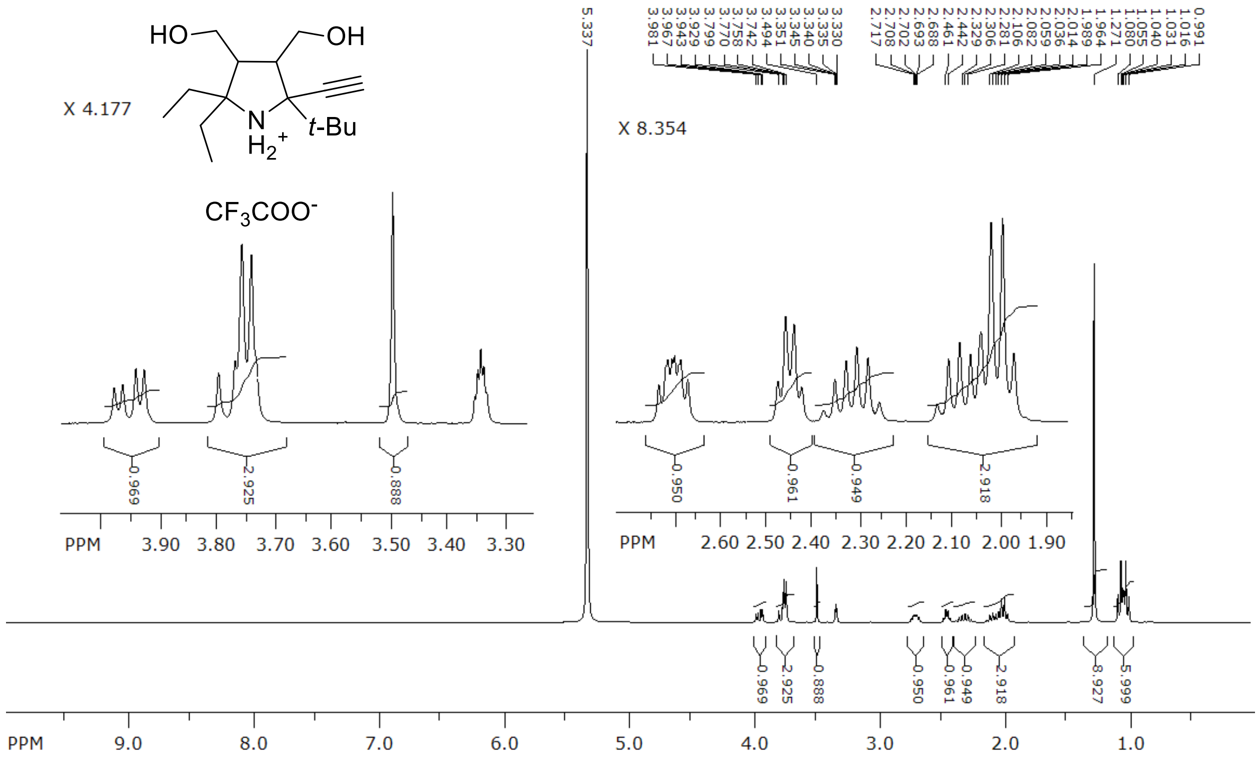

^{1}H NMR of 2,2,5,5-tetraethyl-3,4-bis(hydroxymethyl)-pyrrolidine-1-oxyl (13a)

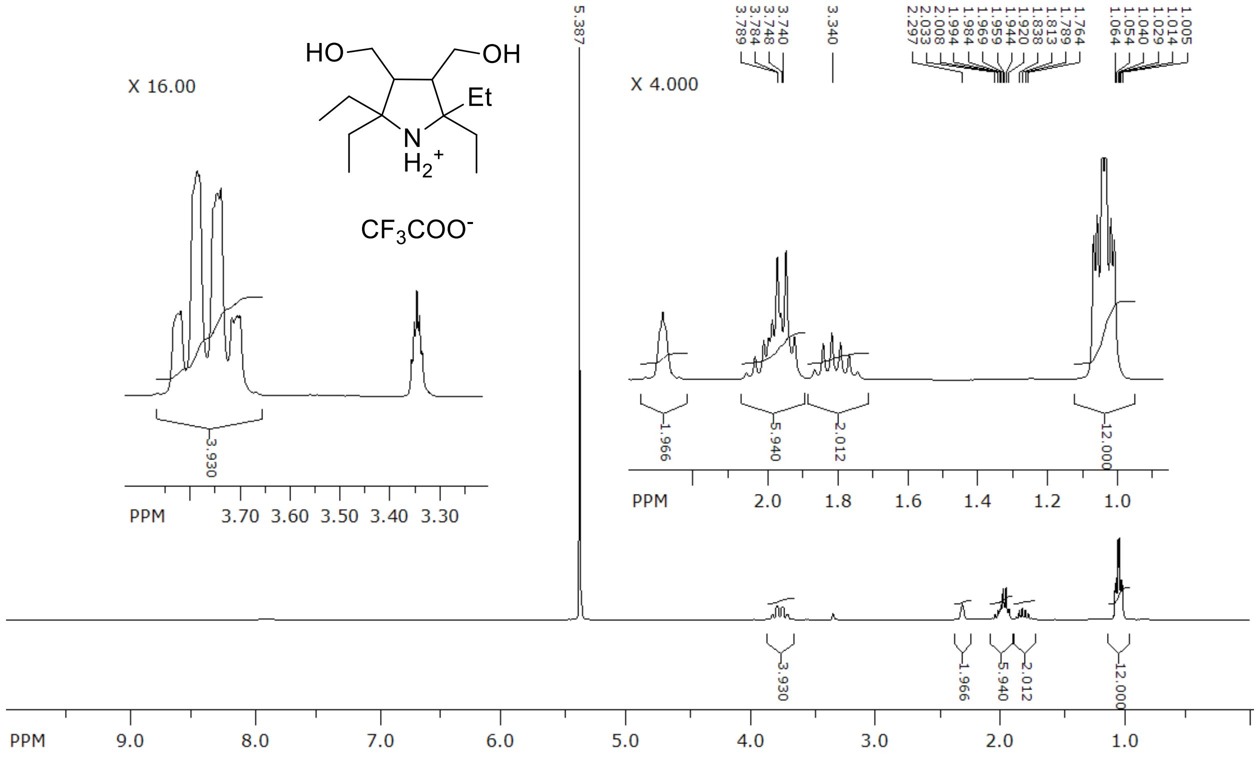

^{1}H NMR of 2,2,5-triethyl-5-isopropyl-3,4-bis(hydroxymethyl)-pyrrolidine-1-oxyl (13c)

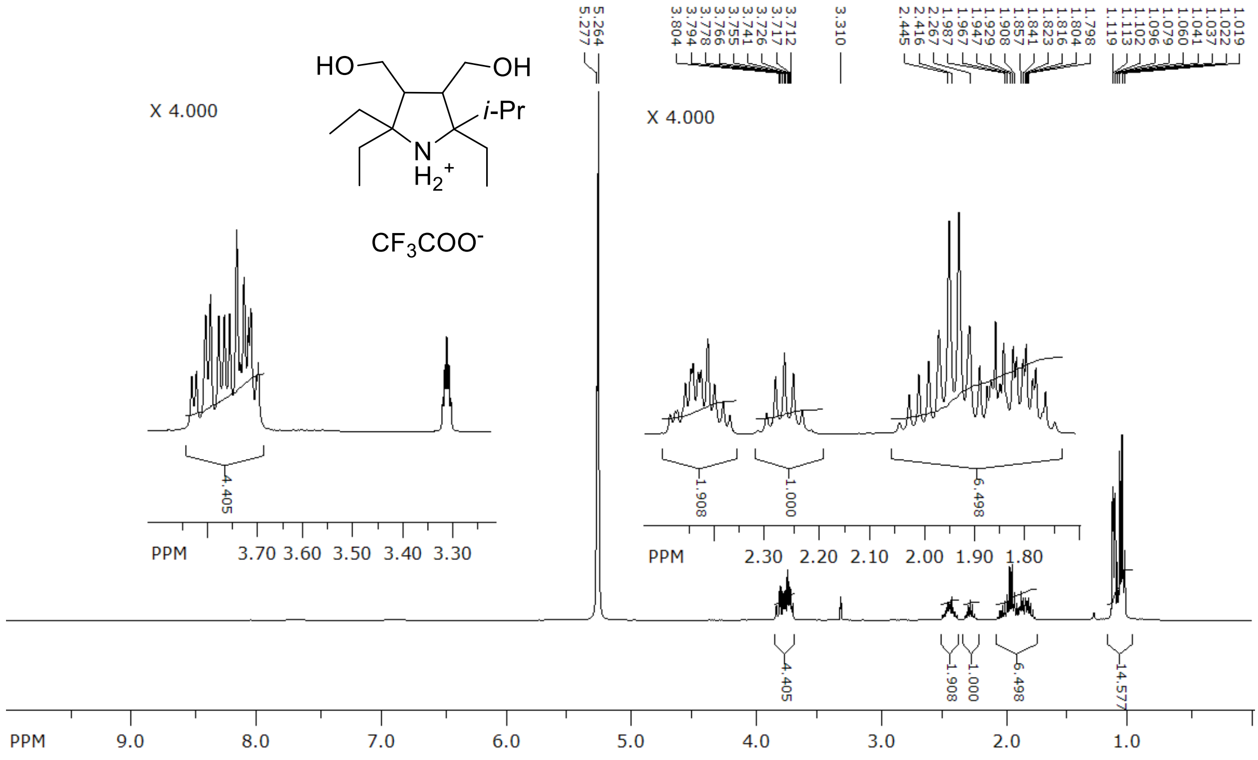

^{1}H NMR of 2,2,5-triethyl-5-tert-butyl-3,4-bis(hydroxymethyl)-pyrrolidine-1-oxyl (13b)

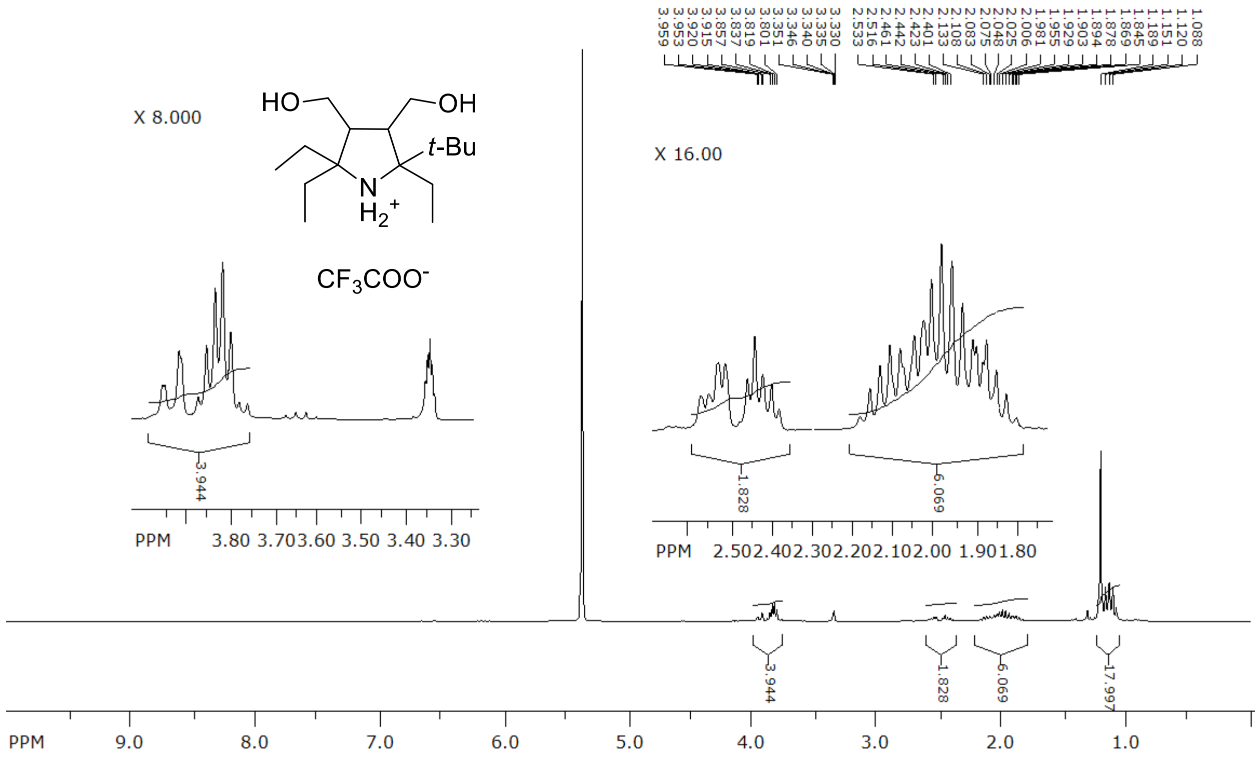

^{1}H NMR of 2,5,5-triethyl-2-ethynyl-4-pyrrolidino-2,5-dihydroimidazol-1-oxyl (17)

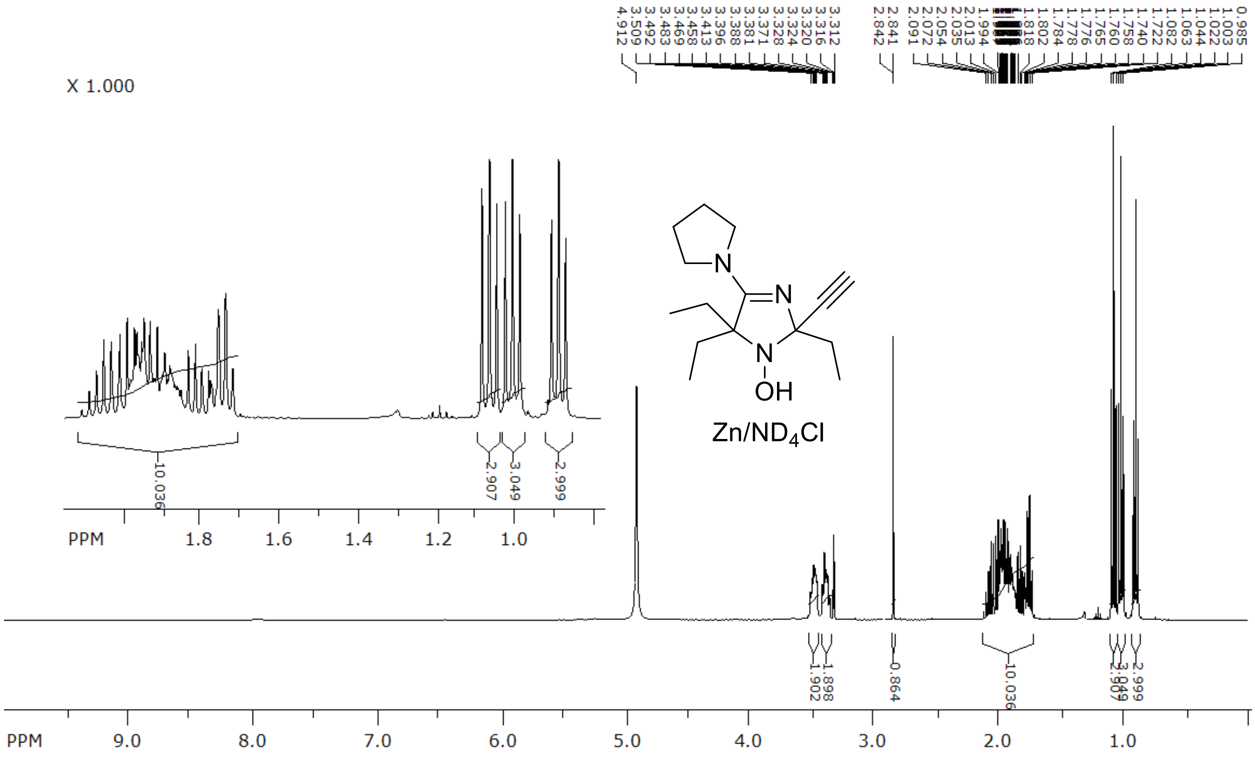

^{1}H NMR of 2,2,5,5-tetraethyl-4-pyrrolidino-2,5-dihydroimidazol-1-oxyl (21)

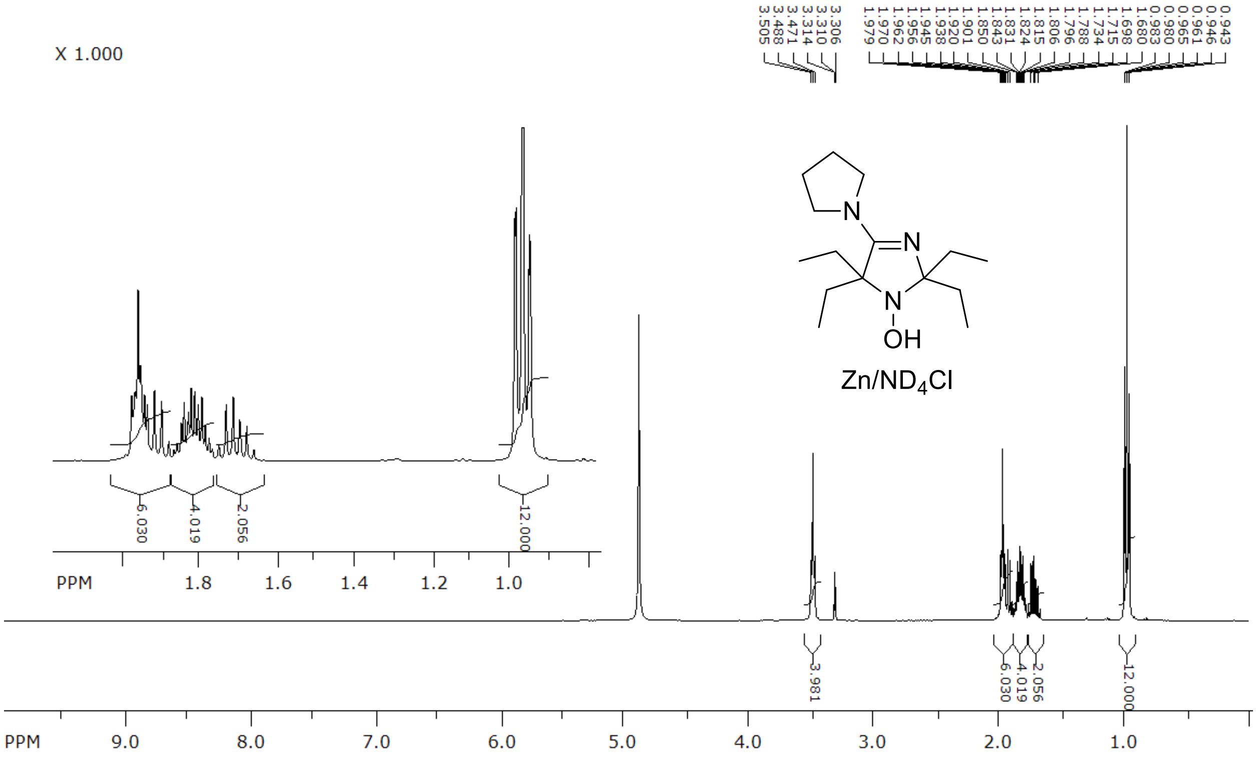

^{1}H NMR of 2,2,5-triethyl-5-ethynyl-3,4,4-trimethylimidazolidin-1-oxyl (16)

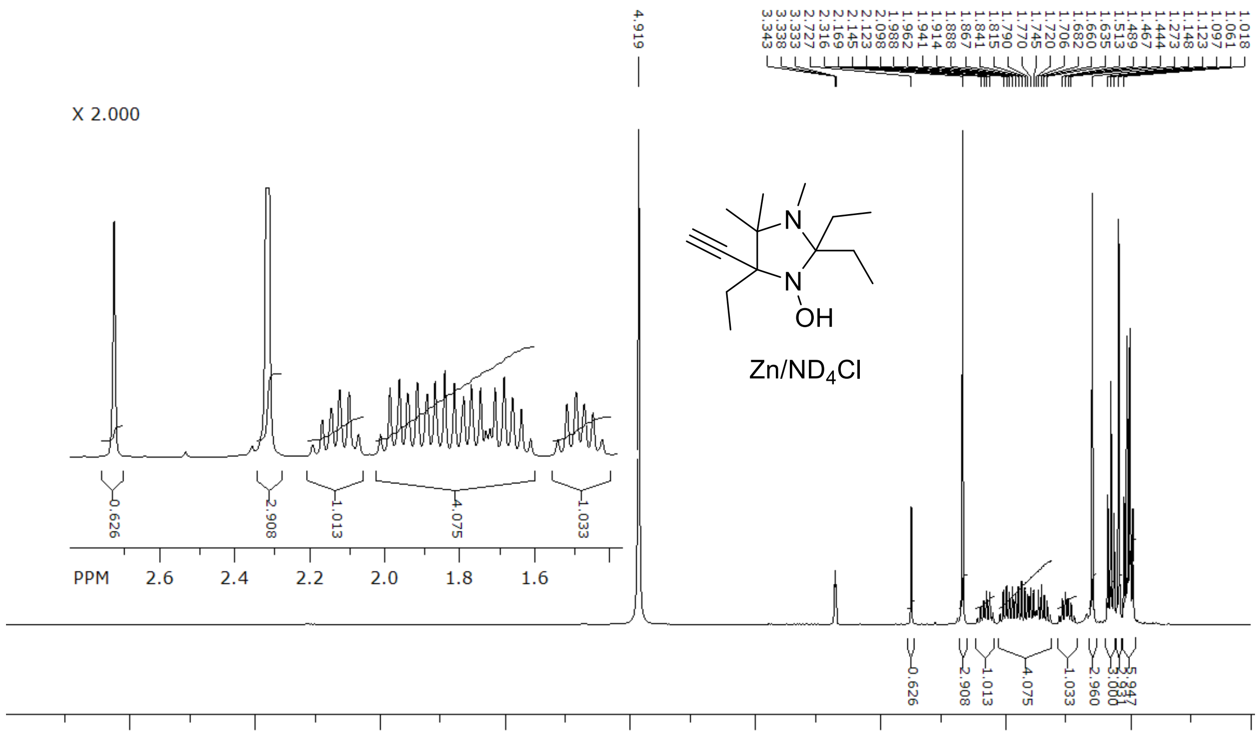

^{1}H NMR of 2,2,5,5-tetraethyl-3,4,4-trimethylimidazolidin-1-oxyl (20)

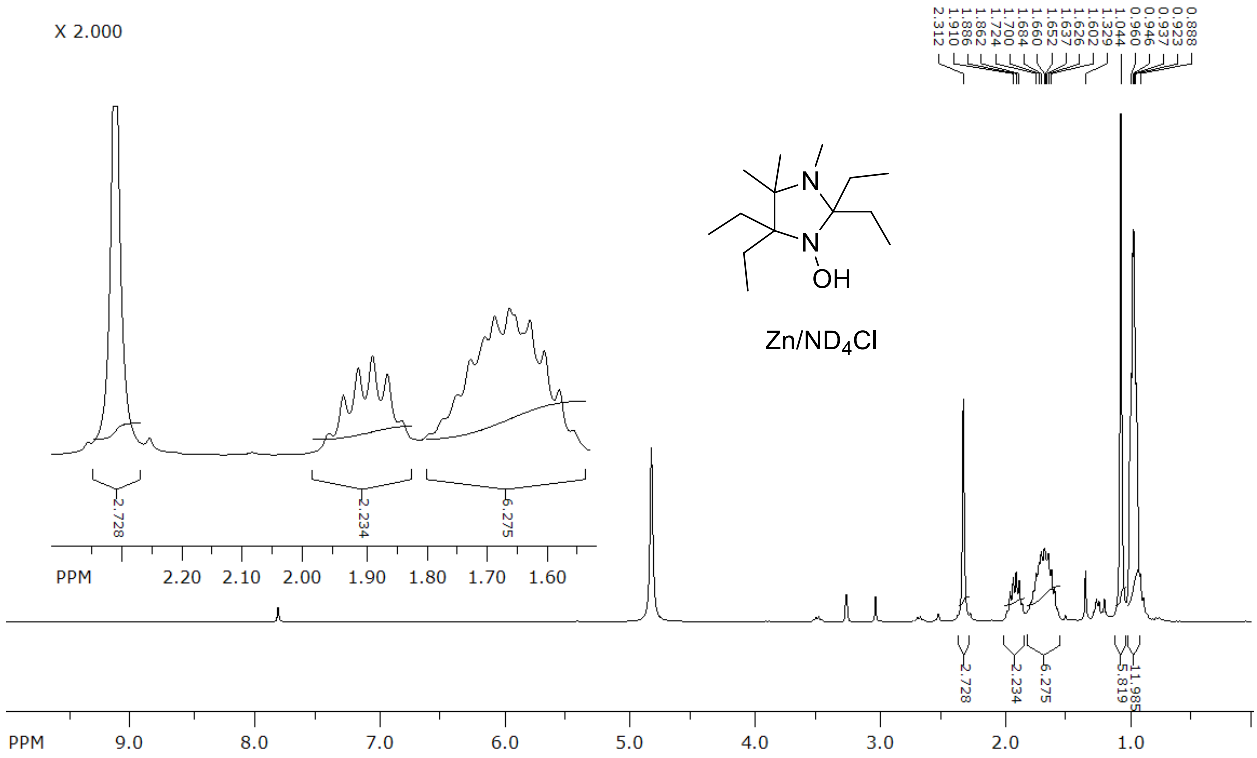

^{1}H NMR of 2,2,5-triethyl-5-ethynylpyrrolidin-1-oxyl (15)

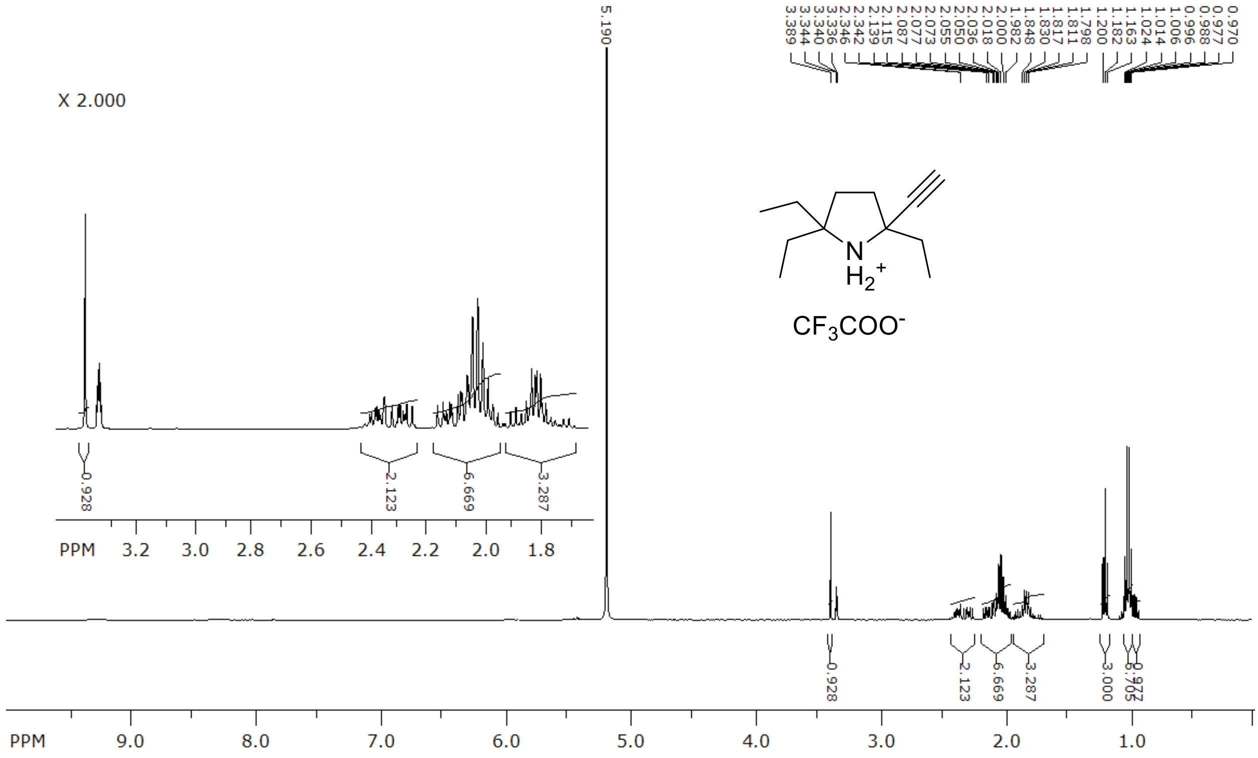

^{1}H NMR of 2,2,5,5-tetraethylpyrrolidin-1-oxyl (19)

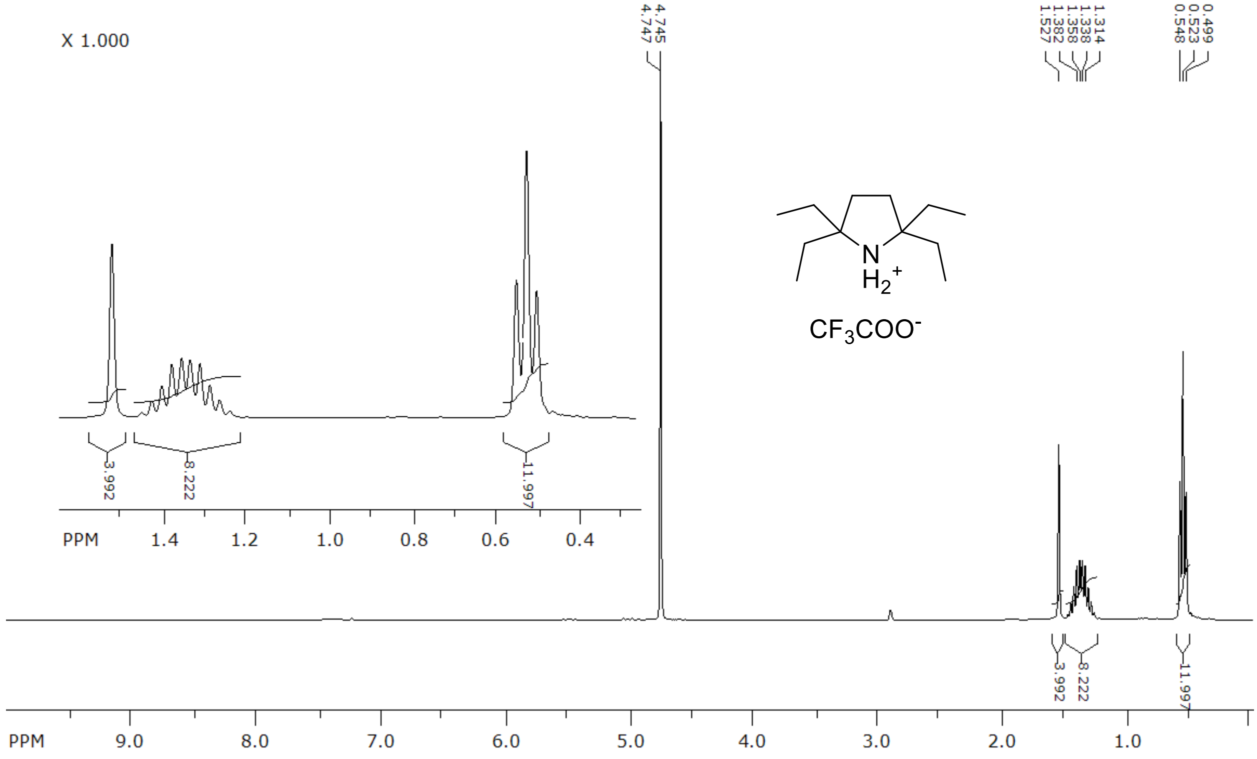

1H NMR of (3S,4S,5S)-3,4-di-tert-butoxy-2,2,5-triethyl-5-ethynylpyrrolidine 1-oxyl (14)

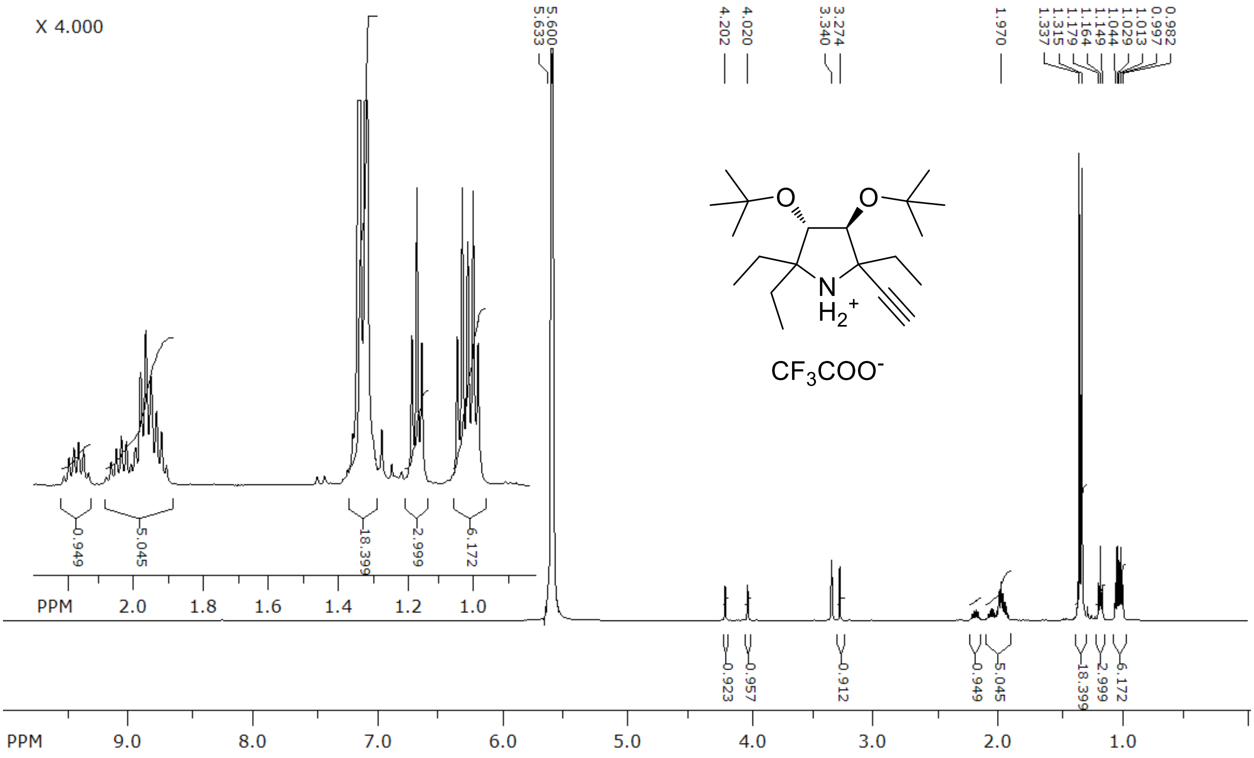

1H NMR of (3S,4S)-3,4-di-tert-butoxy-2,2,5,5-tetraethylpyrrolidine 1-oxyl (18)

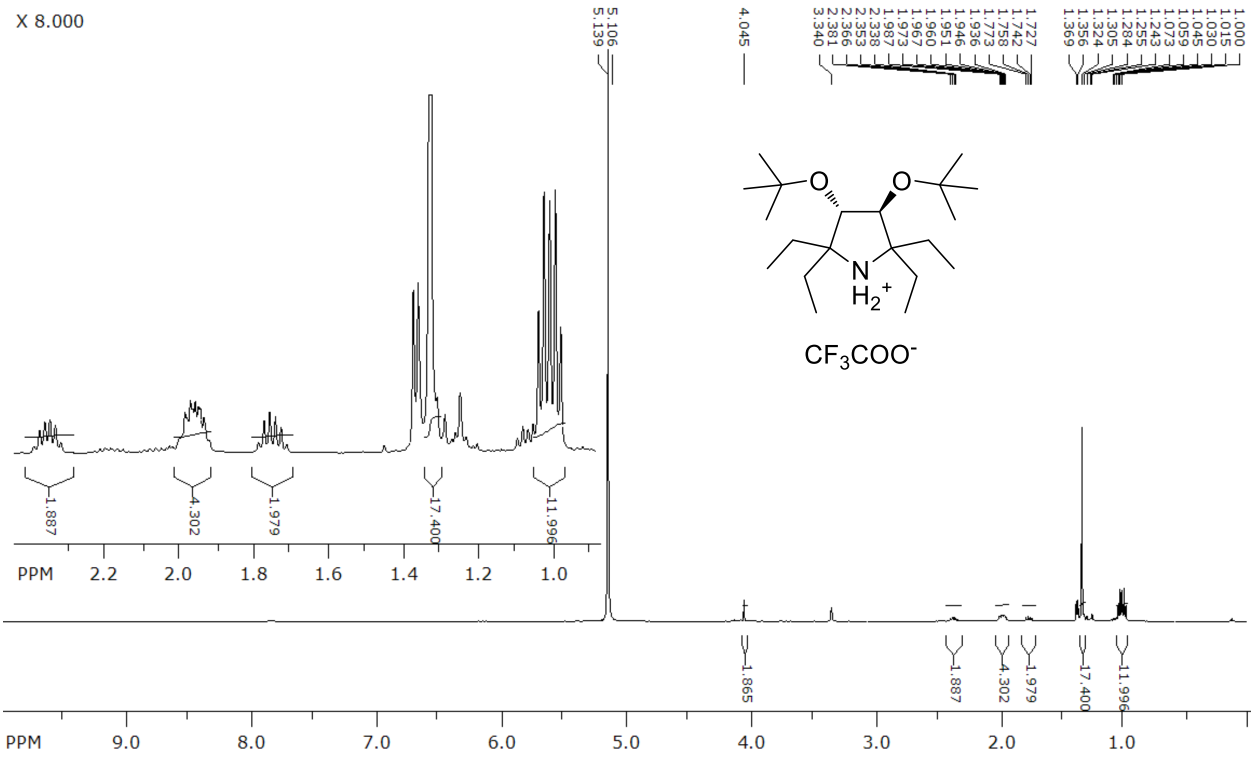

¹H NMR of 2,2,5-triethyl-5-(3-hydroxyprop-1-yn-1-yl)pyrrolidin-1-oxyl (23)

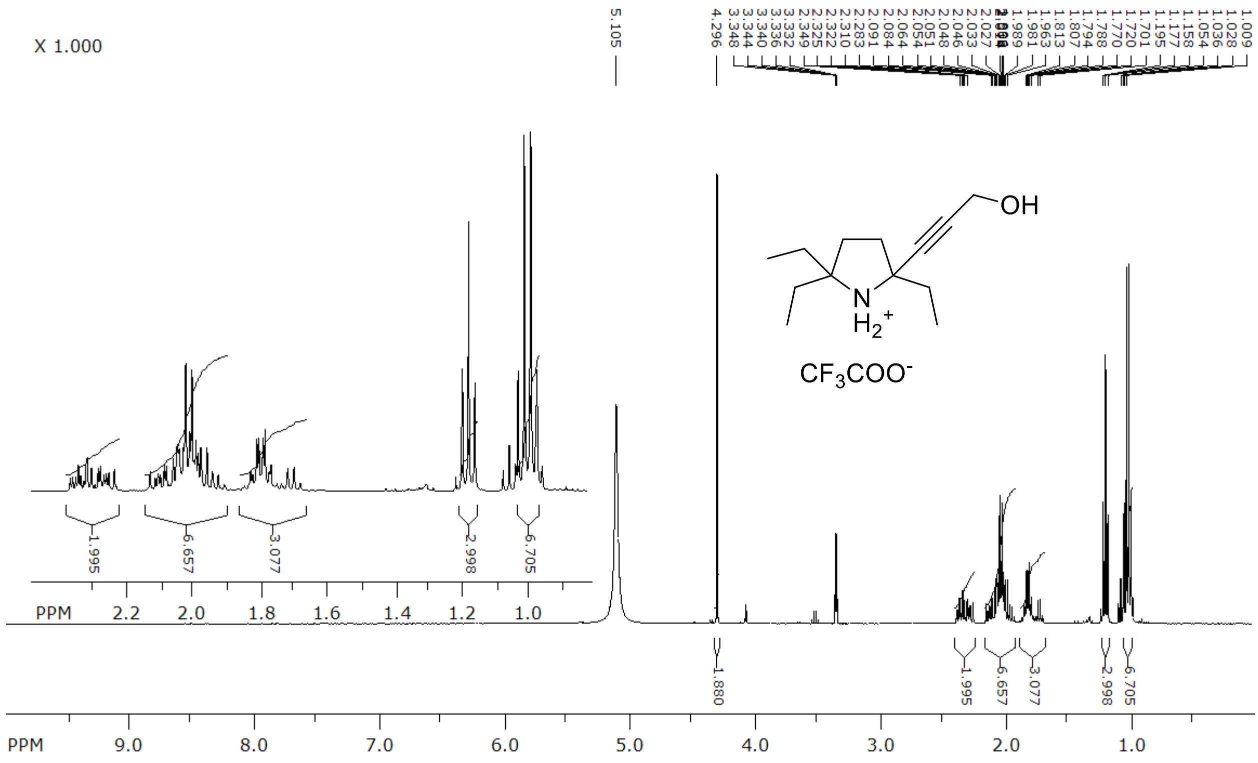

¹H NMR of 2,2,5-triethyl-5-phenylethynyl-3,4-bis(hydroxymethyl)-pyrrolidine-1-oxyl (22a)

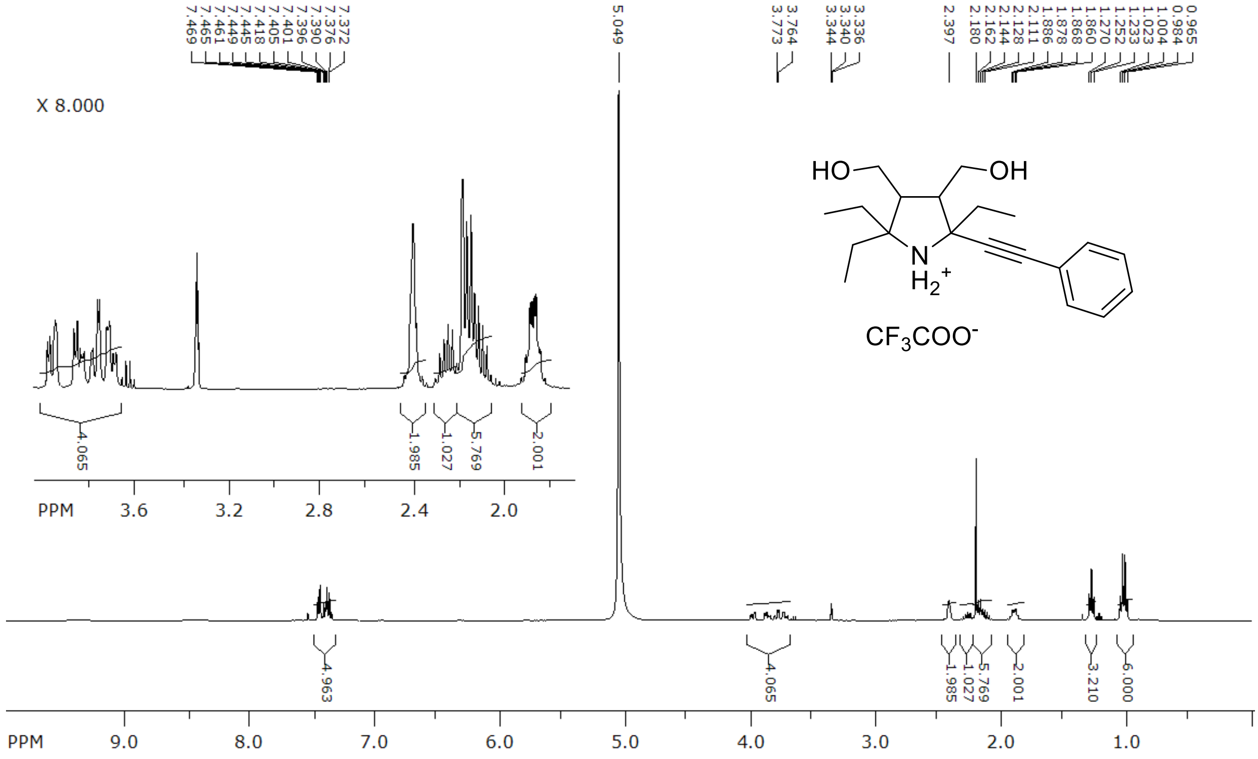

^{1}H NMR of 2,2,5-triethyl-5-(3-hydroxyprop-1-yn-1-yl)-3,4-bis(hydroxymethyl)-pyrrolidine-1-oxyl (22b)

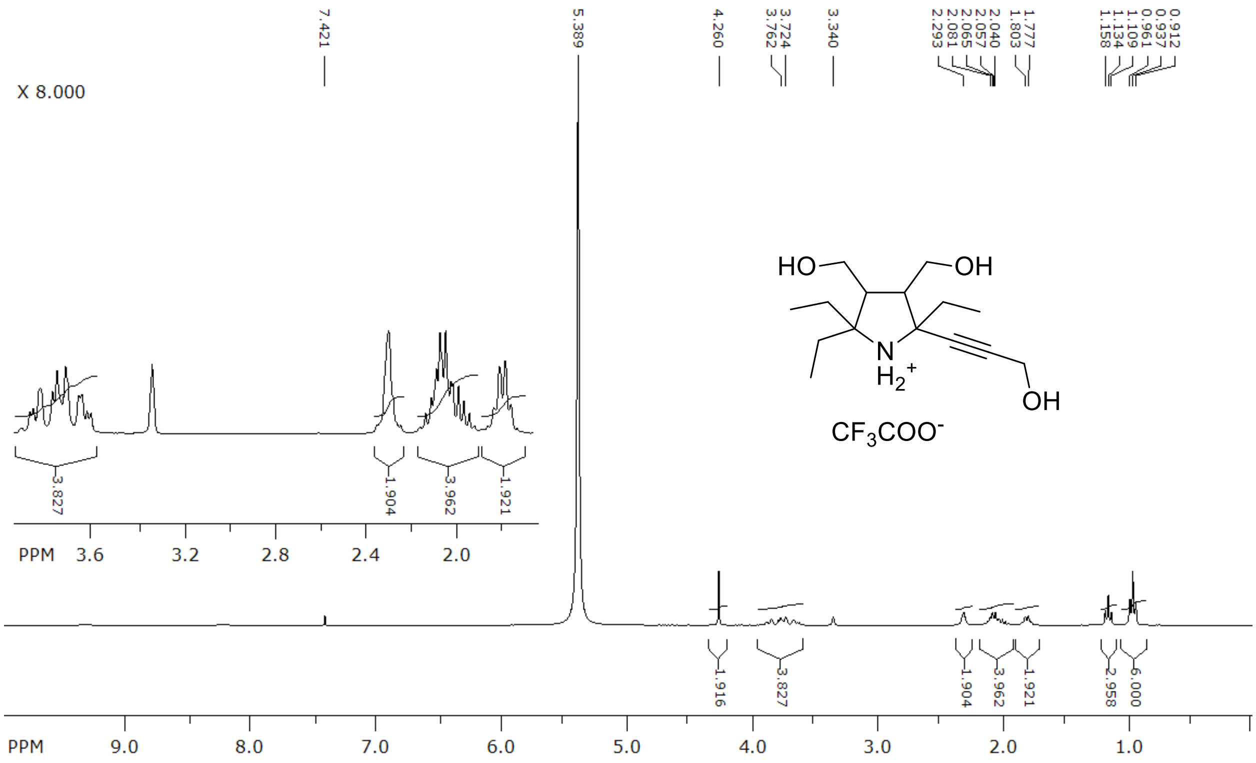

^{1}H NMR of 2,2,5-triethyl-5-(3-hydroxy-3-methylbut-1-yn-1-yl)-3,4-bis(hydroxymethyl)-pyrrolidine-1-oxyl (22c)

CD3OD

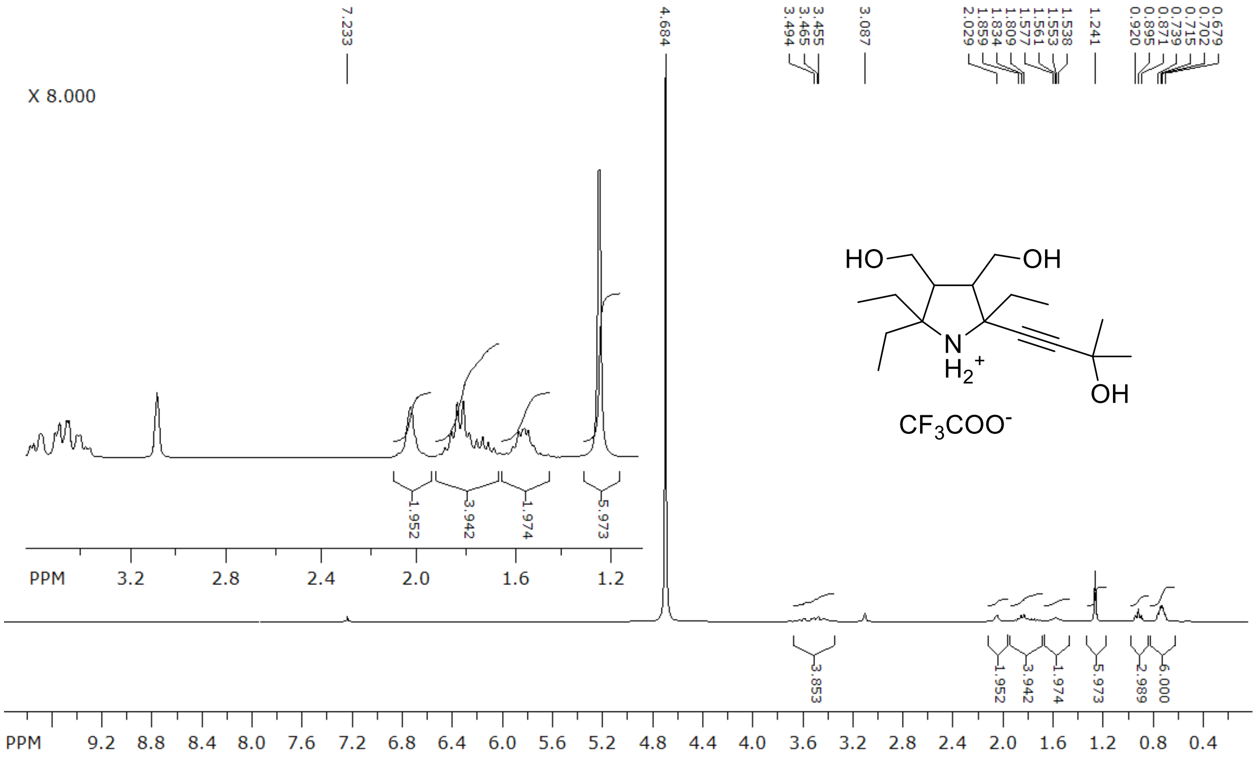

IR (neat) of 2,2-diethyl-5-isopropyl-3,4-bis(methoxycarbonyl)pyrrolidine (6c)

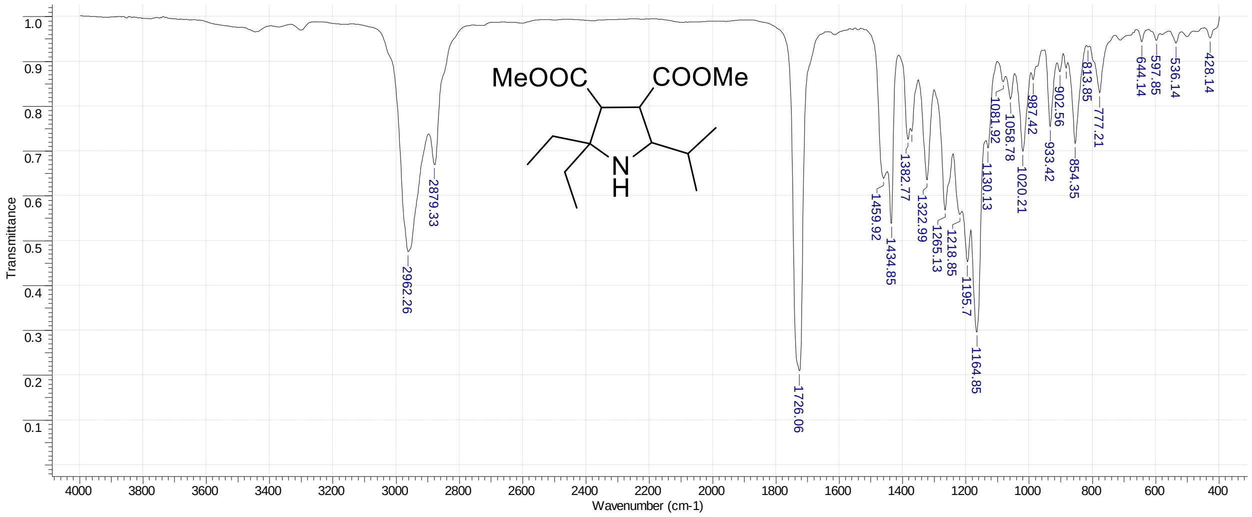

IR (neat) of 2,2-diethyl-5-isopropyl-3,4-bis(methoxycarbonyl)pyrrolidine (7c)

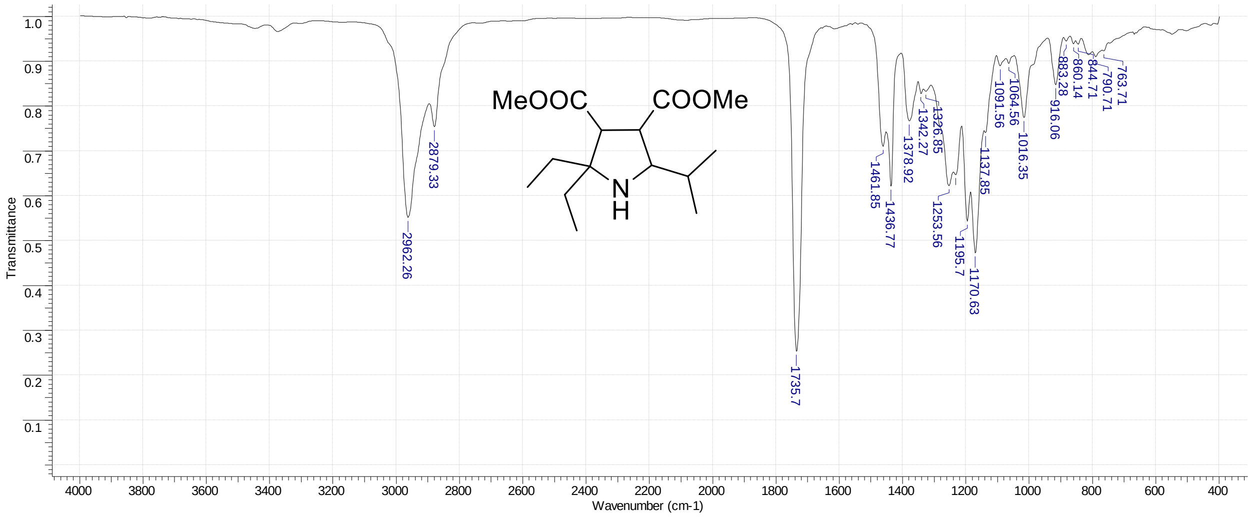

IR (neat) of 2,2,5-triisopropyl-3,4-bis(methoxycarbonyl)pyrrolidine (6d)

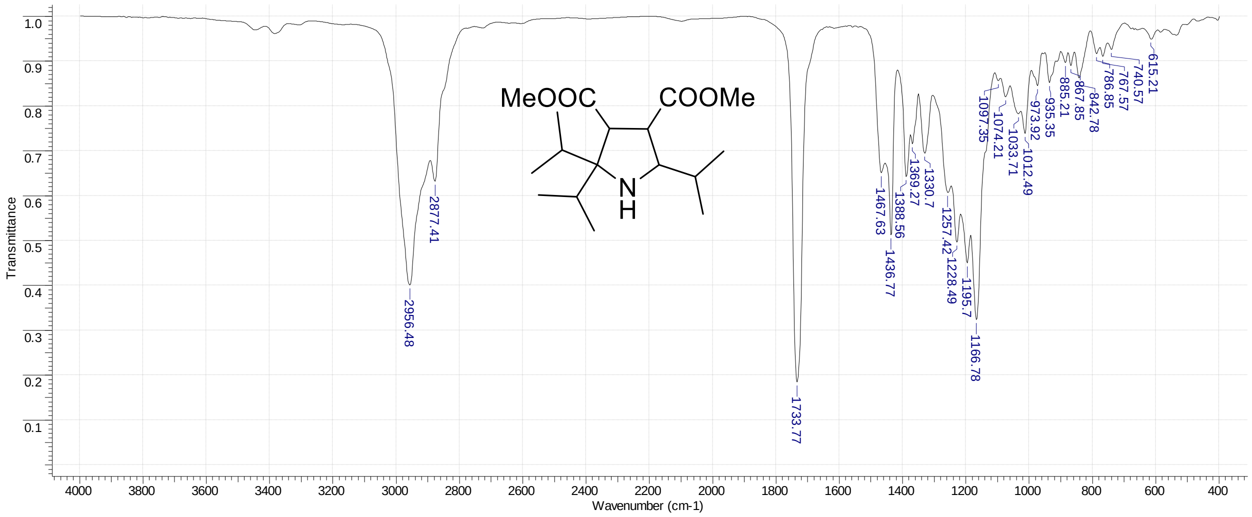

IR (neat) of 2,2,5-triisopropyl-3,4-bis(methoxycarbonyl)pyrrolidine (7d)

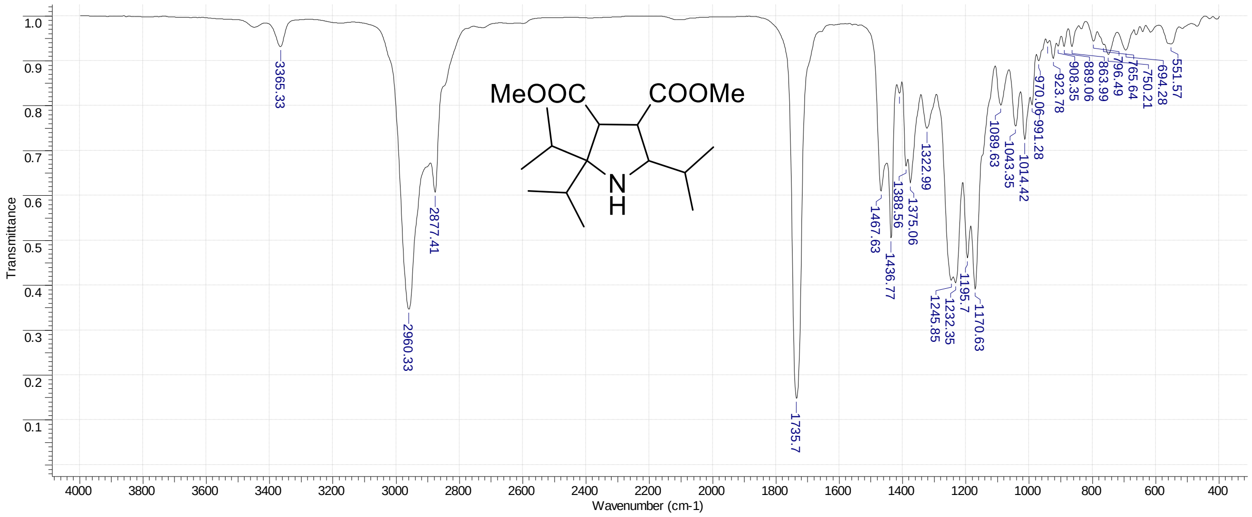

IR (KBr) of 2,2-diethyl-5-isopropyl-3,4-bis(hydroxymethyl)-3,4-dihydro-2H-pyrrole 1-oxide (8c)

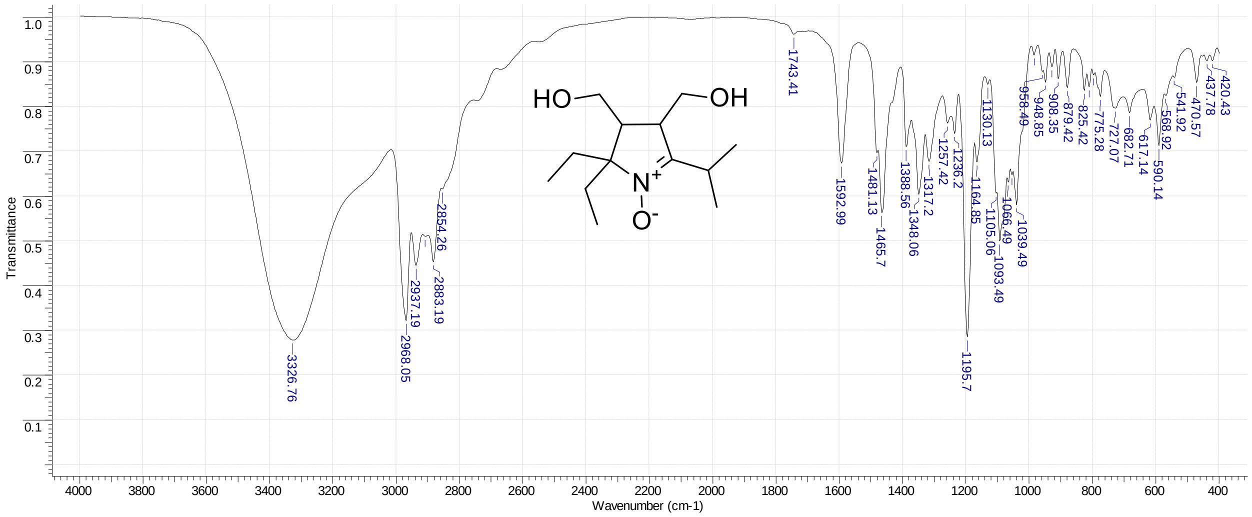

IR (KBr) of 2,2,5-triisopropyl-3,4-bis(hydroxymethyl)-3,4-dihydro-2H-pyrrole 1-oxide (8d)

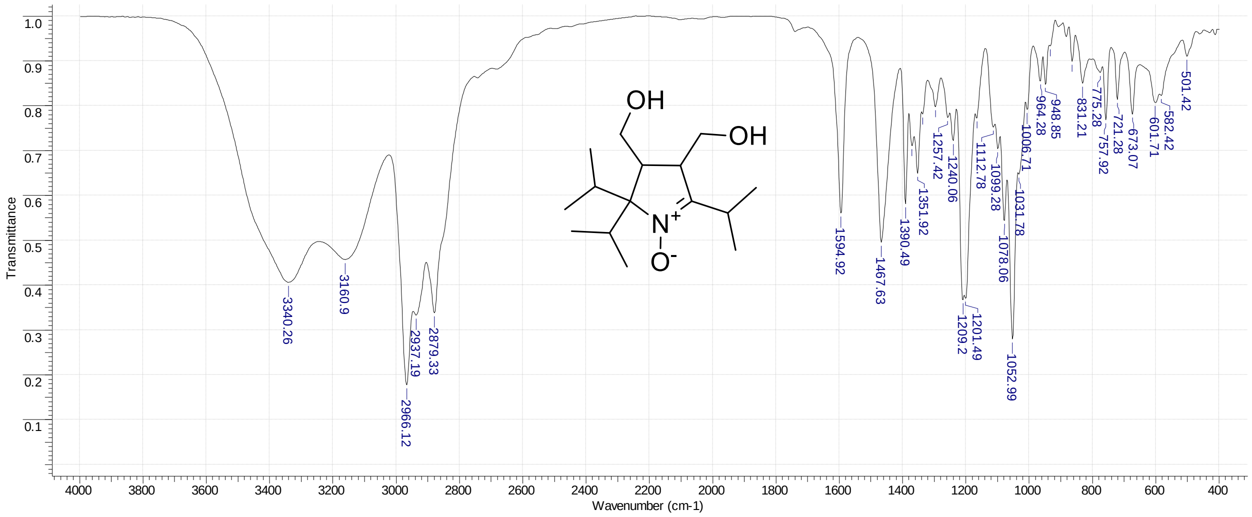

IR (neat) of 2,2,5-triethyl-3,4-bis(((2-methoxypropan-2-yl)oxy)methyl)-3,4-dihydro-2H-pyrrole 1-oxide (1a)

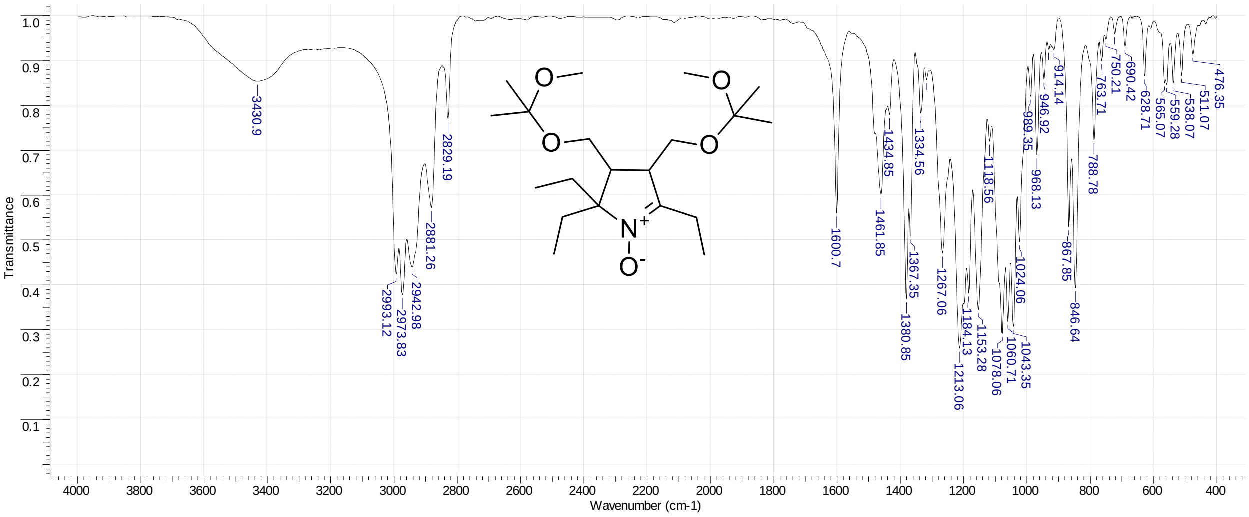

IR (neat) of 2,2-diethyl-5-isopropyl-3,4-bis(((2-methoxypropan-2-yl)oxy)methyl)-3,4-dihydro-2H-pyrrole 1-oxide (1c)

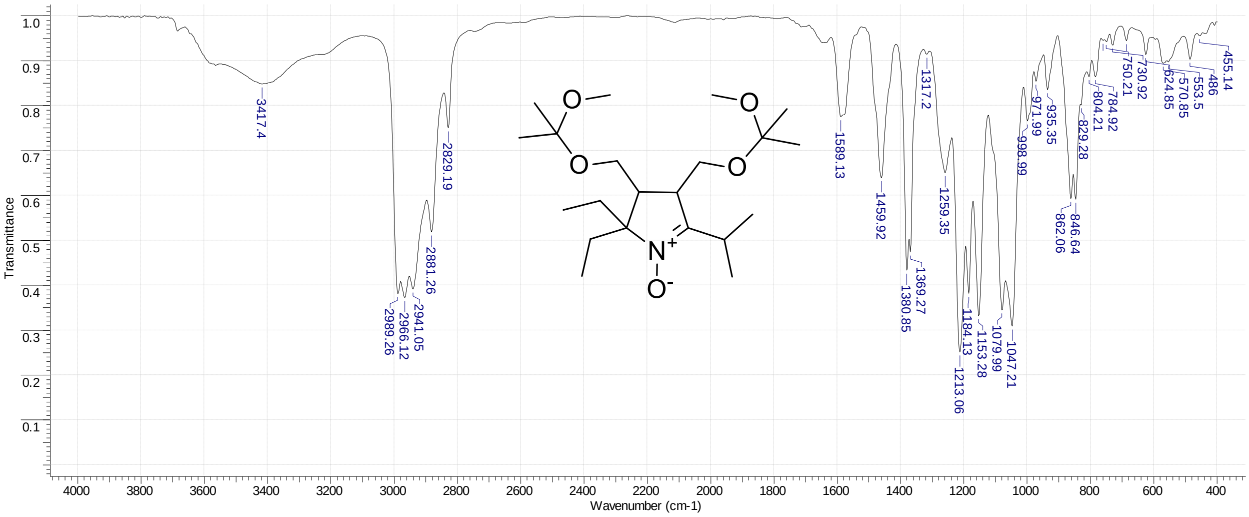

IR (KBr) of 5-tert-butyl-2,2-diethyl-3,4-bis(((2-methoxypropan-2-yl)oxy)methyl)-3,4-dihydro-2H-pyrrole 1-oxide (1b)

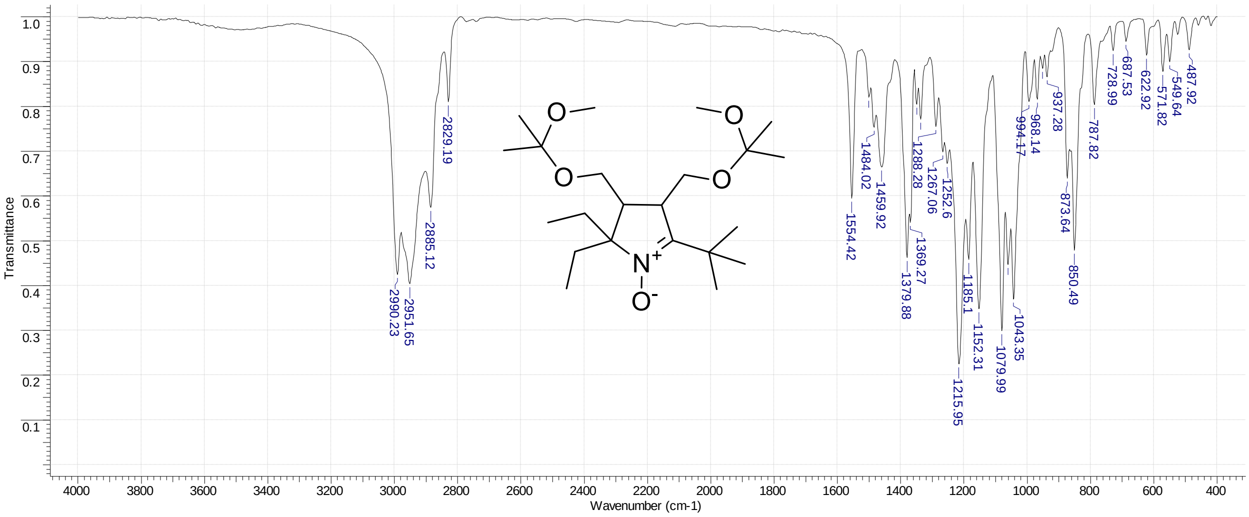

IR (KBr) of 2,2,5-triisopropyl-3,4-bis(((2-methoxypropan-2-yl)oxy)methyl)-3,4-dihydro-2H-pyrrole 1-oxide (1d)

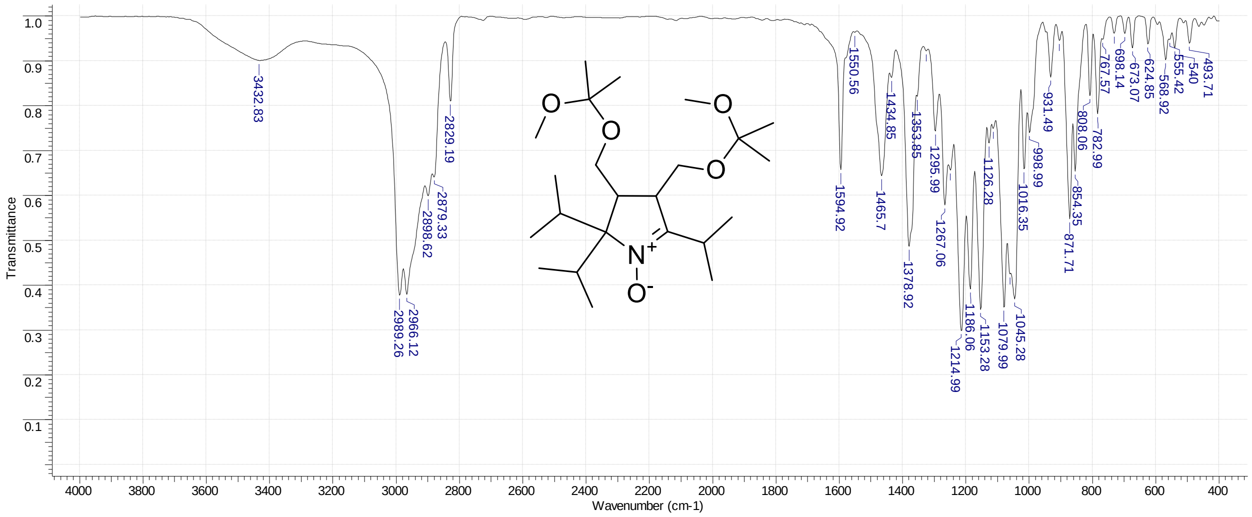

IR (KBr) of (3S,4S)-3,4-di-tert-butoxy-2,2,5-triethyl-3,4-dihydro-2H-pyrrole 1-oxide (2)

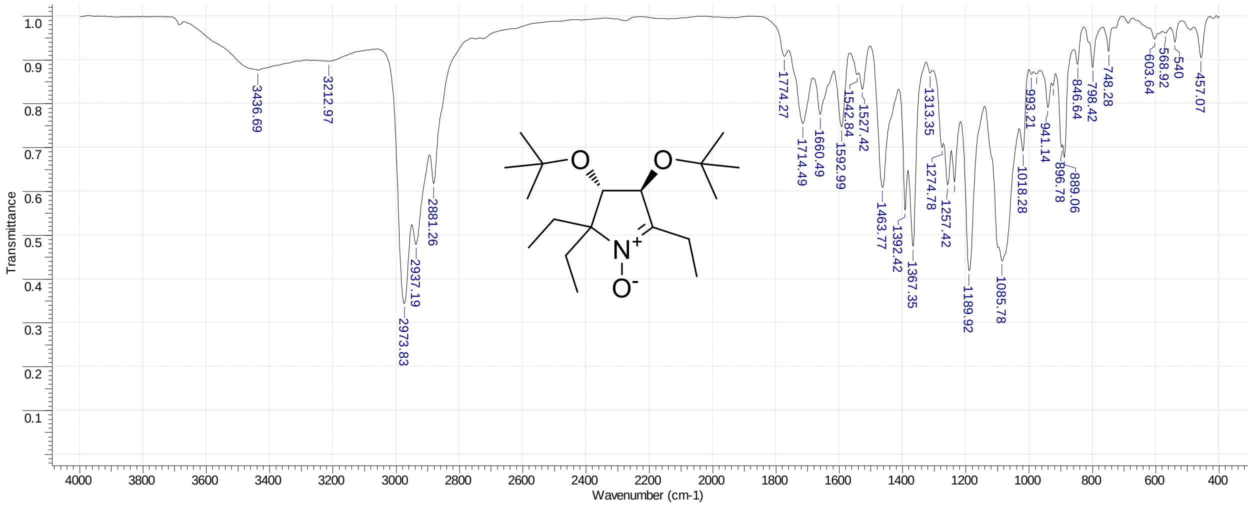

IR (KBr) of 2,2,4-triethyl-5,5-dimethyl-2,5-dihydroimidazole 3-oxide (11)

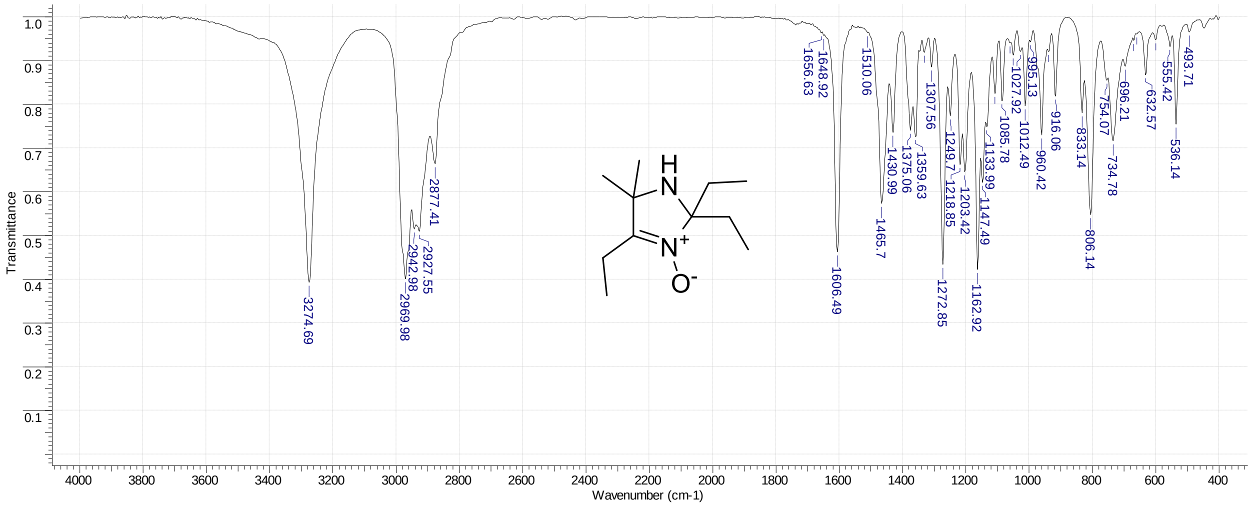

IR (neat) of 2,2,4-triethyl-1,5,5-trimethyl-2,5-dihydroimidazole 3-oxide (4)

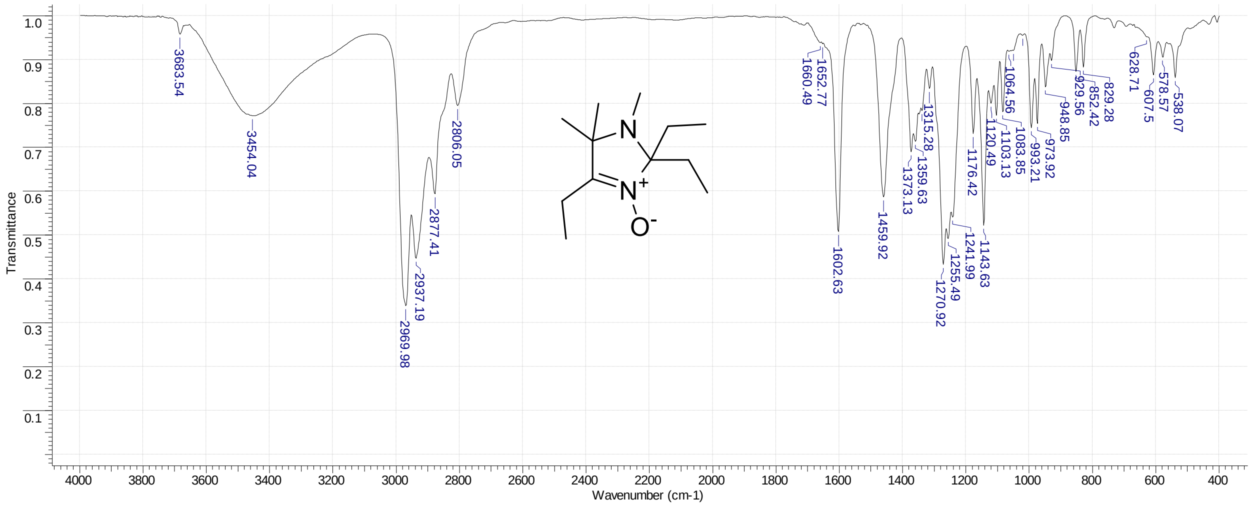

IR (KBr) of 2,2,5-triethyl-5-ethynyl-3,4-bis(hydroxymethyl)-pyrrolidine-1-oxyl (12a)

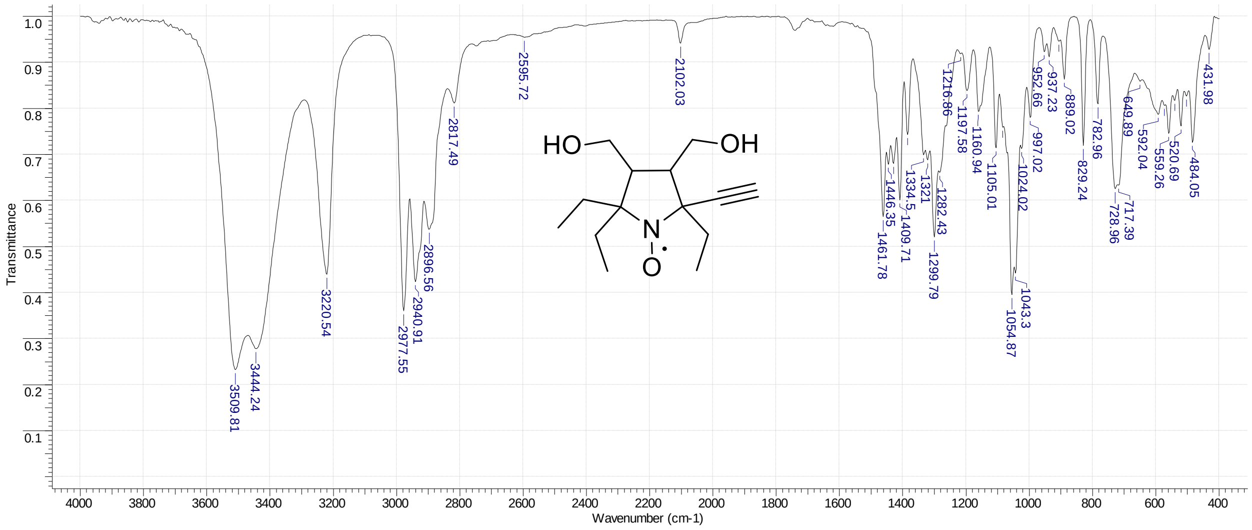

IR (KBr) of 2,2-diethyl-5-isopropyl-5-ethynyl-3,4-bis(hydroxymethyl)-pyrrolidine-1-oxyl (12c)

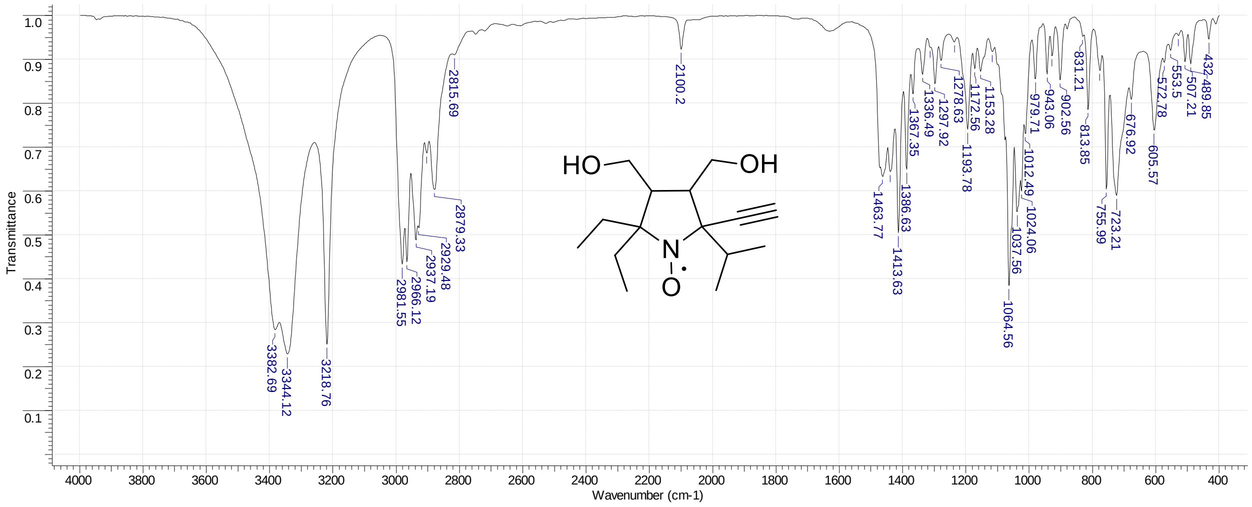

IR (KBr) of 5-tert-dutyl-2,2-diethyl-5-ethynyl-3,4-bis(hydroxymethyl)-pyrrolidine-1-oxyl (12b)

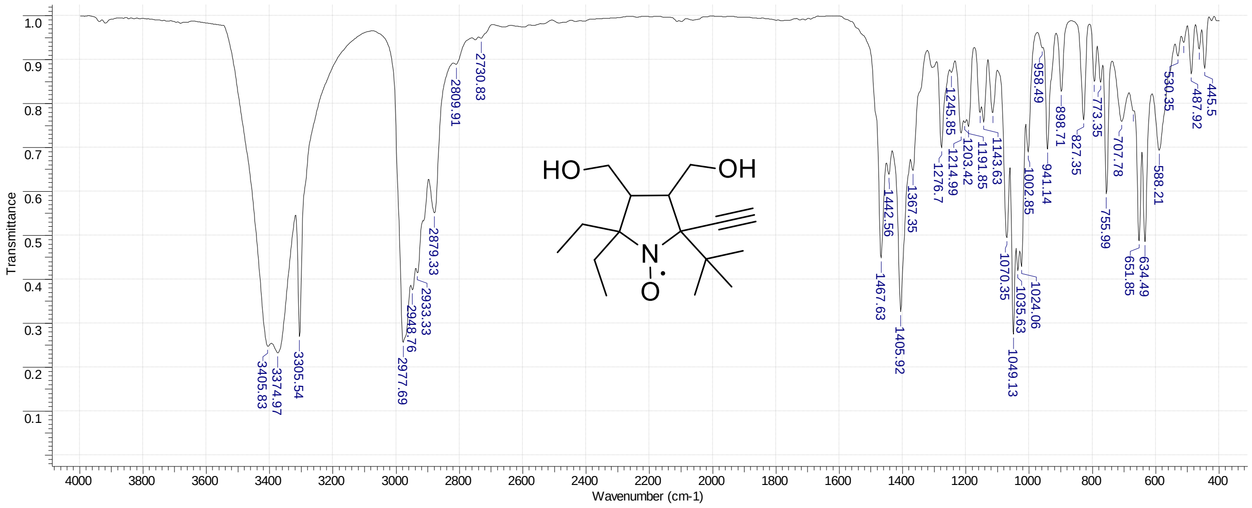

IR (KBr) of 2,2,5,5-tetraethyl-3,4-bis(hydroxymethyl)-pyrrolidine-1-oxyl (13a)

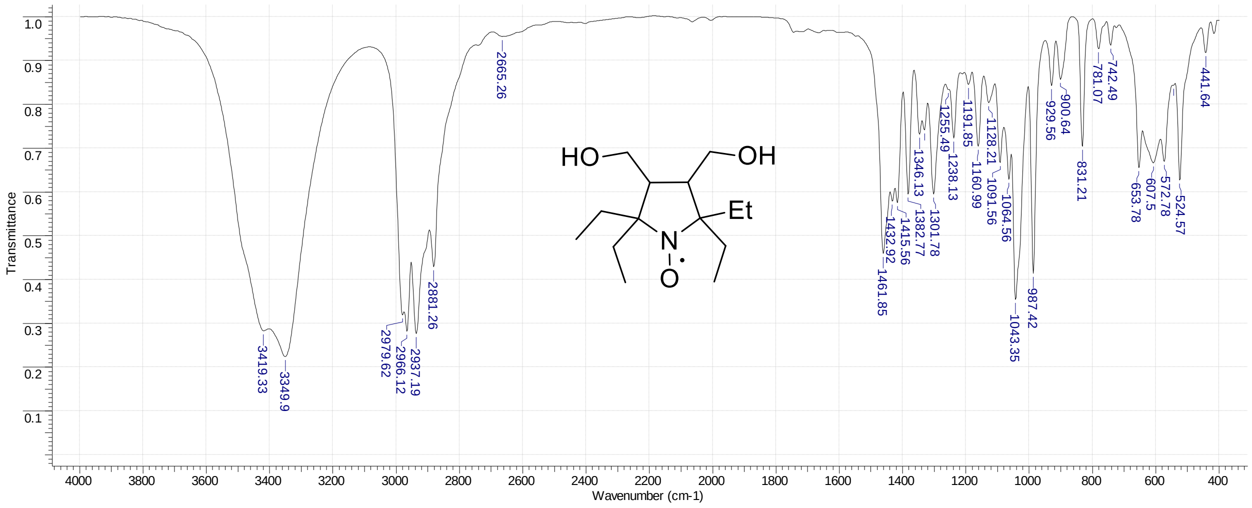

IR (neat) of 2,2,5-triethyl-5-isopropyl-3,4-bis(hydroxymethyl)-pyrrolidine-1-oxyl (13c)

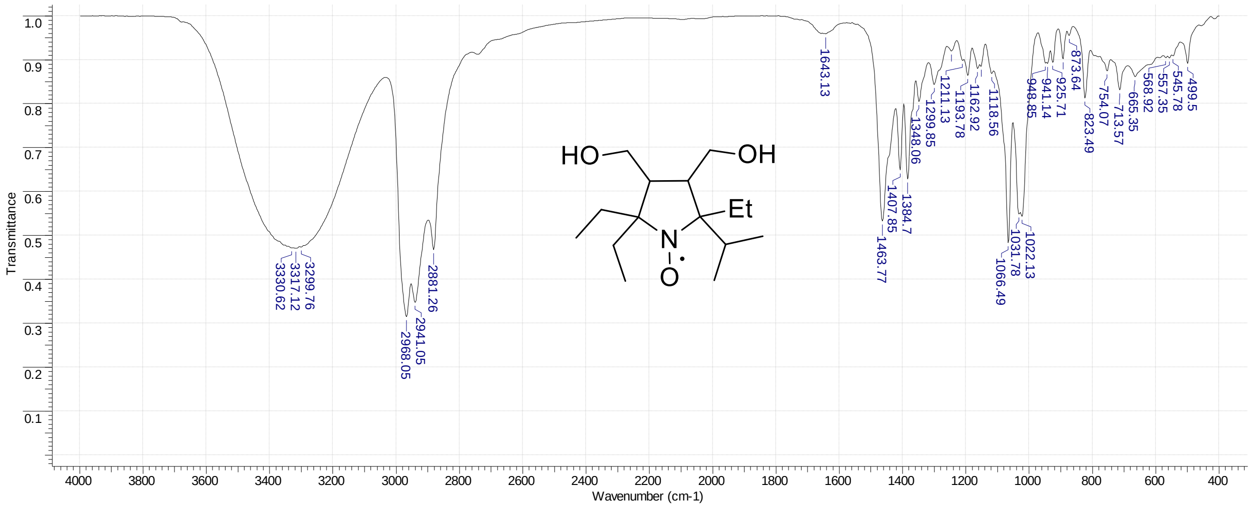

IR (KBr) of 2,2,5-triethyl-5-tert-butyl-3,4-bis(hydroxymethyl)-pyrrolidine-1-oxyl (13b)

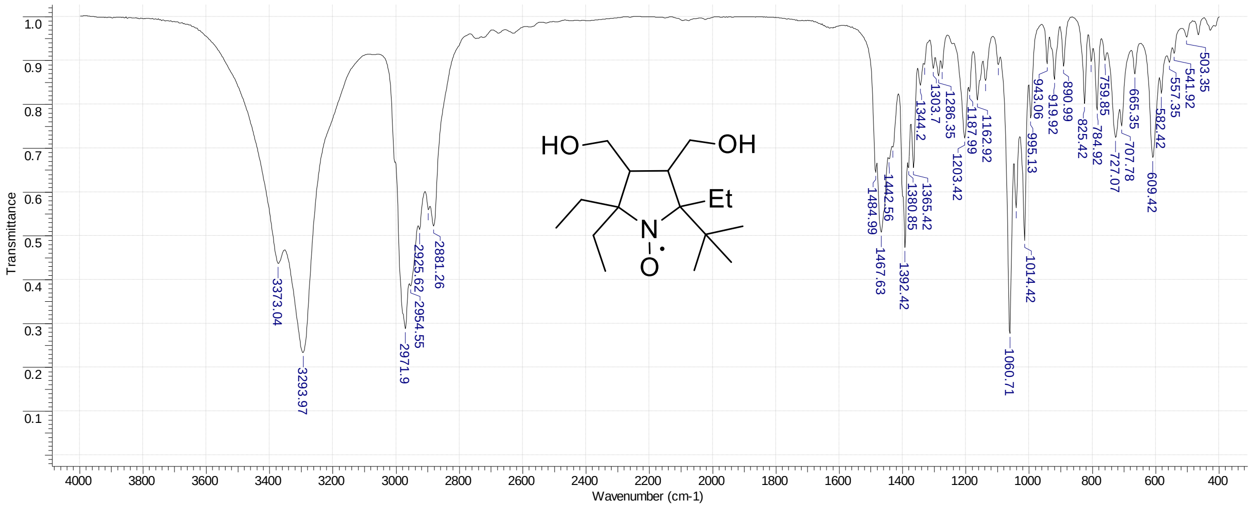

IR (KBr) of 2,5,5-triethyl-2-ethynyl-4-pyrrolidino-2,5-dihydroimidazol-1-oxyl (17)

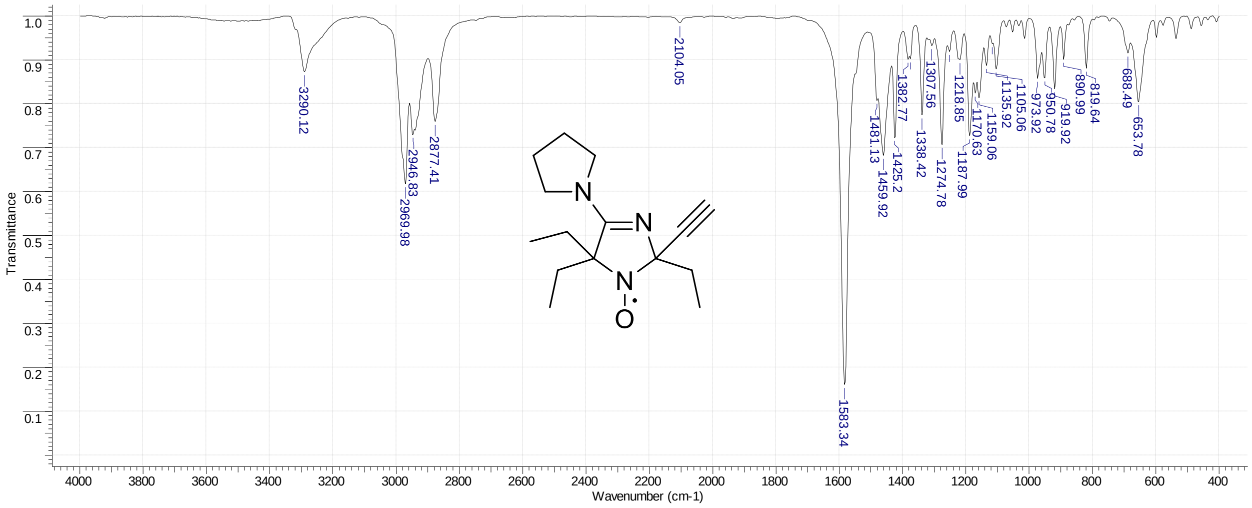

IR (neat) of 2,2,5,5-tetraethyl-4-pyrrolidino-2,5-dihydroimidazol-1-oxyl (21)

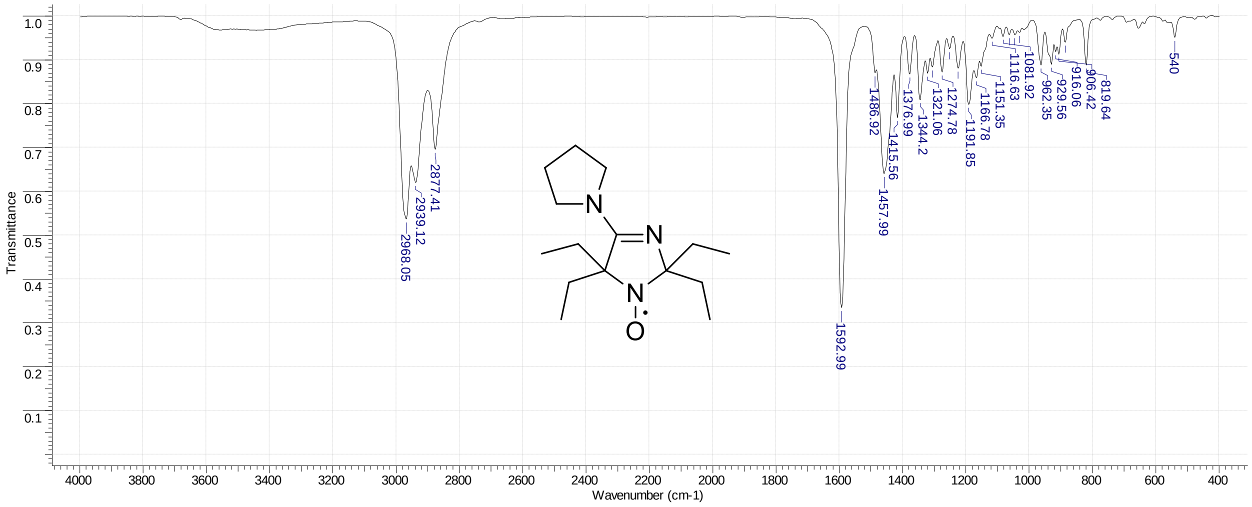

IR (KBr) of 2,2,5-triethyl-5-ethynyl-3,4,4-trimethylimidazolidin-1-oxyl (16)

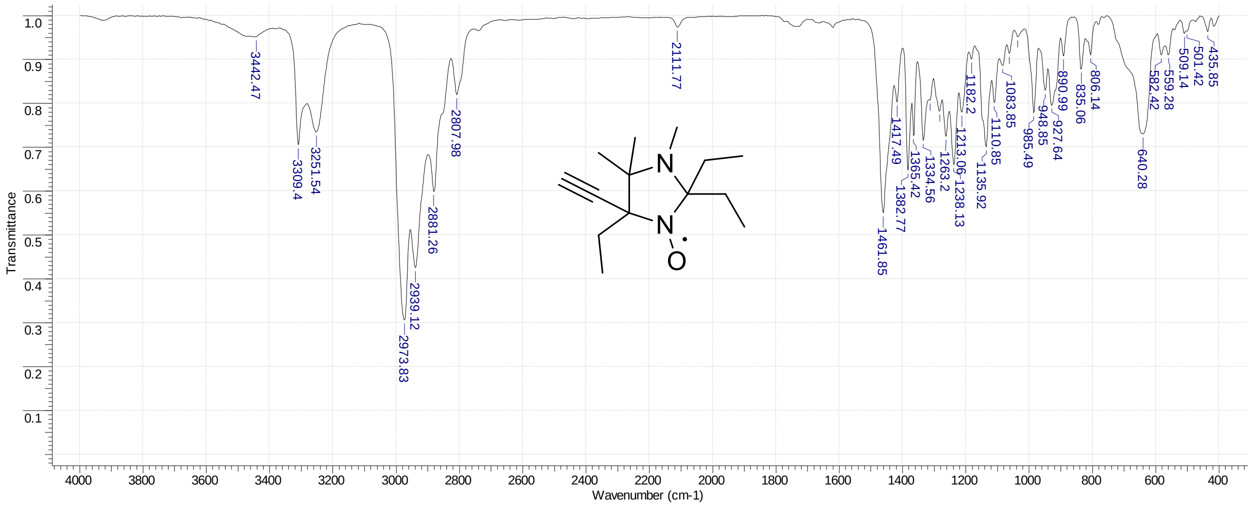

IR (KBr) of 2,2,5,5-tetraethyl-3,4,4-trimethylimidazolidin-1-oxyl (20)

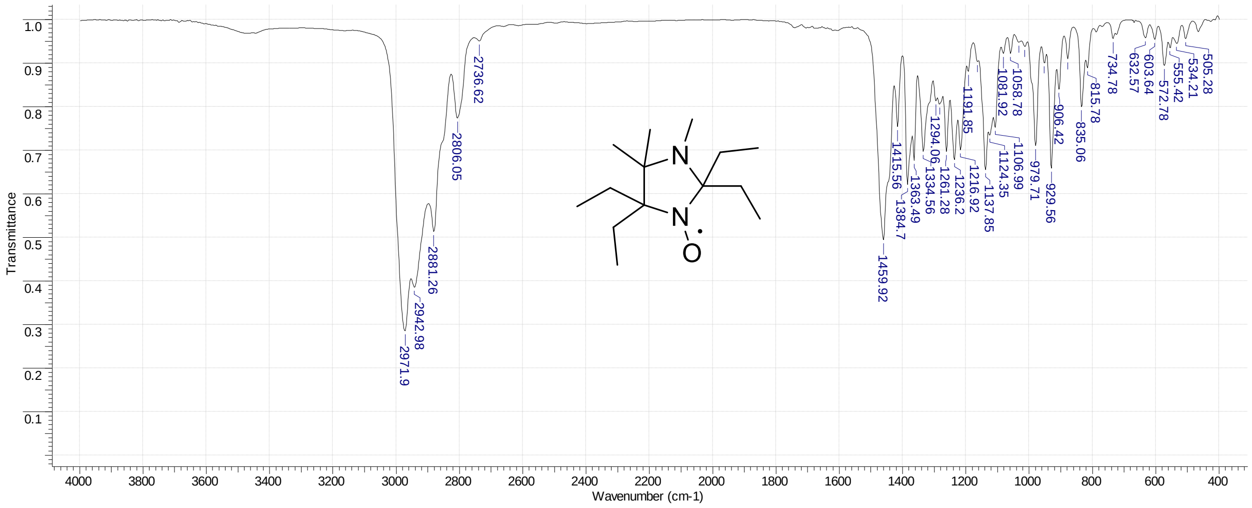

IR (neat) of 2,2,5-triethyl-5-ethynylpyrrolidin-1-oxyl (15)

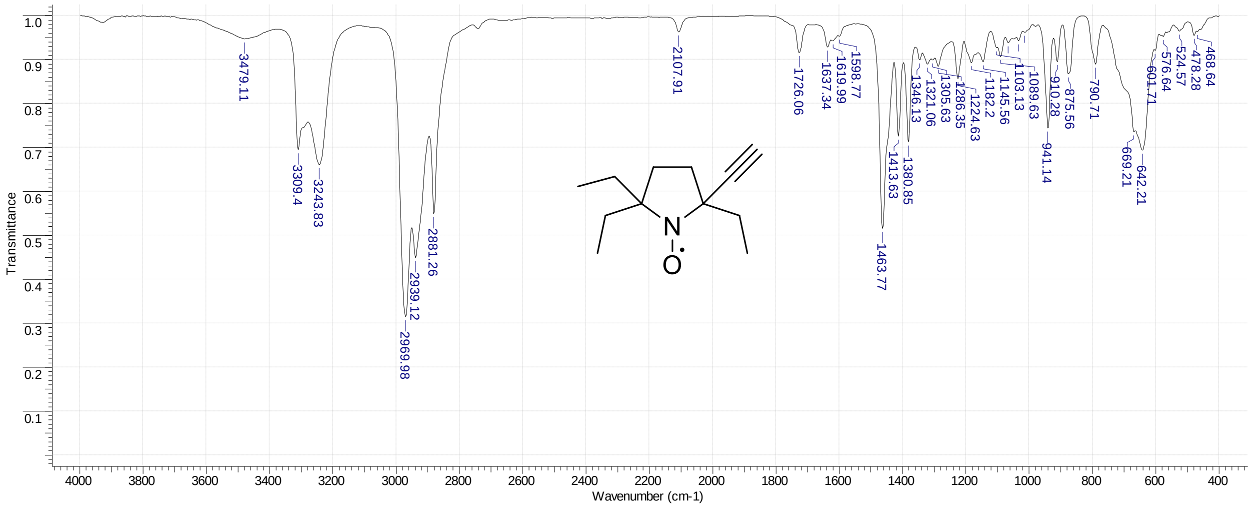

IR (neat) of 2,2,5,5-tetraethylpyrrolidin-1-oxyl (19)

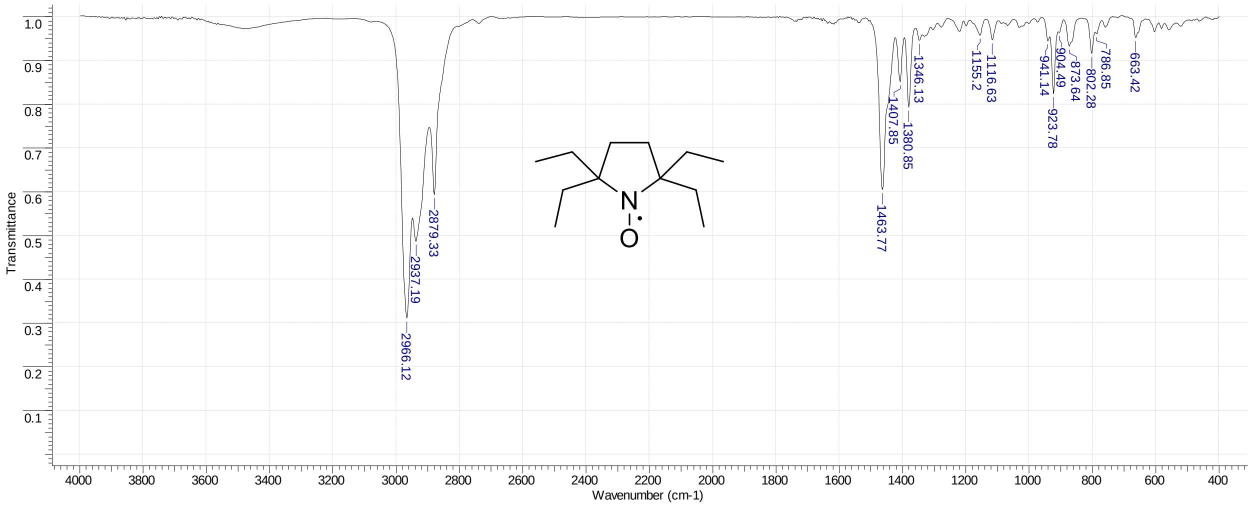

IR (KBr) of (3S,4S,5S)-3,4-di-tert-butoxy-2,2,5-triethyl-5-ethynylpyrrolidine 1-oxyl (14)

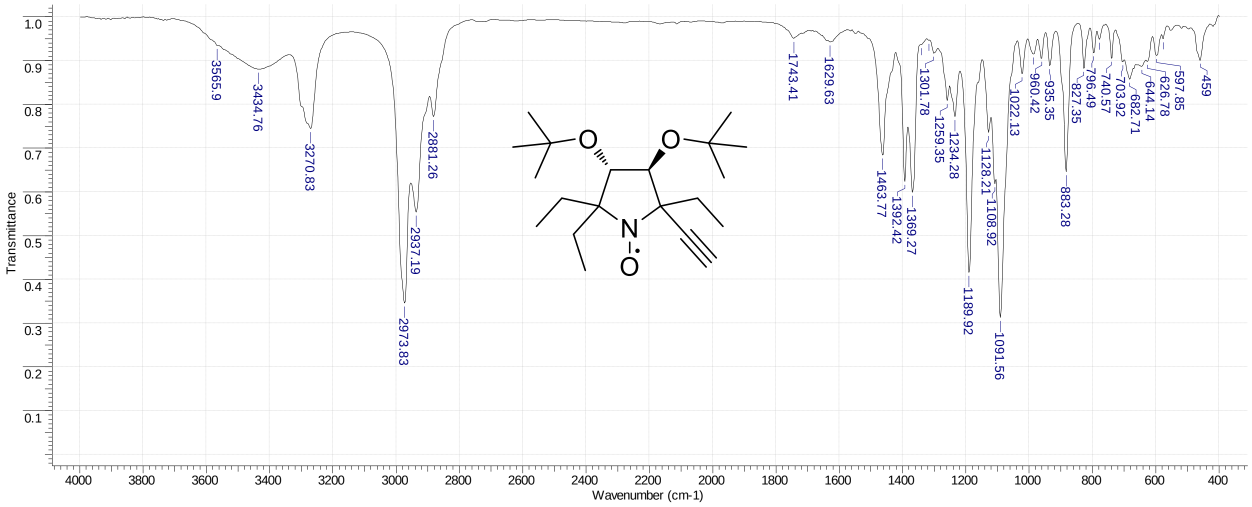

IR (KBr) of (3S,4S)-3,4-di-tert-butoxy-2,2,5,5-tetraethylpyrrolidine 1-oxyl (18)

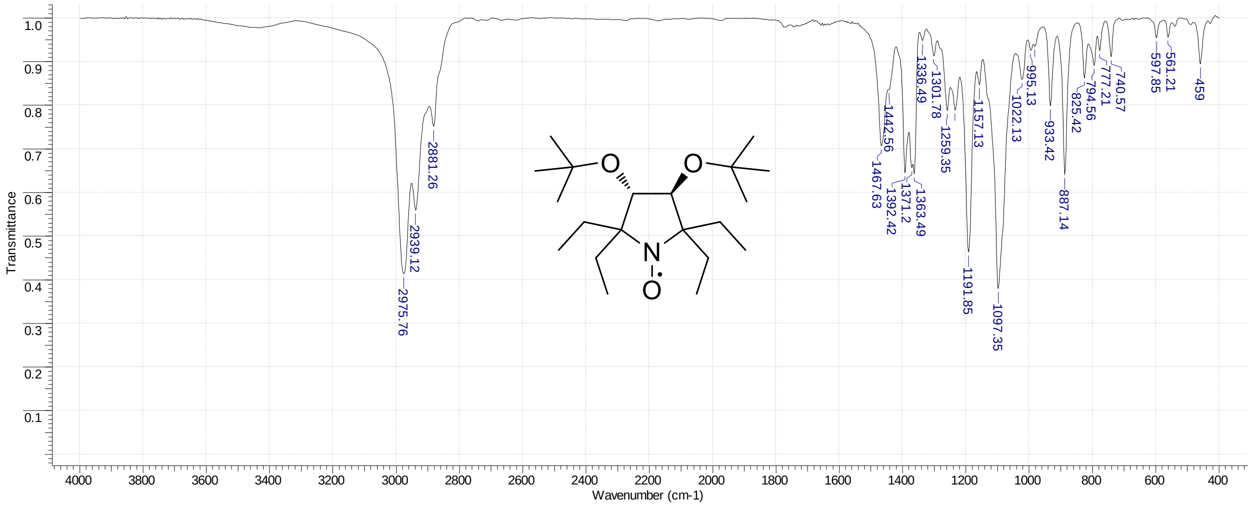

IR (neat) of 2,2,5-triethyl-5-(3-hydroxyprop-1-yn-1-yl)pyrrolidin-1-oxyl (23)

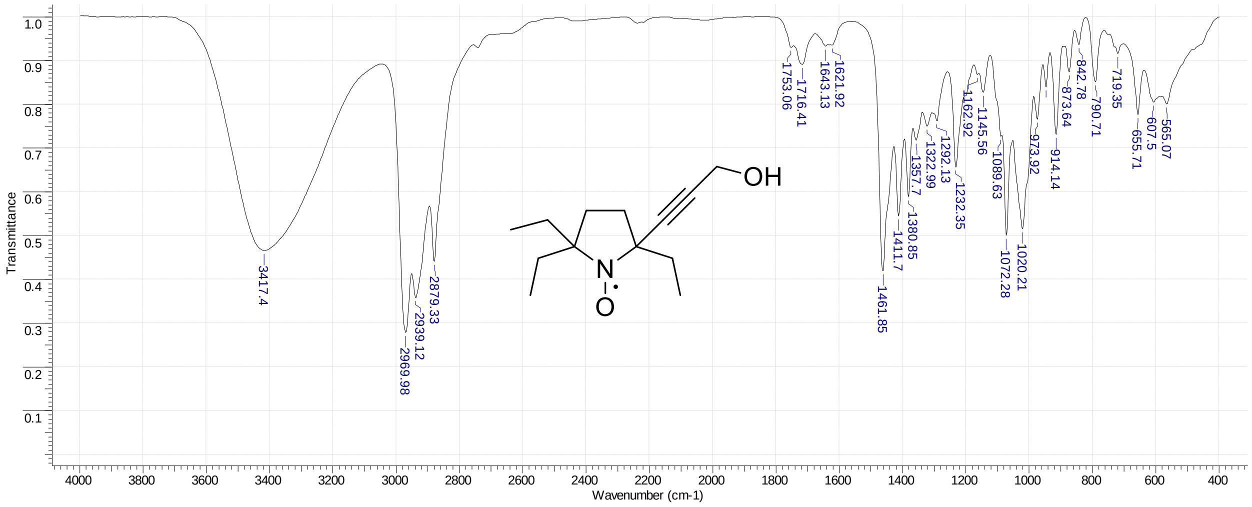

IR (KBr) of 2,2,5-triethyl-5-phenylethynyl-3,4-bis(hydroxymethyl)-pyrrolidine-1-oxyl (22a)

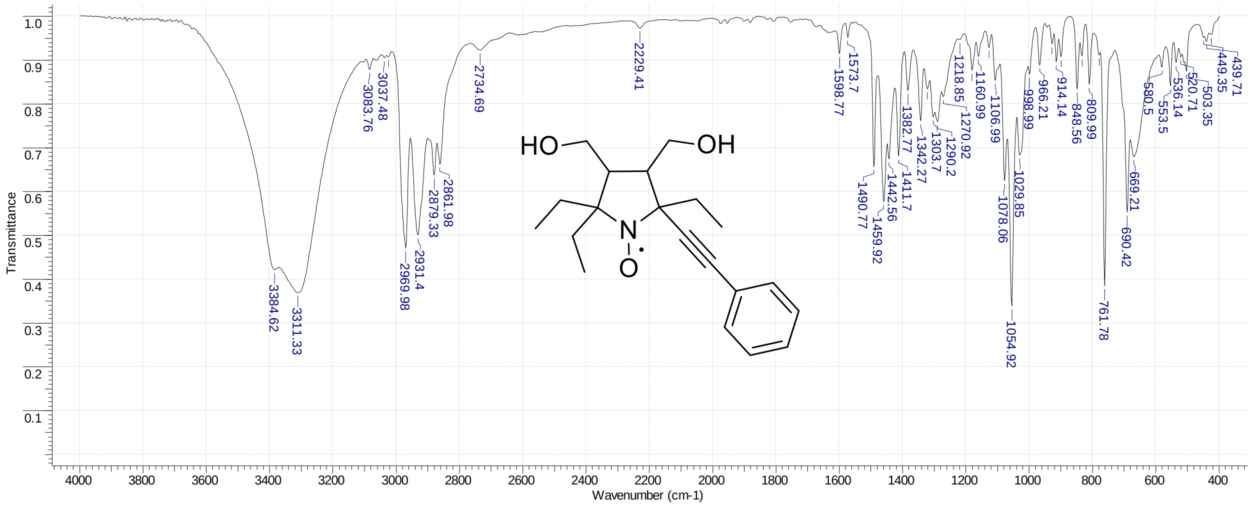

IR (KBr) of 2,2,5-triethyl-5-(3-hydroxyprop-1-yn-1-yl)-3,4-bis(hydroxymethyl)-pyrrolidine-1-oxyl (22b)

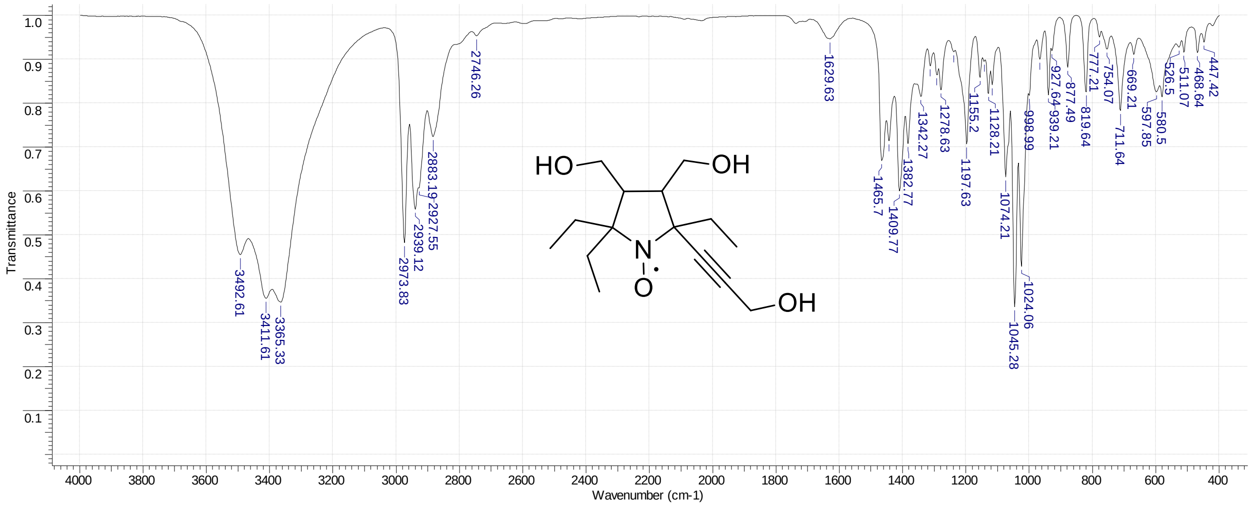

IR (KBr) of 2,2,5-triethyl-5-(3-hydroxy-3-methylbut-1-yn-1-yl)-3,4-bis(hydroxymethyl)-pyrrolidine-1-oxyl (22c)

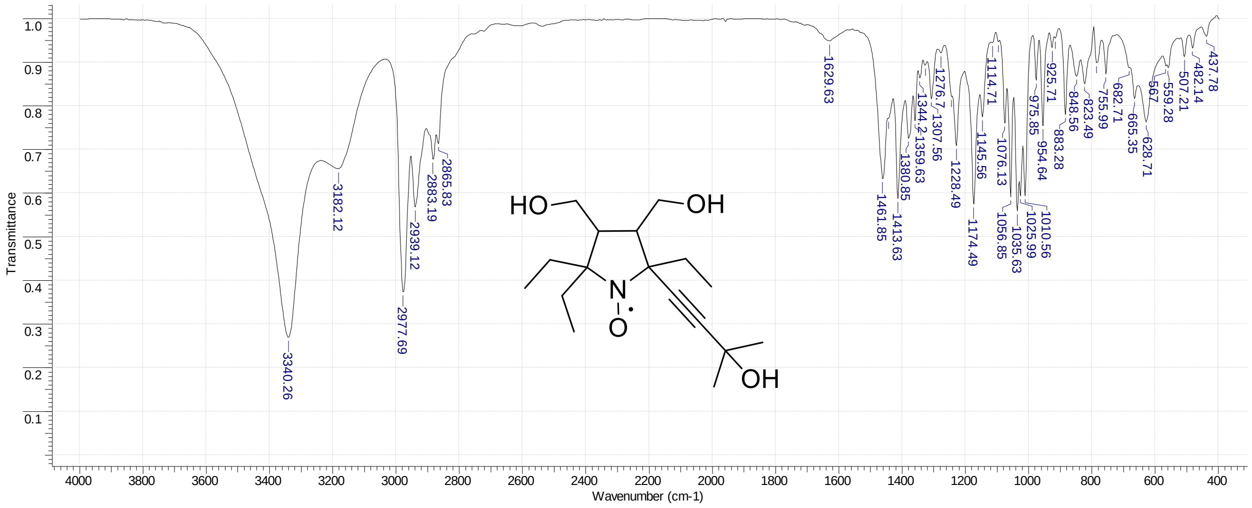

X-ray diffraction data for compounds

X-ray diffraction data for compounds **8d, 12a-c, 13b, 14, 22a-c** were obtained at room temperature with a Bruker Kappa Apex II CCD diffractometer using ϕ, ω scans of narrow frames with Mo-Kα radiation (λ = 0.71073 Å) and a graphite monochromator. The structures were solved by direct methods, and refined by the full-matrix least-squares method against all F^2 in the anisotropic approximation using the SHELX-2018 set of programs. The positions of hydroxyl H atoms were located from difference map and refined isotropically. Positions of the rest H atoms were refined isotropically with the riding model. Absorption corrections were applied empirically using the SADABS programs. Crystallographic data are listed in Table S1. CCDC 2209668 - 2209676 contain the supplementary crystallographic data for this paper. These data can be obtained free of charge from The Cambridge Crystallographic Data Centre https://www.ccdc.cam.ac.uk/structures/.

Figure S1. Structure of 8d (CCDC 2209668)

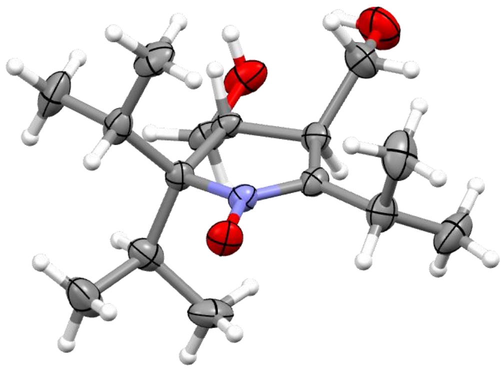

Figure S2. Structure of 12a (CCDC 2209669)

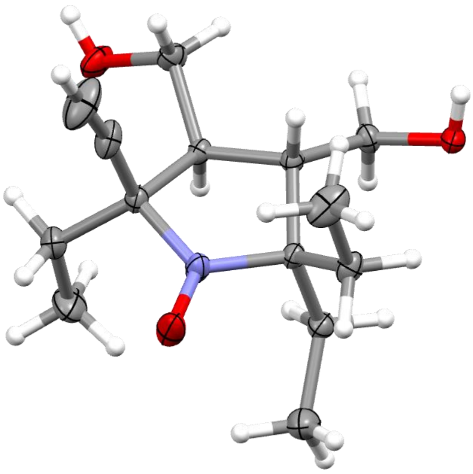

Figure S3. Structure of 12c (CCDC 2209670)

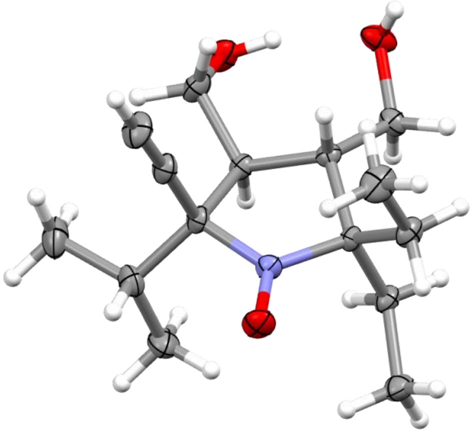

Figure S4. Structure of 12b (CCDC 2209671)

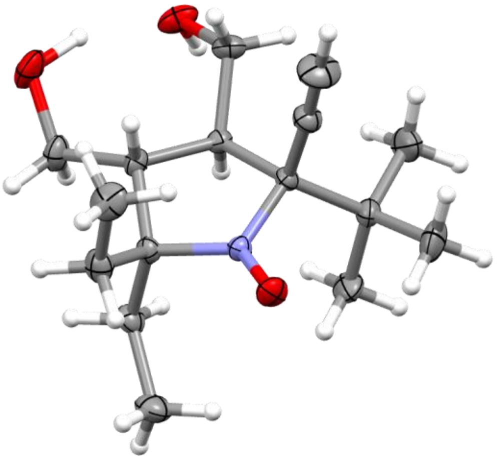

Figure S5. Structure of 13b (CCDC 2209672)

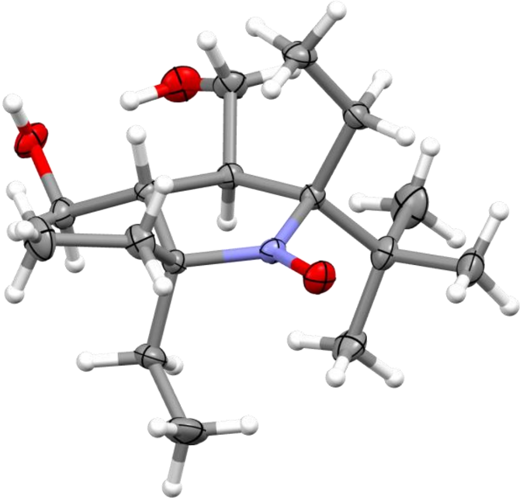

Figure 6S. Structure of 14 (CCDC 2209676)

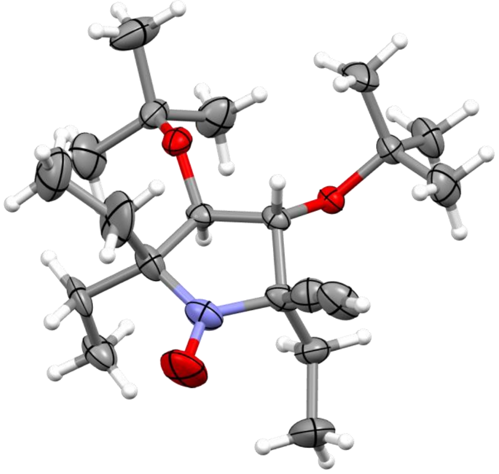

Figure S7. Structure of 22a (CCDC 2209673)

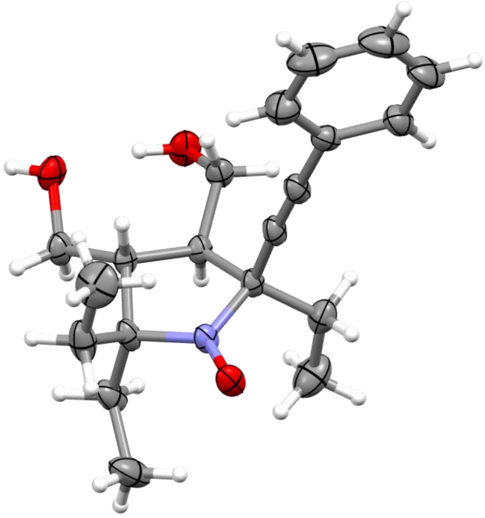

Figure S8. Structure of 22b (CCDC 2209674)

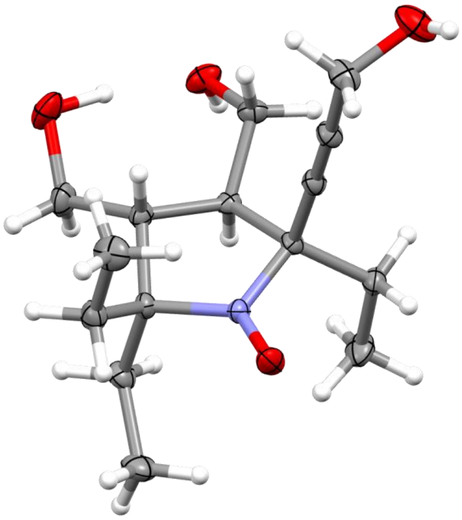

Figure S9. Structure of 22c (CCDC 2209675)

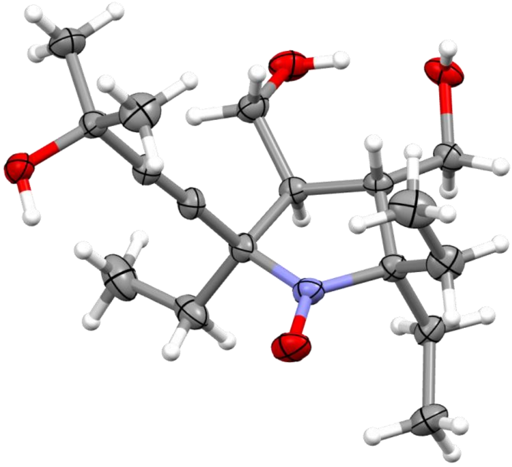

Table S1. Experimental details

	8c	12a	12c	12b
Crystal data				
Chemical formula	$C_{15}H_{29}NO_3$	$C_{14}H_{24}NO_3$	$C_{15}H_{26}NO_3$	$C_{16}H_{28}NO_3$
M_r	271.39	254.34	268.37	282.39
Crystal system, space group	Monoclinic, $P2_1/n$	Orthorhombic, $Pca2_1$	Orthorhombic, *Pbca*	Monoclinic, $P2_1/c$
a, *b*, *c* (Å)	13.3109 (12), 15.4649 (19), 16.3380 (19)	14.3478 (6), 7.9851 (3), 12.6962 (5)	14.7776 (6), 13.0092 (5), 15.8442 (7)	15.9755 (6), 7.7241 (3), 13.1380 (4)
α, β, γ (°)	90, 104.695 (4), 90	90, 90, 90	90, 90, 90	90, 90.635 (1), 90
V (Å^3)	3253.2 (6)	1454.59 (10)	3046.0 (2)	1621.08 (10)
Z	8	4	8	4
μ (mm^{-1})	0.08	0.08	0.08	0.08
Crystal size (mm)	0.61 × 0.15 × 0.08	0.54 × 0.28 × 0.27	0.59 × 0.21 × 0.19	0.55 × 0.35 × 0.18
Data collection				
Absorption correction	Multi-scan *SADABS2016*/2	Multi-scan *SADABS2008*/1	Multi-scan *SADABS2016*/2	Multi-scan *SADABS2008*/1
T_{min}, T_{max}	0.839, 0.928	0.918, 0.954	0.893, 0.954	0.913, 0.956
No. of measured, independent and observed [$I > 2\sigma(I)$] reflections	52393, 5794, 3964	48142, 3946, 3619	52165, 3030, 2461	33407, 4383, 3654
R_{int}	0.052	0.037	0.047	0.045
$(\sin \theta/\lambda)_{max}$ (Å^{-1})	0.597	0.687	0.619	0.687
Refinement				
$R[F^2 > 2\sigma(F^2)]$, $wR(F^2)$, S	0.089, 0.271, 1.03	0.051, 0.139, 0.97	0.038, 0.116, 1.02	0.048, 0.158, 1.06
No. of reflections	5794	3946	3030	4383
No. of parameters	365	175	182	192
No. of restraints	6	3	0	0
H-atom treatment	H atoms treated by a mixture of independent and constrained refinement	H atoms treated by a mixture of independent and constrained refinement	H atoms treated by a mixture of independent and constrained refinement	H atoms treated by a mixture of independent and constrained refinement
$\Delta\rangle_{max}$, $\Delta\rangle_{min}$ (e Å^{-3})	0.82, -0.35	0.55, -0.23	0.21, -0.14	0.29, -0.18
Absolute structure	–	Refined as an inversion twin.	–	–
Absolute structure parameter	–	0.4 (16)	–	–
CCDC number	2209668	2209669	2209670	2209671

	**13b**	**22a**	**22b**	**22c**
Crystal data				
Chemical formula	$C_{16}H_{32}NO_3$	$C_{20}H_{28}NO_3$	$C_{15}H_{28}NO_4$	$C_{17}H_{30}NO_4$
M_r	286.42	330.43	286.38	312.42
Crystal system, space group	Monoclinic, $P2_1/n$	Monoclinic, $C2/c$	Monoclinic, $P2_1/c$	Monoclinic, $P2_1/c$
a, *b*, *c* (Å)	9.1995 (4), 15.7453 (6), 12.1584 (5)	30.2701 (17), 9.1483 (6), 15.4728 (9)	14.6526 (15), 7.5382 (8), 13.8807 (13)	9.1957 (5), 16.7092 (8), 12.5715 (6)
α, β, γ (°)	90, 107.388 (2), 90	90, 114.717 (2), 90	90, 94.886 (4), 90	90, 106.5453 (17), 90
V (Å^3)	1680.65 (12)	3892.2 (4)	1527.6 (3)	1851.67 (16)
Z	4	8	4	4
μ (mm^{-1})	0.08	0.08	0.09	0.08
Crystal size (mm)	0.61 × 0.59 × 0.30	0.59 × 0.19 × 0.14	0.28 × 0.28 × 0.04	0.49 × 0.27 × 0.19
Data collection				
Absorption correction	Multi-scan *SADABS2008*/1	Multi-scan *SADABS2016*/2	Multi-scan *SADABS2016*/2	Multi-scan *SADABS2016*/2
T_{min}, T_{max}	0.859, 0.914	0.895, 0.971	0.813, 0.901	0.900, 0.940
No. of measured, independent and observed [$I > 2\sigma(I)$] reflections	29318, 4165, 3370	9180, 3454, 2462	20737, 2704, 2236	16062, 4481, 3218
R_{int}	0.044	0.019	0.061	0.059
(sin θ/λ)$_{max}$ (Å^{-1})	0.667	0.596	0.597	0.661
Refinement				
$R[F^2 > 2\sigma(F^2)]$, $wR(F^2)$, S	0.053, 0.152, 1.06	0.050, 0.171, 1.00	0.052, 0.157, 1.05	0.057, 0.167, 1.03
No. of reflections	4165	3454	2704	4481
No. of parameters	195	228	191	216
No. of restraints	2	0	1	3
H-atom treatment	H atoms treated by a mixture of independent and constrained refinement	H atoms treated by a mixture of independent and constrained refinement	H atoms treated by a mixture of independent and constrained refinement	H atoms treated by a mixture of independent and constrained refinement
Δ〉$_{max}$, Δ〉$_{min}$ (e Å^{-3})	0.31, -0.20	0.28, -0.17	0.70, -0.31	0.24, -0.18
Absolute structure	–	–	–	–
Absolute structure parameter	–	–	–	–
CCDC number	2209672	2209673	2209674	2209675

	**14**
Crystal data	
Chemical formula	$C_{20}H_{33}NO_2$
M_r	319.47
Crystal system, space group	Orthorhombic, $P2_12_12_1$
a, *b*, *c* (Å)	9.4853 (4), 11.3183 (6), 19.8743 (10)
α, β, γ (°)	90, 90, 90
V (Å^3)	2133.65 (18)
Z	4
μ (mm^{-1})	0.06
Crystal size (mm)	0.59 × 0.48 × 0.32
Data collection	
Absorption correction	Multi-scan<br>*SADABS2008*/1
T_{min}, T_{max}	0.916, 0.992
No. of measured, independent and observed [$I > 2\sigma(I)$] reflections	22118, 4190, 3184
R_{int}	0.052
$(\sin \theta/\lambda)_{max}$ (Å^{-1})	0.618
Refinement	
$R[F^2 > 2\sigma(F^2)]$, $wR(F^2)$, S	0.074, 0.219, 1.02
No. of reflections	4190
No. of parameters	225
No. of restraints	0
H-atom treatment	H-atom parameters constrained
$\Delta\rangle_{max}$, $\Delta\rangle_{min}$ (e Å^{-3})	0.28, -0.21
Absolute structure	Flack x determined using 1078 quotients [(I+)-(I-)]/[(I+)+(I-)] (Parsons, Flack and Wagner, Acta Cryst. B69 (2013) 249-259).
Absolute structure parameter	0.6 (4)
CCDC number	2209676

Computer programs: Bruker *APEX2*, Bruker *SAINT*, *SHELXT2018*/2 (Sheldrick, 2018), *SHELXL2018*/3 (Sheldrick, 2018), Bruker *SHELXTL*.

EPR

EPR of 2,2,5-triethyl-5-ethynyl-3,4-bis(hydroxymethyl)-pyrrolidine-1-oxyl (12a)

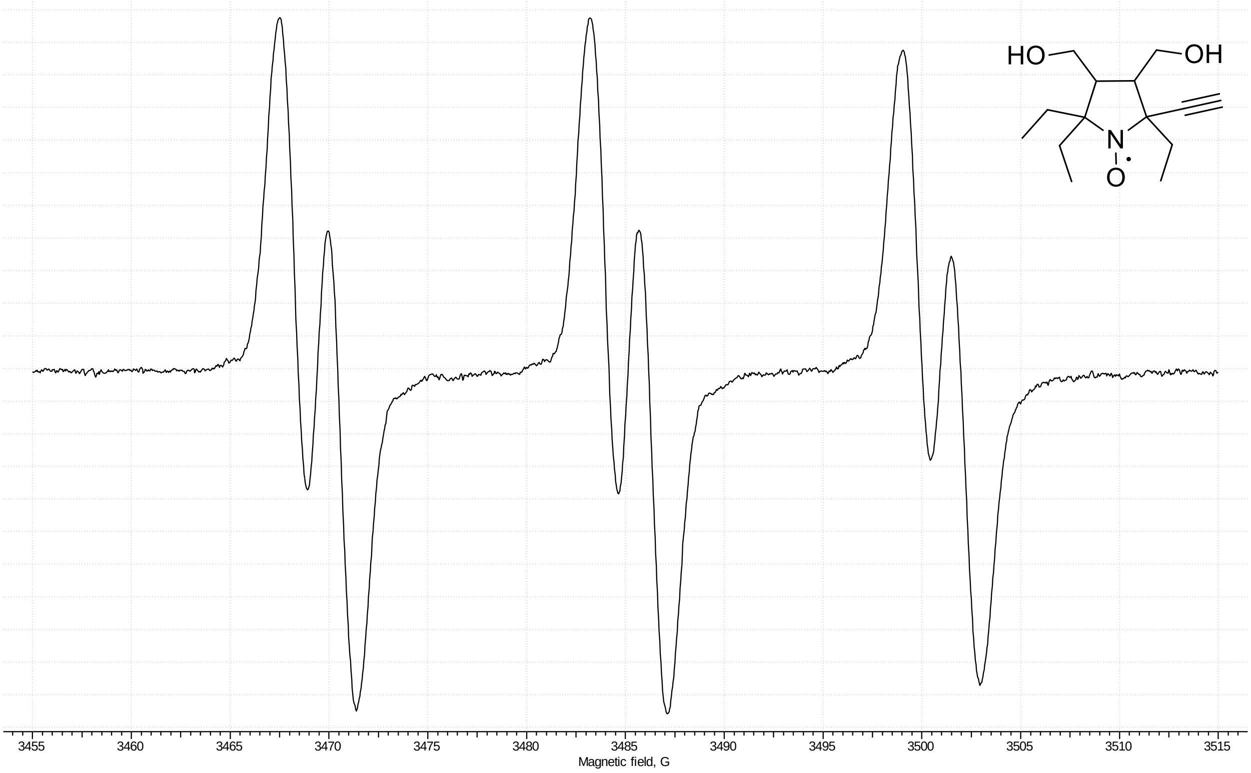

EPR of 2,2-diethyl-5-isopropyl-5-ethynyl-3,4-bis(hydroxymethyl)-pyrrolidine-1-oxyl (12c)

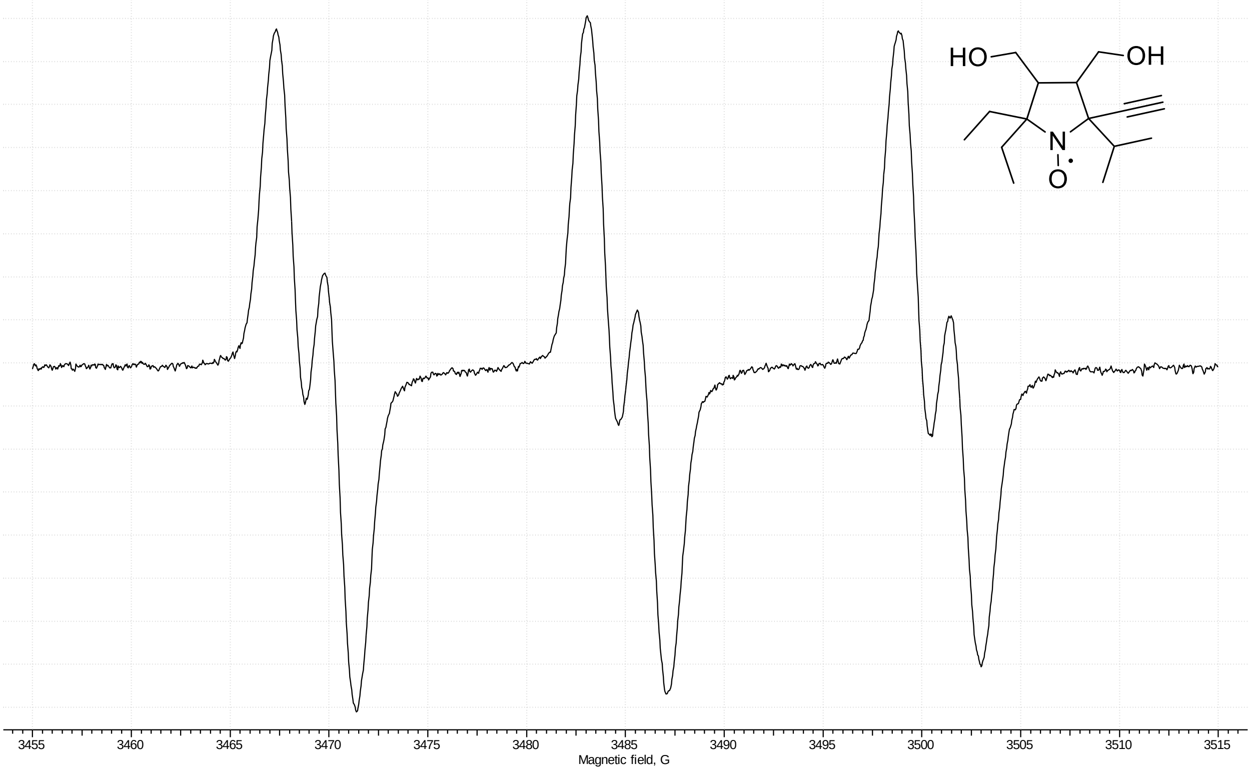

EPR of 5-tert-dutyl-2,2-diethyl-5-ethynyl-3,4-bis(hydroxymethyl)-pyrrolidine-1-oxyl (12b)

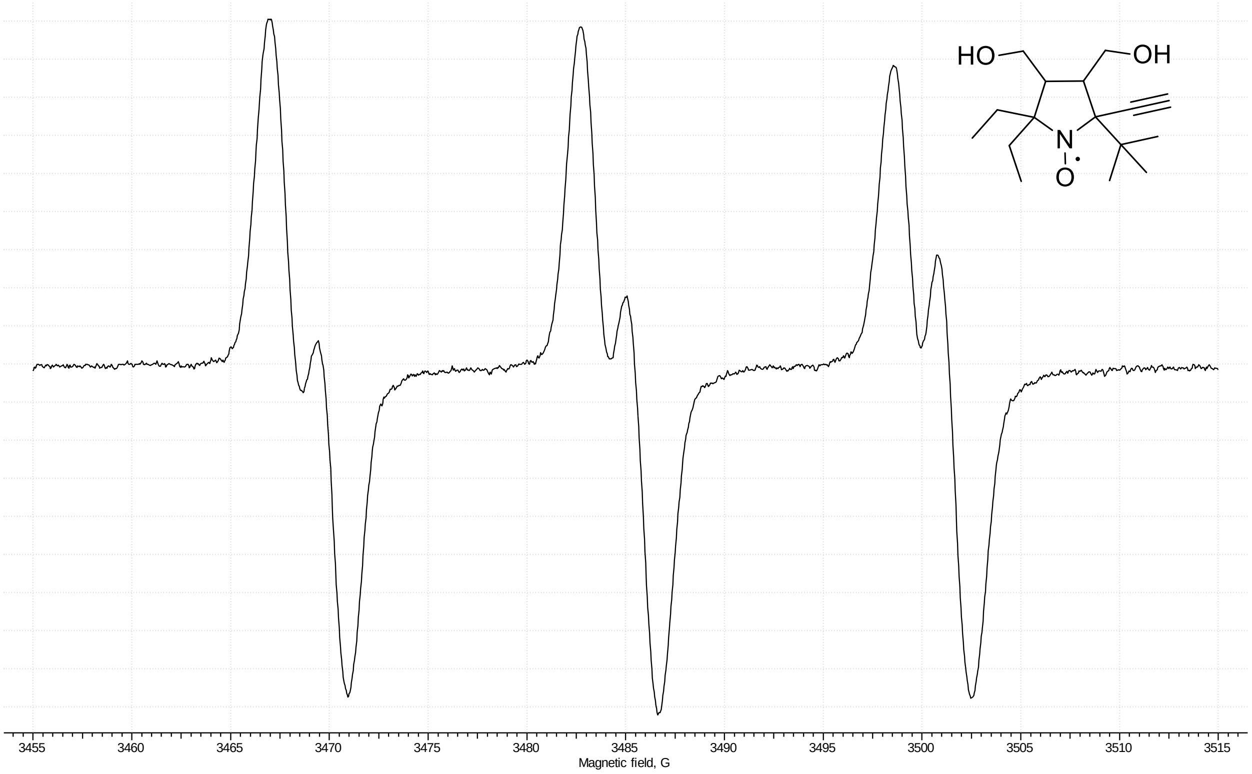

EPR of 2,5,5-triethyl-2-ethynyl-4-pyrrolidino-2,5-dihydroimidazol-1-oxyl (17)

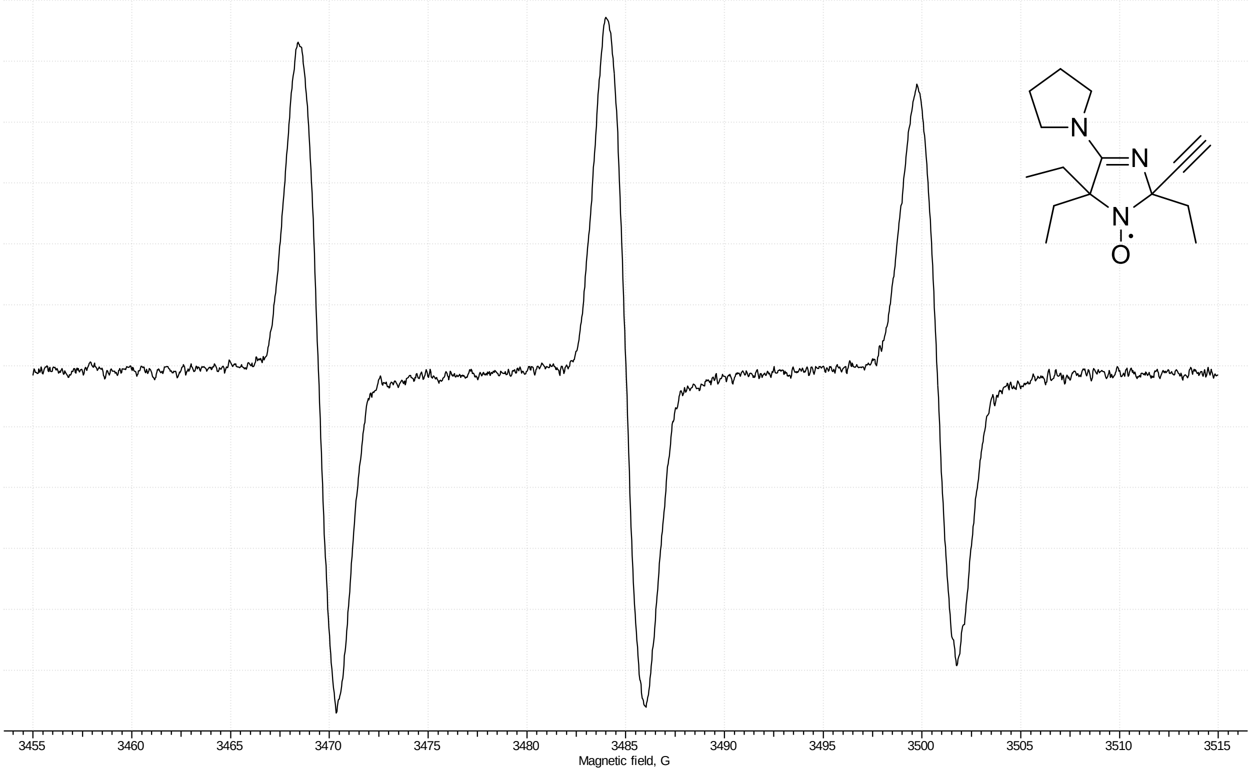

EPR of 2,2,5-triethyl-5-ethynyl-3,4,4-trimethylimidazolidin-1-oxyl (16)

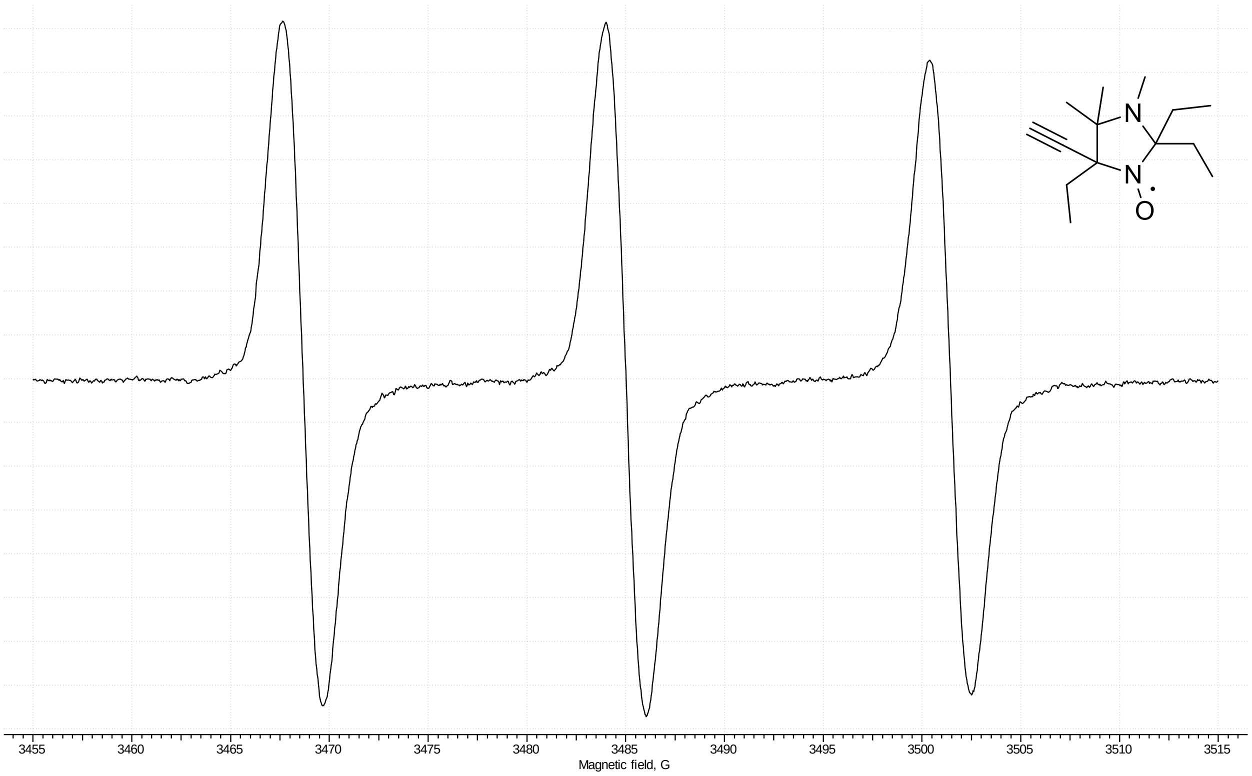

EPR of 2,2,5-triethyl-5-ethynylpyrrolidin-1-oxyl (15)

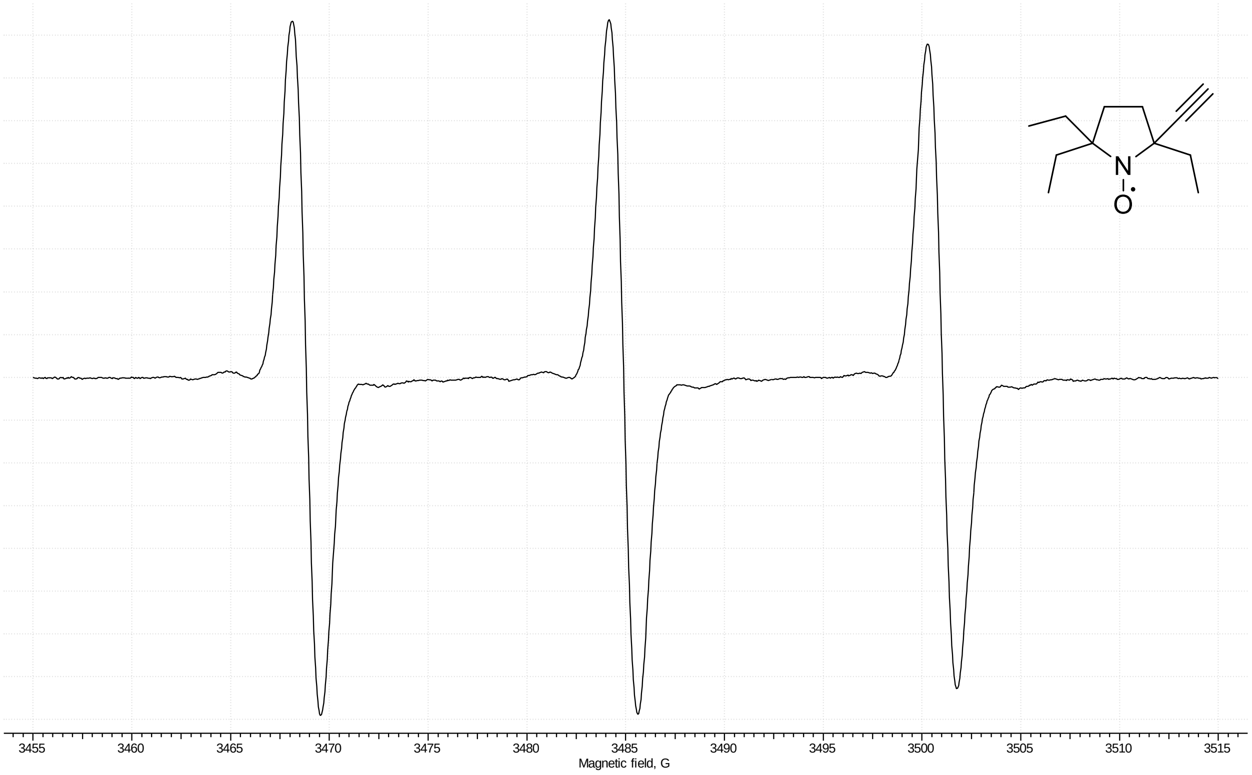

EPR of 2,2,5,5-tetraethyl-3,4-bis(hydroxymethyl)-pyrrolidine-1-oxyl (13a)

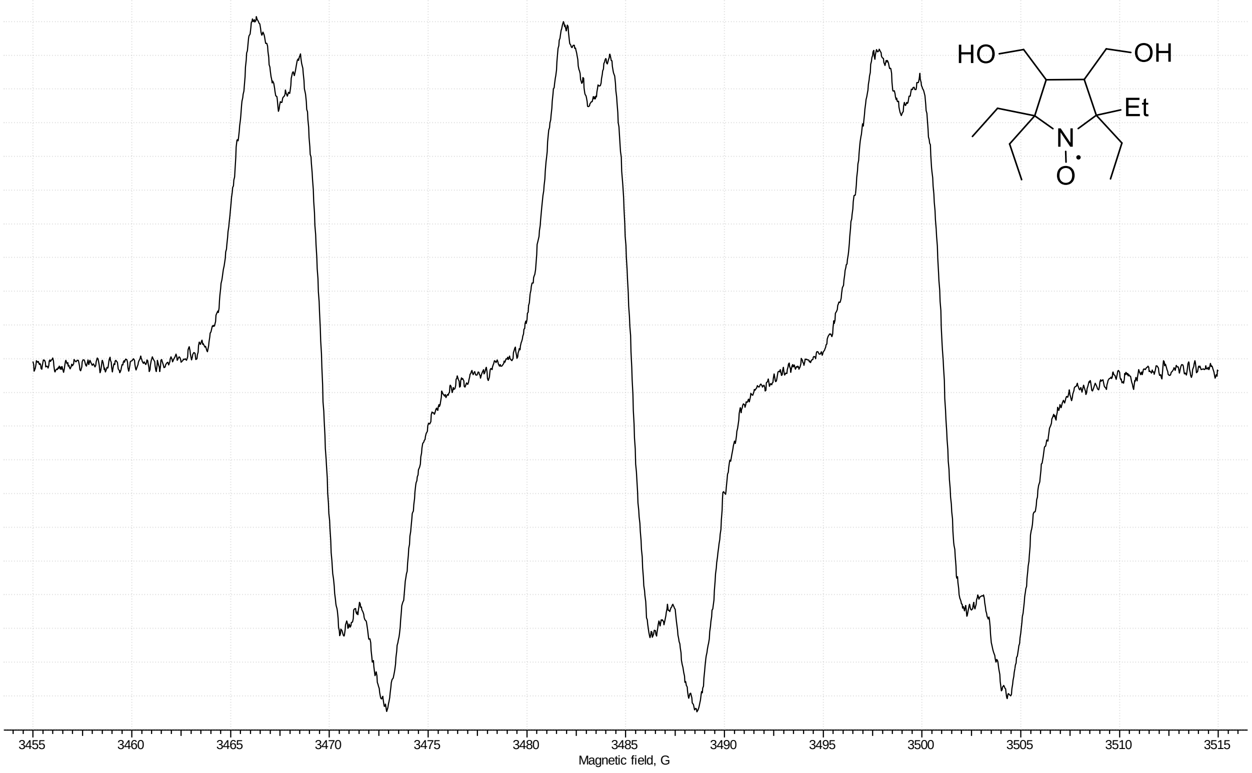

EPR of 2,2,5-triethyl-5-isopropyl-3,4-bis(hydroxymethyl)-pyrrolidine-1-oxyl (13c)

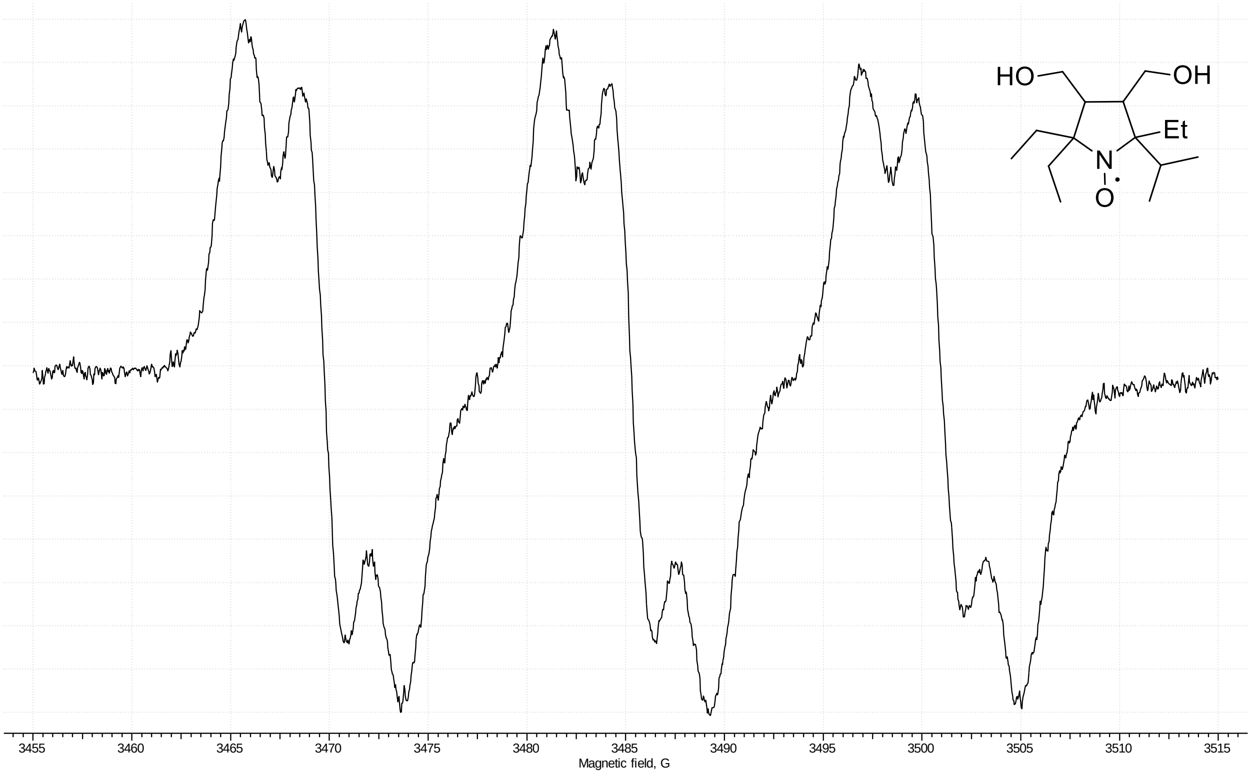

EPR of 2,2,5-triethyl-5-tert-butyl-3,4-bis(hydroxymethyl)-pyrrolidine-1-oxyl (13b)

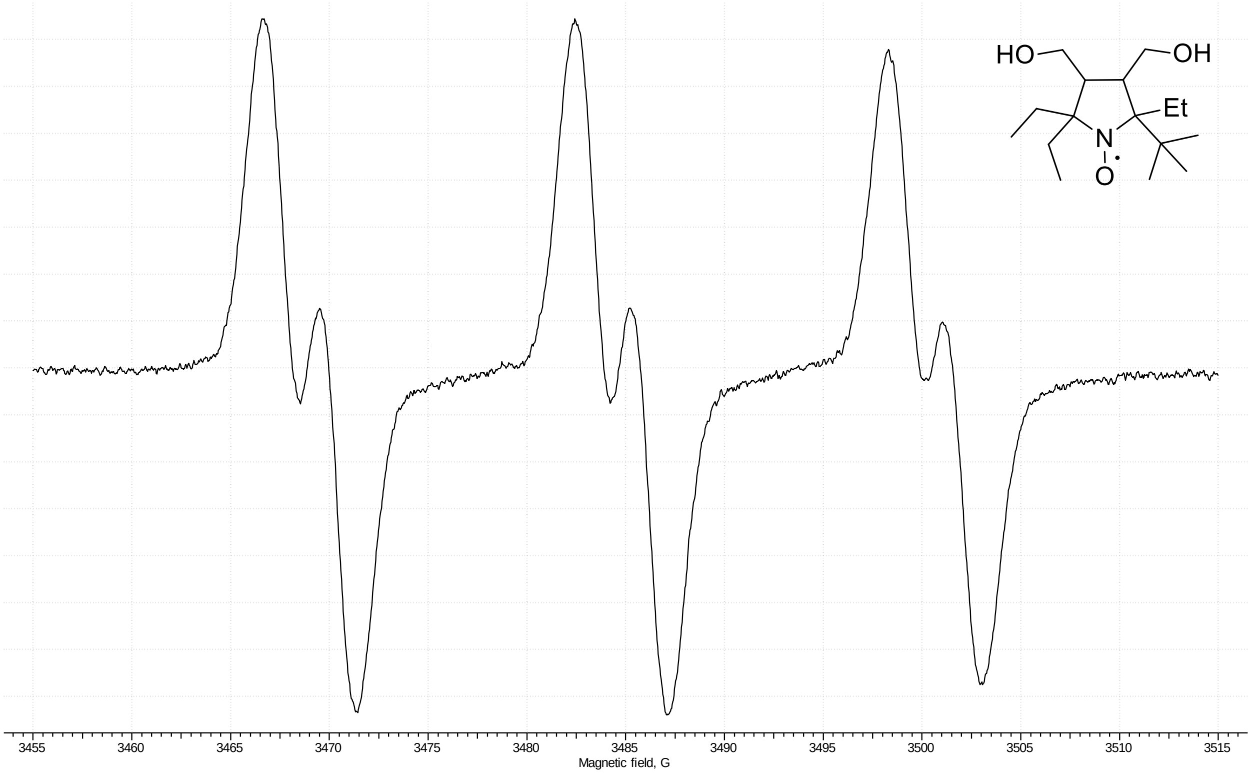

EPR of 2,2,5,5-tetraethyl-4-pyrrolidino-2,5-dihydroimidazol-1-oxyl (21)

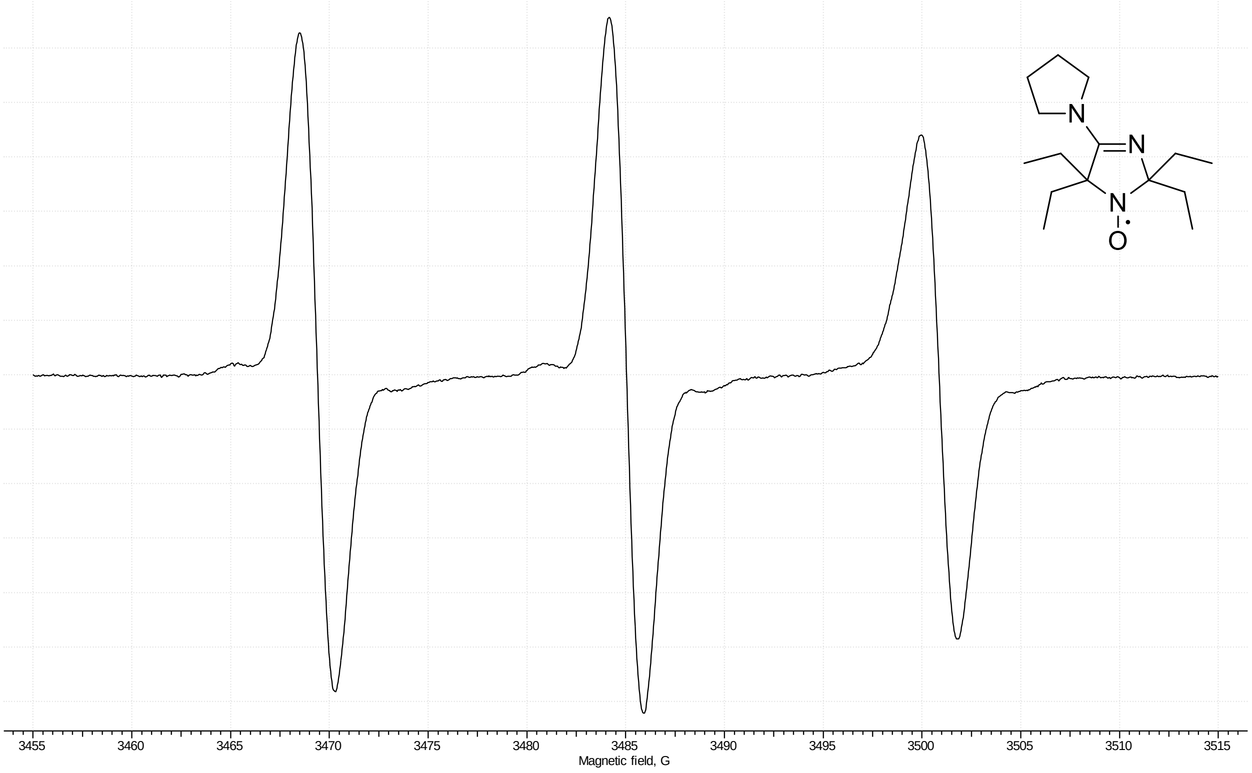

EPR of 2,2,5,5-tetraethyl-3,4,4-trimethylimidazolidin-1-oxyl (20)

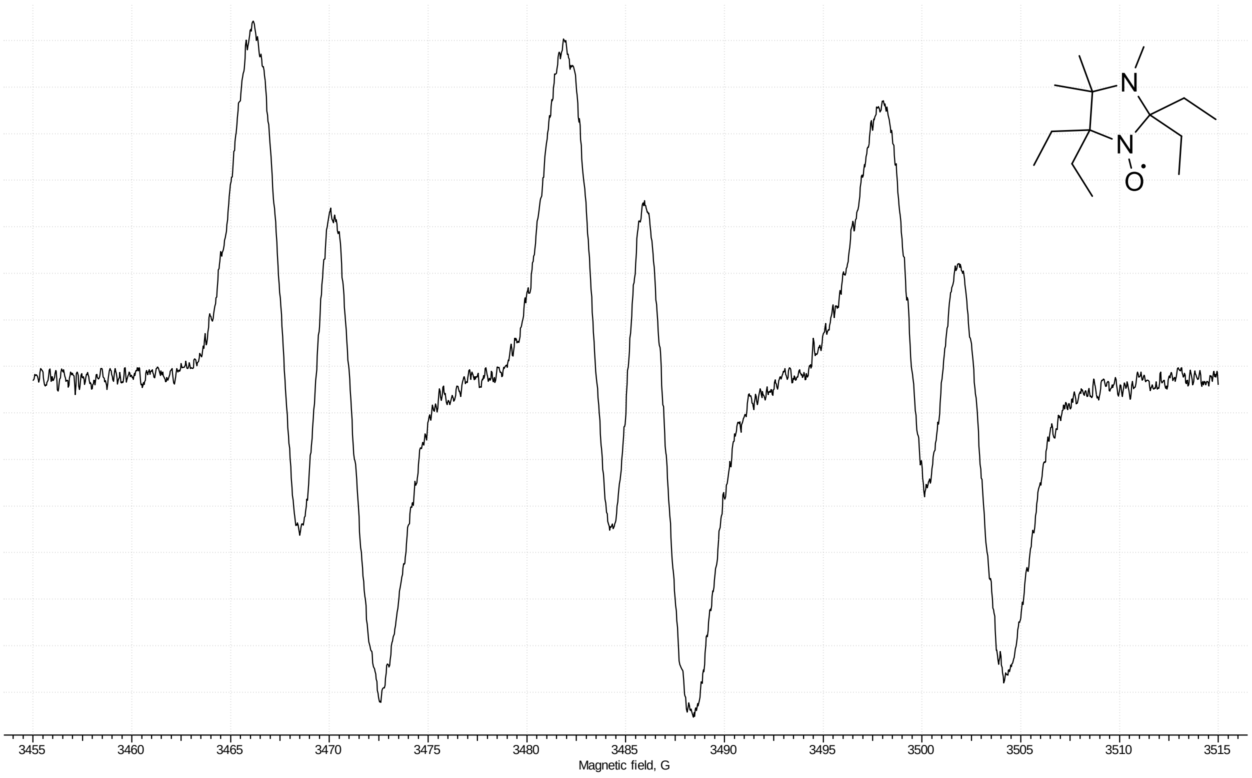

EPR of 2,2,5,5-tetraethylpyrrolidin-1-oxyl (19)

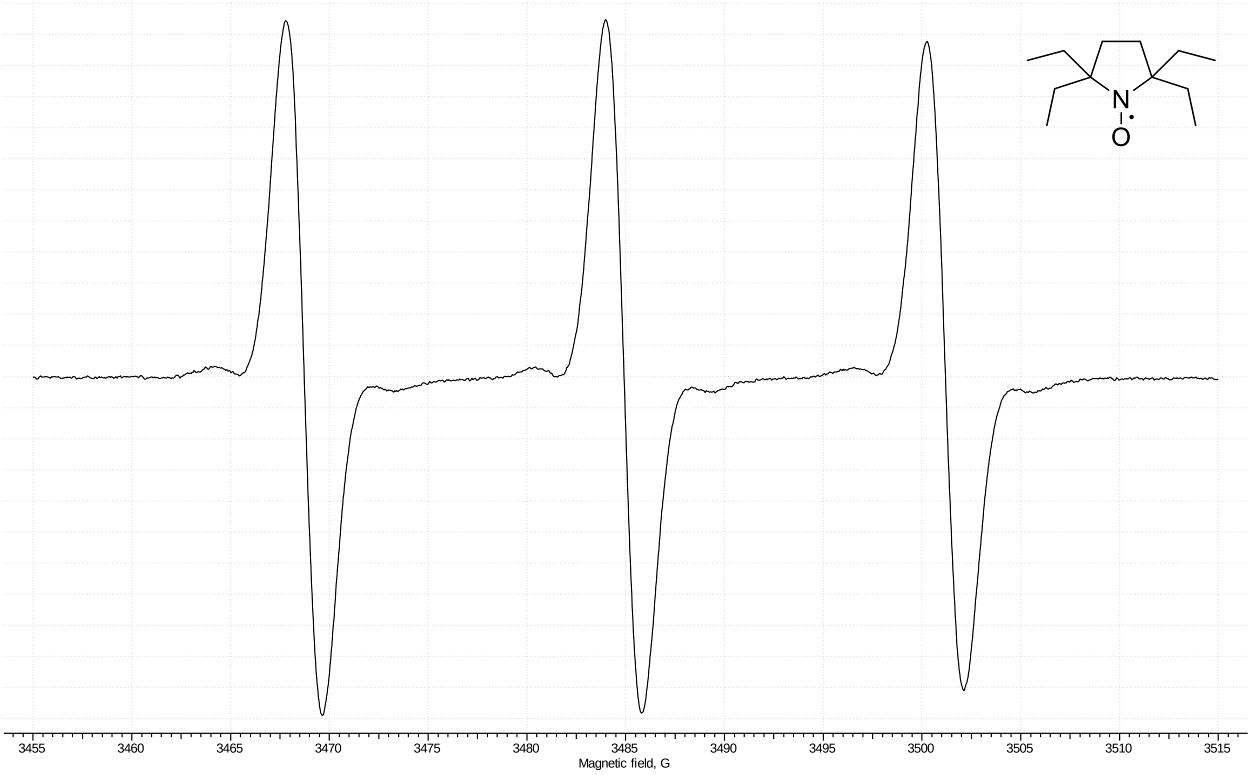

EPR of (3S,4S,5S)-3,4-di-tert-butoxy-2,2,5-triethyl-5-ethynylpyrrolidine 1-oxyl (14)

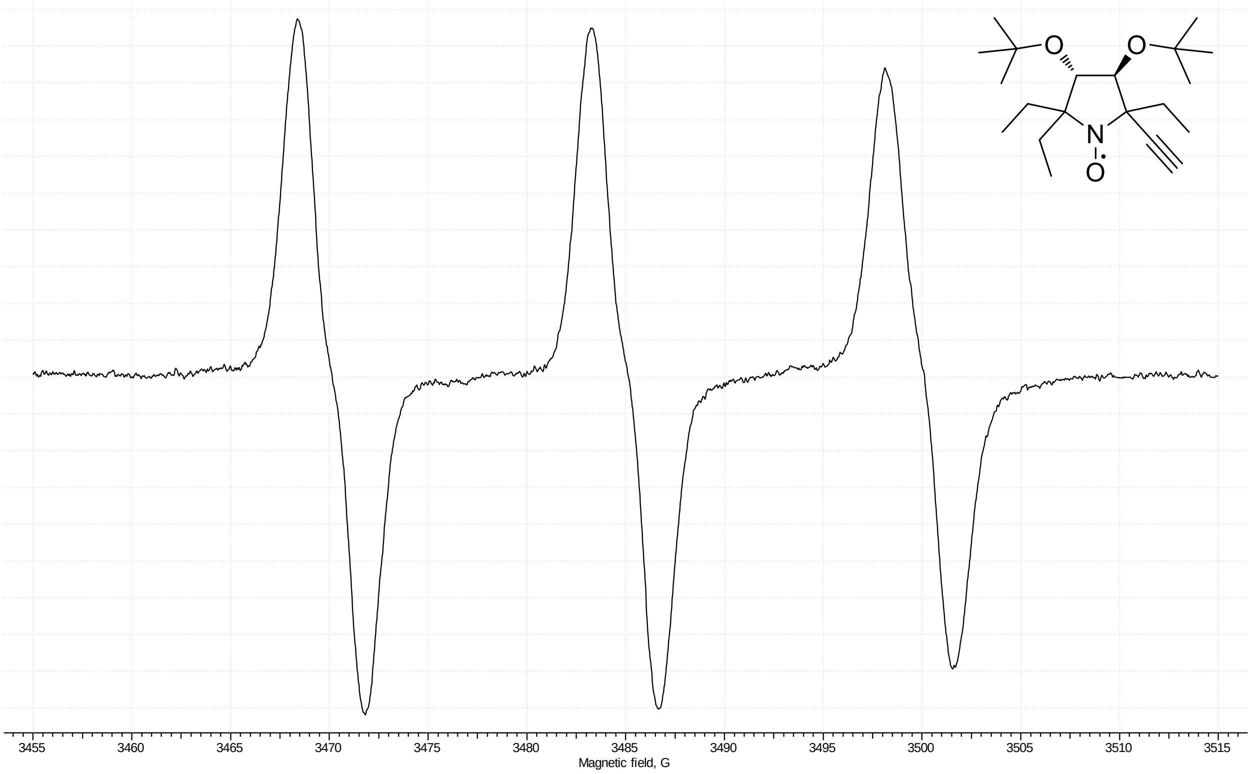

EPR of (3S,4S)-3,4-di-tert-butoxy-2,2,5,5-tetraethylpyrrolidine 1-oxyl (18)

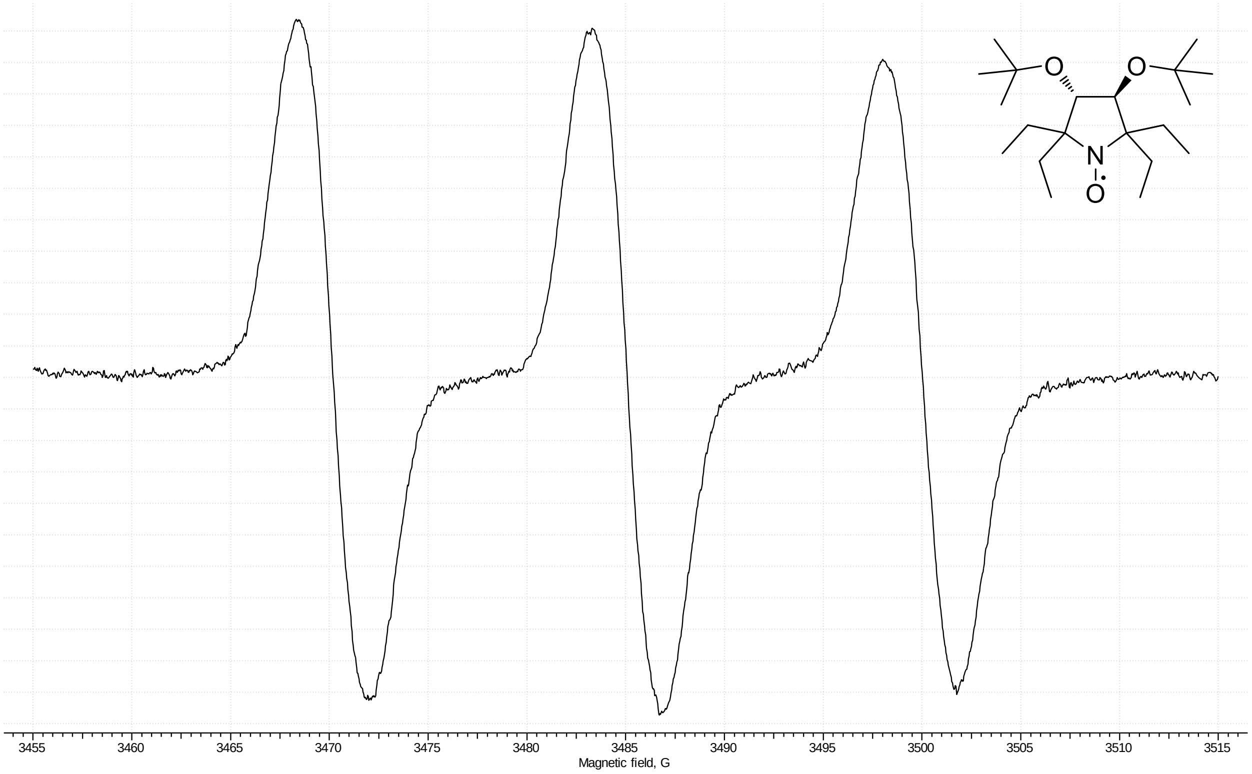

EPR of 2,2,5-triethyl-5-(3-hydroxyprop-1-yn-1-yl)pyrrolidin-1-oxyl (23)

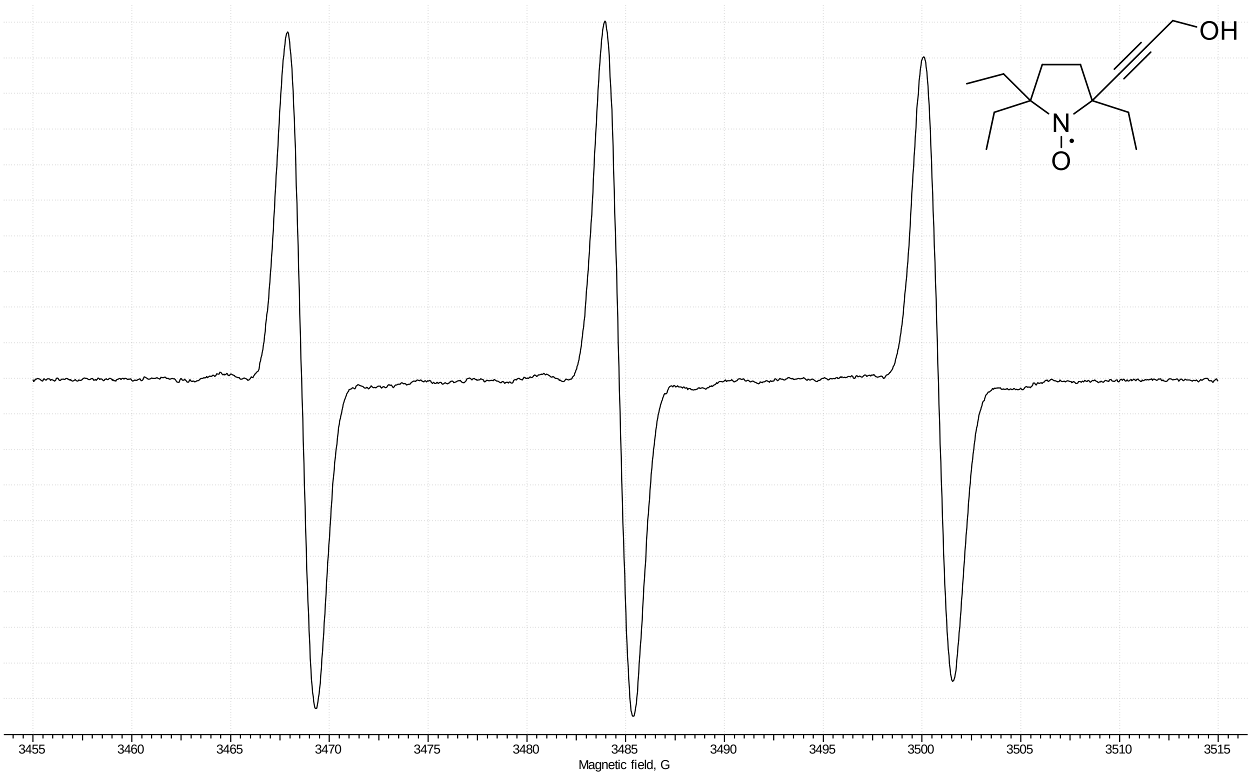

EPR of 2,2,5-triethyl-5-phenylethynyl-3,4-bis(hydroxymethyl)-pyrrolidine-1-oxyl (22a)

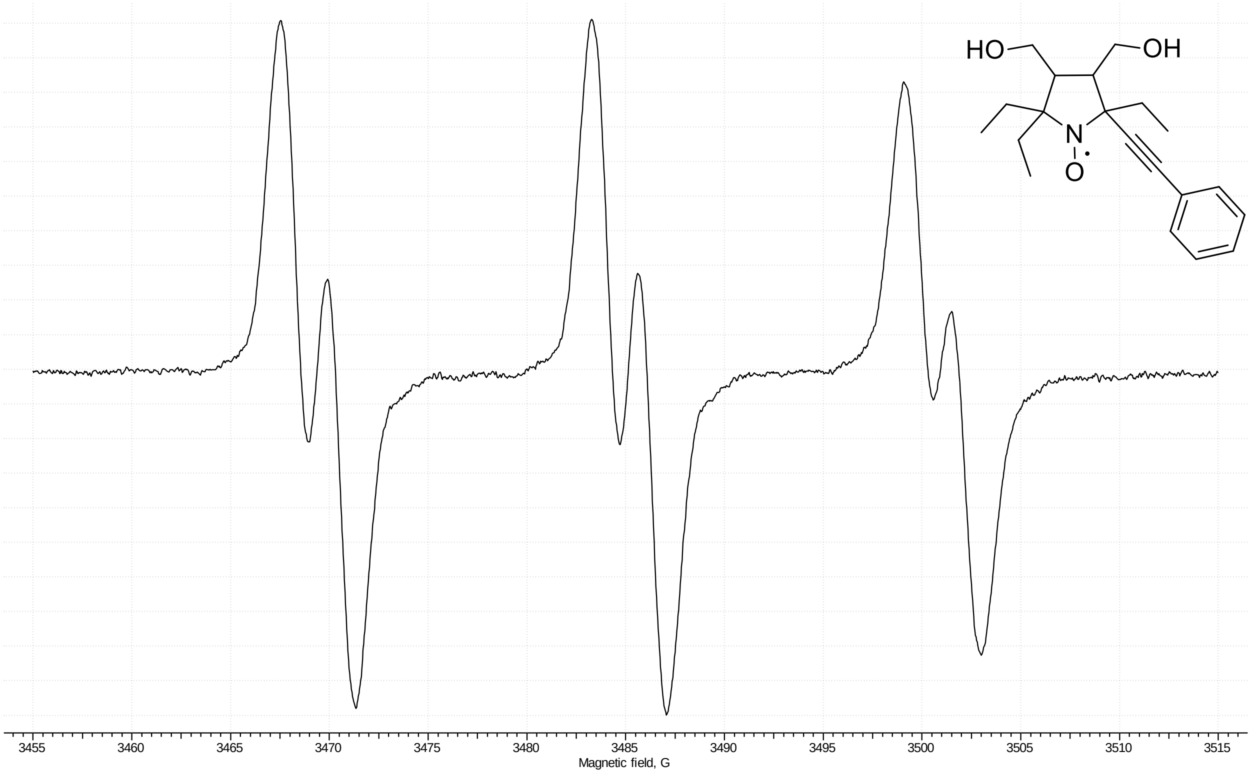

EPR of 2,2,5-triethyl-5-(3-hydroxyprop-1-yn-1-yl)-3,4-bis(hydroxymethyl)-pyrrolidine-1-oxyl (22b)

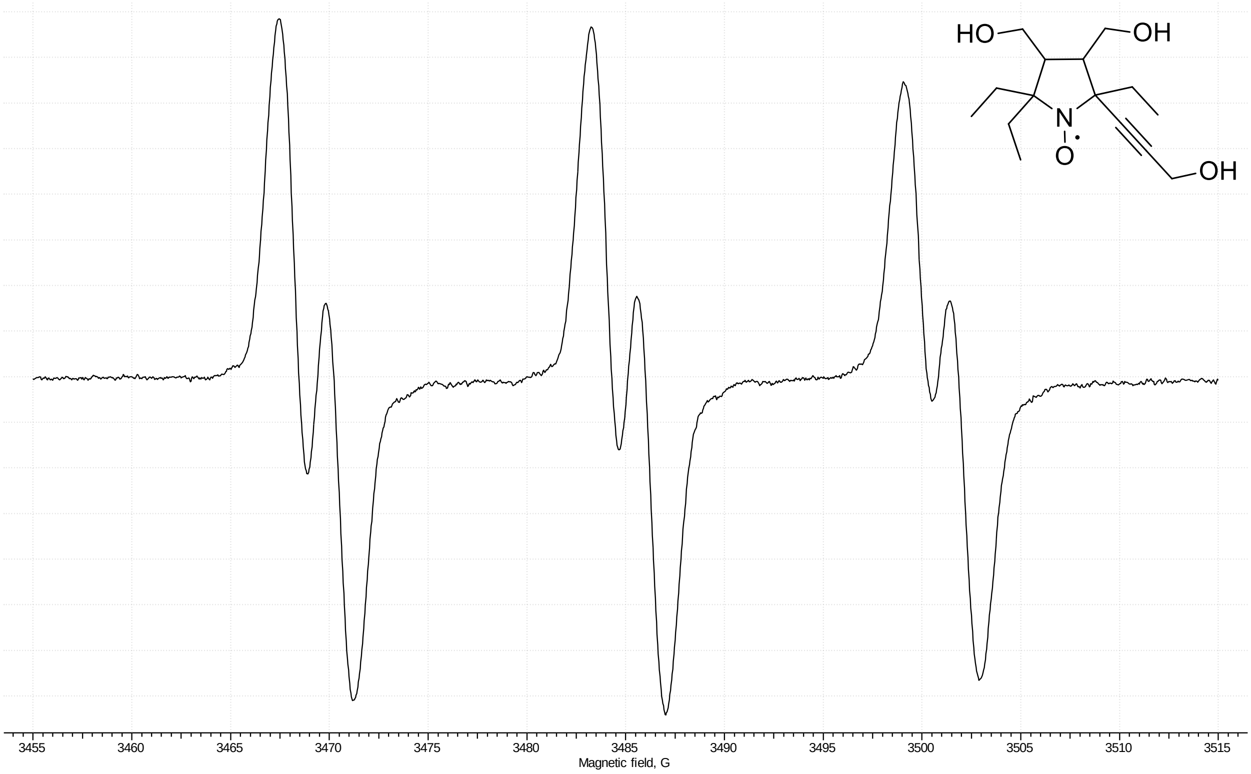

EPR of 2,2,5-triethyl-5-(3-hydroxy-3-methylbut-1-yn-1-yl)-3,4-bis(hydroxymethyl)-pyrrolidine-1-oxyl (22c)

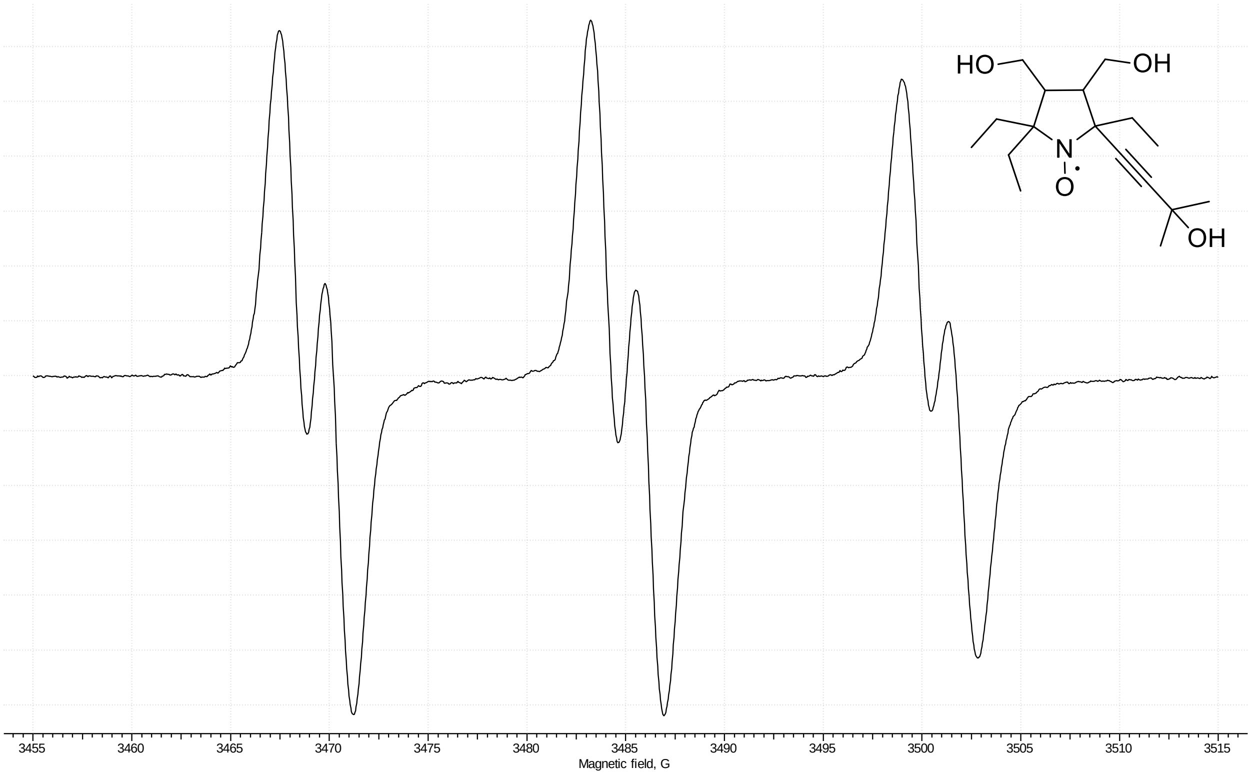

HPLC

HPLC of (3S,4S,5S)-3,4-di-tert-butoxy-2,2,5-triethyl-5-ethynylpyrrolidine 1-oxyl (14)

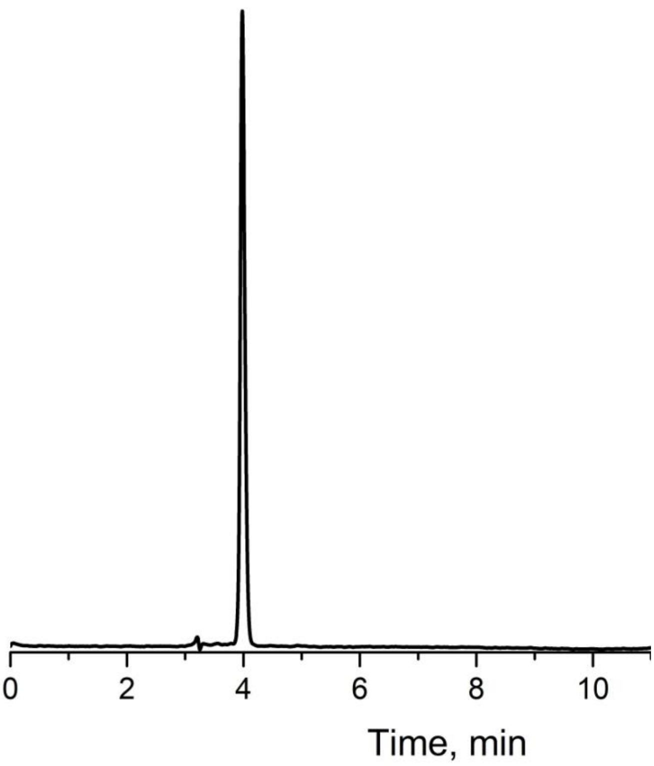

Supplement: Supplementary file 1 [file molecules-27-07626-s001.zip › molecules-1978872-supplementary-new version.pdf]
